# Supplementary material for: External injuries, trauma and avoidable deaths in Agincourt, South Africa: a retrospective observational and qualitative study
Source: BMJ Open. 2019 Jun 4;9(6):e027576. doi: 10.1136/bmjopen-2018-027576 (PMC6561452; doi:10.1136/bmjopen-2018-027576)
Supplement: Supplementary data [file bmjopen-2018-027576supp001.pdf]

Appendices

Appendix 1. Details of the definitions of injury survivability

DEFINITIONS OF SURVIVABILITY	STUDIES EXAMINING INJURY SURIVABILITY
Vitals at the scene of injury e.g. palpable pulse or blood pressure	[1]
Awake at the scene	[1-3]
Reversible or treatable injury	[4,3]
Generally stable at initial treatment or if unstable, becomes stable with initial treatment	[5]
Non-severe injuries	[3]
Non-severe single-system or single-organ or single-vessel injuries	[1,2]
Non-severe non-central nervous system (CNS) injuries	[3]
**APPROACHES TO CRITERIA FOR DEFINITION OF SURVIVABILITY**	**STUDIES**
Abbreviated Injury Scale (AIS)/Injury Severity Scores (ISS)/Probability of Survival (PS)/Trauma Score (TS)	[6-8]
AIS/ISS/PS and specific preventable factors	[9,10-12]
AIS/ISS/PS and impact of care delivered	[13-31]
Survivable injuries (subjective/panel)	[5]
Survivable injuries (subjective/panel) and impact of care delivered	[2,4,32-34,31,1,5,35,36,3]
Impact of care delivered	[37-38]

Appendix 2. Details of the three-delays model for care extracted from literature review*

DELAY	FACTORS AFFECTING UTILIZATION OR OUTCOME
DECIDING TO SEEK CARE	**Socio-economic/Cultural**<br>Illness factors<br>● Healthcare literacy<br>● Delayed discovery [4,14]<br>● Perceived severity<br>● Perceived etiology<br>Economic status [39]<br>Educational status
	**Perceived Accessibility**<br>Distance<br>Transportation [4]<br>● EMS versus personal transport**<br>● Road conditions<br>● Season—dry or rainy<br>Costs [39]<br>● Transportation<br>● Medical care<br>● Medications<br>● Opportunity costs<br>● Lost income<br>EMS transport protocols [39]<br>● Closest hospital versus best equipped hospital
	**Perceived quality of care**<br>Reputation or previous experience<br>Satisfaction with outcomes<br>● Treatment effectiveness<br>Satisfaction with service<br>● Staff attitudes<br>● Hospital capacity and surgery<br>● Supplies availability<br>● Waiting time<br>● Hospital efficiency<br>● Care consistent with local beliefs<br>● Timely care<br>● Visitation rules affecting social support
IDENTIFYING AND REACHING CARE	**Actual accessibility of care**<br>Distribution of health facilities [31,37,39]<br>EMS accessibility and timeliness of response [2,37]<br>Travel time [1,40,4,14]<br>Prehospital care [33,41,42,37,39]<br>Costs

	EMS transport protocols<br>● Closest hospital versus most equipped hospital [43]
RECEIVING ADEQUATE AND APPROPRIATE TREATMENT	**Actual quality of care**<br>Poorly staffed facilities [33]<br>● Number of staff/lack of personnel<br>● Competence of staff—training of personnel [40,20]<br>● Maldistribution of staff<br>● Lack of managers<br>Poorly equipped facilities [33,39]<br>● Lack of a blood bank<br>● Lack of a functioning operating room (OR)<br>● Lack of medications or supplies<br>● Lack of infrastructure<br>Inadequate management [33,2,4,20,21,31,42,43,22,37,39,27]<br>● Inadequate or delayed diagnosis<br>● Inadequate or delayed treatment<br>● Inappropriate or delayed inter-hospital transfer<br>● Functioning trauma system<br>● Ward care after ER treatment

*Model based on that created for obstetric emergencies (Thaddeus and Maine, 1994). Delay factors examined in other studies, with respect to trauma, are cited here.
**EMS refers not only to the mode of transportation, but also to the care provided while in transport. As perceptions of EMS can affect decisions to seek care, EMS appears in both seeking care and reaching care categories.

Appendix 3. References for articles in literature review.

1. MacLeod JBA, Cohn SM, Johnson EW et al. Trauma deaths in the first hour: are they all unsalvageable injuries? *Am J Surg* 2007;193:195–9. doi: 10.1016/j.amjsurg.2006.09.010.
2. Kleber C, Giesecke MT, Tsokos M et al. Trauma-related preventable deaths in Berlin 2010: Need to change prehospital management strategies and trauma management education. *World J Surg* 2013;37: 1154–61. doi: 10.1007/s00268-013-1964-2.
3. Ray JJ, Meizoso JP, Satahoo SS et al. Potentially preventable prehospital deaths from motor vehicle collisions. *Traffic Inj Prev* 2016;17:676–80. doi: 10.1080/15389588.2016.1149580.
4. Papadopoulos IN, Bukis D, Karalas E et al. Preventable prehospital trauma deaths in a Hellenic urban health region: an audit of prehospital trauma care. *J Trauma* 1996;41:864–9.
5. Schoeneberg C, Schilling M, Probst T et al. Preventable and potentially preventable deaths in severely injured elderly patients: A single-center retrospective data analysis of a german trauma center. *World J Surg* 2014;38:3125–32. doi: 10.1007/s00268-014- 2755-0.
6. Boman H, Bjornstig U, Hedelin A et al. Avoidable deaths in two areas of Sweden--analysis of deaths in hospital after injury. *European Journal of Surgery* 1999;165:828-33
7. Pellicane J, Byrnne K, DeMaria E. Preventable complications and death from multiple organ failure among geriatric trauma victims. *J Trauma* 1992;33:440-44
8. Oliver GJ, Walter DP, Redmond AD. Prehospital deaths from trauma: are injuries survivable and do bystanders help? *Injury* 2017;48:985-91
9. Hussain LM, Redmond AD. Are pre-hospital deaths from accidental injury preventable? *Br Med J (Clin Res Ed)* 1994;308:1077–80. doi: 10.1136/bmj.309.6946.57.
10. Diamond I, Parkin P, Wales P et al. Preventable pediatric deaths in Ontario: a comparative population-based study. *J Trauma* 2009;66:1189-94.
11. Lerer L, Knottebnelt J. Preventable mortality following sharp penetrating chest trauma. *J Trauma* 1994;37:9-12
12. Oliver GJ, Walter DP, Redmond AD. Are prehospital deaths from trauma and accidental injury preventable? A direct historical comparison to assess what has changed in two decades. *Injury* 2017;48:978-84
13. McDermott F, Cordner S, Tremayne A. Evaluation of the medical management and preventability of death in 137 road traffic fatalities in Victoria, Australia: an overview. Consultative Committee on Road Traffic Fatalities in Victoria. *J Trauma* 1996;40:520-33.
14. Esposito T, Sanddal N, Hansen J et al. Analysis of preventable trauma deaths and inappropriate trauma care in a rural state. *J Trauma* 1995;39:955-62.
15. Esposito T, Sanddal N, Dean J et al. Analysis of preventable pediatric trauma deaths and inappropriate trauma care in Montanna. *J Trauma* 1999;47:243-51.

16. Esposito T, Sanddal T, Reynolds S et al Effect of a voluntary trauma system on preventable death and inappropriate care in a rural state. *J Trauma* 2003;54:663-70.
17. Henriksson E, Ostrom M, Eriksson A. Preventability of vehicle-related fatalities. *Accid Anal Prev* 2001;33:467-75.
18. Rautji R, Bhardwaj D, Dogra T. The Abbreviated Injury Scale and its correlation with preventable traumatic accidental deaths: A study from South Delhi. *Medicine Science and the Law* 2006;46:157-65
19. Chiara O, Scott J, Cimbanassi S et al. Trauma deaths in an Italian urban area: An audit of prehospital and in-hospital trauma care. *Injury* 2002;33:553-62
20. Vioque SM, Kim PK, McMaster J. Classifying errors in preventable and potentially preventable trauma deaths: A 9-year review using the Joint Commission's standardized methodology. *American Journal of Surgery* 2014;208:187–94. doi: 10.1016/j.amjsurg.2014.02.006.
21. Montmany S, Pallisera A, Rebasa P et al. Preventable deaths and potentially preventable deaths. What are our errors? *Injury* 2016;47:669-73
22. Saltzherr T, Wendt K, Nieboer P et al. Preventabilityof trauma deaths in a Dutch level-1 trauma centre. *Injury* 2011;42:870-73.
23. Motomura T, Mashiko K, Matsumoto H et al. Preventable trauma deaths after traffic accidents in Chiba Prefecture, Japan, 2011: Problems and Solutions. *Journal of Nippon Medical School* 2014;81:320-27
24. Sampalis J, Boukas S, Nikolis A et al. Preventable death classification: Inter-rater reliability and comparison with ISS-based survival probability estimates. *Accident Analysis and Prevention* 1995;27:199-206.
25. Schoeneberg C, Schilling M, Hussmann B et al. Preventable and potentially preventable deaths in severely injured patients: a retrospective analysis including patterns of errors. *Eur J Trauma Emerg Surg* 2016;1-9
26. Moon S, Lee SH, Ryoo HW et al. Preventable trauma death rate in Daegu, South Korea. *Clini Exp Emerg Med* 2015;2:236–43. doi: 10.15441/ceem.15.085.
27. Ha M, Kim B, Choi S et al. Preventable and potentially preventable traumatic death rates in a neurosurgery department: a single centre experience. *Korean J Neurotrauma* 2016;12:67-71
28. Kuo L, Kaufman E, Hoffman R et al. Failure to rescue after injury is associated with preventability: the results of mortality panel review of failure to rescue cases in trauma. *Surgery* 2017;161:782-90.
29. 65. Kim Y, Jung K. Utility of the international classification of diseases injury severity score: detecting preventable deaths and comparing the performance of emergency medical centres. *J Trauma* 2003;54:775-80.
30. Iau PTC, Ong CL, Chan STF. Preventable trauma deaths in Singapore. *Aust N Z J Surg* 1998;68:820–5. doi: 10.1046/j.1440-1622.1998.01461.x.
31. Sugrue M, Caldwell E, D'Amours S et al. Time for a change in injury and trauma care delivery: A trauma death review analysis. *Aust N Z J Surg* 2008;78:949–54. doi: 10.1111/j.1445-2197.2008.04711.x.

32. Sanddal T, Esposito T, Whitney J et al. Analysis of preventable trauma deaths and opportunities for trauma care improvement in Utah. *J Trauma* 2011;70:970-77
33. Yeboah D, Mock C, Karikari P et al. Minimizing preventable trauma deaths in a limited-resource setting: A test-case of a multidisciplinary panel review approach at the Komfo Anokye Teaching Hospital in Ghana. *World J Surg* 2014;38:1707-12. doi: 10.1007/s00268-014-2452-z.
34. Zafarghandi MR, Modaghegh MHS, Boudsari BS. Preventable Trauma Death in Tehran: An Estimate of Trauma Care Quality in Teaching Hospitals. *J Trauma* 2003;55:459–65. doi: 10.1097/01.TA.0000027132.39340.FE.
35. Maxson T, Mabry C, Sutherland M. Does the institution of a statewide trauma system reduce preventable mortality and yield a positive return on investmnt for taxpayers. *J Am Coll Surg* 2017;224:489-99
36. Navarro S, Montmany S, Rebasa P. Impact of ATLS training on preventable and potentially preventable deaths. *World J Surg* 2014;38:2273–8. doi: 10.1007/s00268-014-2587-y.
37. Lauriola P, Tosatti F, Schiavi A et al. Confidential enquiry into avoidable vehicle accidental deaths in the province of Modena, Italy. *Eur J Epidemiol*, 2000;16:67-74.
38. Stocchetti N. Risk prevention, avoidable deaths and mortality-morbidity reduction in head injury. *Eur J Emerg Med* 2001;8:215-219.
39. Simons R, Brasher P, Taulu T et al. A population-based analysis of injury-related deaths and access to trauma care in rural-remote Northwest British Columbia. *J Trauma* 2010;69:11-9.
40. Chiara O, Cimbanassi S, Pitidis A et al. Preventable trauma deaths: from panel review to population based-studies. *World J Emerg Surg* 2006;1:1–7. doi: 10.1186/1749-7922-1-12.
41. Henry JA, Reingold AL. Prehospital trauma systems reduce mortality in developing countries. *J Trauma Acute Care Surg* 2012;73:261–8. doi: 10.1097/TA.0b013e31824bde1e.
42. Goosen J, Bowley DM, Degiannis E et al. Trauma care systems in South Africa. *Injury*, 2003;34:704–8. doi: 10.1016/S0020-1383(03)00153-0.
43. McSwain N, Rotondo M, Meade et al. A model for rural trauma care. *Br J Surg* 2012;99:309–14. doi: 10.1002/bjs.7734.

Appendix 4. Distribution of cause of death categories for EIDs, trauma, avoidable EIDs and avoidable trauma deaths, 2012-2015.

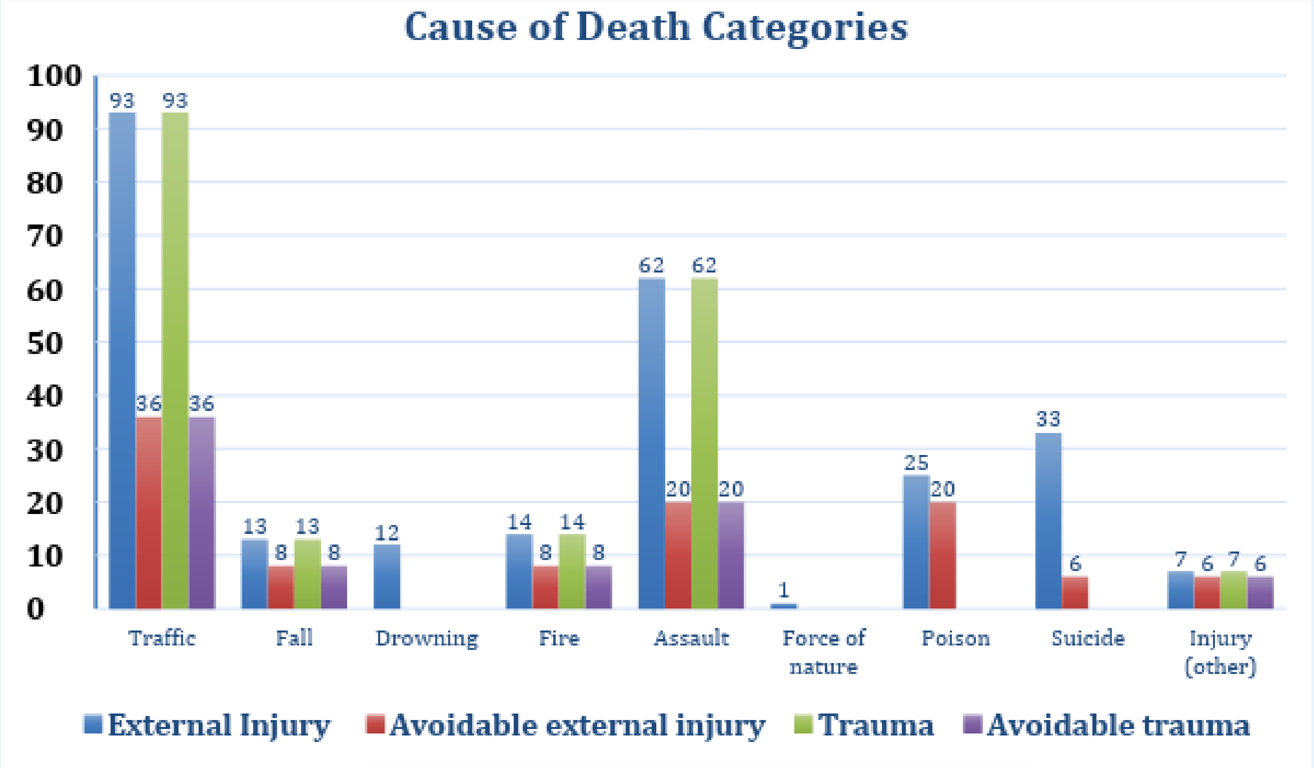

Injury (other) category reflects injuries from objects hitting patients e.g. a football.

Appendix 5. Details of the circumstances of mortality module for the avoidable EIDs (n=109) and trauma (n=81), 2012-2015

Social module category	Indicator	EIDs n (%)	Trauma n (%)
Recognition of severity	Doubts about the need for care	0 (0)	0(0)
	Use of traditional medicine	1(1.0)	1 (1.3)
Mobilising assets to seek care	Did not use a mobile phone to call for help	27 (26)	21 (27)
	Did not use motorised transport to get to care	24 (23)	18 (23)
Access to care	Prohibitive costs	21 (20)	16 (21)
	Did not travel to a hospital or health facility	29 (28)	21 (27)
	>2 hrs travel to care	0 (0)	0 (0)
Quality of care	Problems with admission	2 (1.9)	2 (2.6)
	Problems with treatment (medical treatment, procedures, interpersonal attitudes, respect, dignity)	9 (8.6)	8 (10.2)
	Problems with tests and medications	6 (7.5)	6 (10.2)

Note, for the quality of care indicators and "did not use motorized transport to get to care", n=80 for avoidable EIDs and n=59 for avoidable trauma deaths. This reflects the fact that the denominator in these cases is the total number of eopoe who traveled to hospital

Appendix 6. Details of the key informant interview results.

Seeking care

Health or healthcare literacy

For the general population, HCPs felt that most patients were unaware of proper channels for seeking care, especially when an injury was not acutely severe.

> *"So, there are those who say straight to the hospital and those who say no but you can never actually tell which one because they don't know themselves…it's just a matter of their specific attitude, and there are mistakes in both ways …"*—Medical officer

EMS vs. personal transport

HCPs know that patients are aware of delayed responses from EMS services, so most patients decide between waiting for EMS and seeking personal transport. Previous experience with EMS delays may affect the decision to seek care, as some patients will decide to seek care only when personal transport can be arranged. For those who can afford private ambulance services, this is an easier decision to make, as the private ambulances arrive earlier and are better equipped.

> *"From what we've heard, it's delayed response from the ambulances, the emergency services…the emergency service…it's a problem… and also for the patients, I think because this place is surrounded by villages…if they don't have transport, their own transport to come here, it becomes a bit of a challenge"* –Clinical associate

Mistrust of clinics

Although there may be clinics available, they are not open outside business hours and not well equipped, so patients often decide to wait in the Casualty department of district hospitals, even when their conditions may have been treated in clinics. Although seeking any kind of care after an injury is better than seeking no care at all, for most injuries, seeking care at a hospital may be more appropriate than seeking care in a clinic.

> *"…the patients …they come with their referral, but you will find that most of them don't have a referral, which means that they don't trust their clinic, the clinic for them. Even those cases that can be seen there, but most of the time, they are not seen there."* –Medical officer

> *"They don't really like going to the clinics, because to them, they feel like, they're going to send me to the hospital anyways, so they prefer coming straight to the hospitals, because they feel like they're wasting time."* –Medical officer

Reaching care

EMS accessibility and timeliness

The HCPs felt that the largest barrier for patients reaching care was EMS accessibility. Given the rural area and the limited number of ambulances, patients often wait a long time to be taken to hospital.

"… the biggest problem I think they have is access to emergency care personnel, the paramedics, the ambulance. People may have trauma cases and when they call, the ambulance is already out, attending to trauma care somewhere else. It might take two to three hours for this ambulance to come and pick this patient. Now that is just too long for some cases. That might mean the difference between being normal again and being invalid or even dead. So, that is it. It is just the availability of the service…it's not optimal."—Medical officer

Prehospital care
As well as the issues with EMS timeliness, the care provided by EMS providers is limited by the training they receive, as well as the lack of equipment to provide early resuscitation.

"It's not only death, we can also prevent the sequelae people come in with…they get brain damage, because the care was too late. The thing of the transport is very important, because the other guys, the EMS, if something happens, they are not well trained to resuscitate those patients. I've seen people die here because of that." – Medical officer

<u>Receiving care</u>
Poorly staffed facilities
In the district hospitals, there is a shortage of staff to provide care. The Casualty departments are largely run by clinical associates or junior doctors, who sometimes have difficulty getting senior supervision. Also, in the regional hospital, there is one orthopaedic surgeon and one general surgeon. In the tertiary hospital, there are more orthopaedic and general surgeons but only one neurosurgeon. When these experts are not available, care for the patients is severely delayed.

"From my side, as a clinical associate, some of the patients, you need a second opinion from a doctor or something like that, but it is almost impossible, if not impossible, to get that person whom you can talk to about the patient. So, that's the biggest challenge."—Clinical associate

Inadequately trained staff
An important recurring theme was the need for more training. Many HCPs did not receive standardized training in trauma care e.g. ATLS courses and those that did expressed the lack of continued learning. Not only are there barriers of poor staffing that limit the ability to explore training options located in other provinces, but there are also financial barriers, as HCPs said they financed the course and travel costs independently.

"There is no specific ATLS course given to us, actually in the province as a whole. I understand that in Guateng, and in Limpopo maybe. In other hospitals, they send their doctors, especially their junior doctors, to other places, to do their ATLS training, but with us, there is not that. So, you have to be organized and find out when an ATLS course is going to be, find out for yourself and pay for yourself. Which is not convenient also for the hospital … you cannot go away for a week … how many of us… let's say it's me and my colleague who decide to go. That means for that week, we will leave a hole

in the hospital, leave people struggling here, while you are out to educate yourself. Yes, so it is not the best."—Medical officer

"…now after you've stabilized your patient and there is another trauma case, you have to take your team with you, but someone has to stay and take care of the first patient and now you're faced with a situation where you have to give the most junior person the responsibility of watching the vitals, but they don't really know what they are watching and the next thing you know, the machine alarms go off … they do not know what they are watching… they do not know that the [oxygen] saturation is not supposed to drop below 85% and then eventually you find yourself certifying a patient who … (heavy sigh)… you see what I mean?"—Medical officer

Lack of equipment and supplies
Given financial constraints, there is often a lack of important medical equipment and supplies. When they are present, they easily become non-functional and are not repaired or replaced on time.

"…the second problem is life support equipment. Today it is there, tomorrow, it is broken down, yes …there is a blood corner, where we keep emergency blood, maybe two or three units in a fridge."—Medical officer

Difficult referral system
A major problem in the care of severely injured patients, who are most commonly neurotrauma patients, is the ability to transfer them to a tertiary care institution, which is the only location for a computed tomography (CT) scan and access to a neurosurgeon. Some of the barriers include miscommunication about which hospitals receive referrals from the district hospitals and limited number of ambulances, especially ICU ambulances.

"the problem with serious trauma is the referral. When you have to refer to a higher institution, it's a hassle, it's a problem"—Clinical associate

"Ok, uhh, the first, the big barrier, is the referral system. I can have a patient here, head injury, low GCS, they bring the patient in respiratory distress. I'll try to stabilize the patient. I'll try to stop the bleeding, stabilize the patient, tube the patient, and then…I'll get the doctors at the higher level, they will accept the patient, but to transfer that patient, I need an ICU ambulance but in the province, there are only two ICU ambulances. So, the ICU ambulance probably has to leave Witbank to come here, maybe it can take three to four hours. Then take the patient from here to Nelspruit, that is six hours, then it is a problem…especially since we don't have good ventilation equipment."—Medical officer

"Well, sometimes, it's difficult because our referral system is contradictory. Like last week, we were surprised that our referrals should be going to Themba hospital. Meanwhile, we have been arranging them with Mapulaneng …They said that memo had been written in March, but we didn't know about it. Now, when we communicate with Themba, like last night, the doctor on call was trying to arrange with Themba for

an acute abdomen…but they wanted him to send to Mapulaneng…it's still a bit of a confusion. It's difficult."—Medical officer

Staff morale

Given the numerous challenges described above, the HCPs described feelings of frustration and disappointment with the amount of care they could provide, especially knowing that in better circumstances, they could save more lives.

"If you get an ICU ambulance, you have to get the intermediate trained ICU paramedics, or you have to get a chopper and that takes long so honestly, there is only so much you can do here. Because after you've given your drugs and you've intubated and ventilated, what more can you do? You're just sitting there and the CSF is just leaking and then 6 hours later, they come and you know, come on, you think, why are we even doing this? Then they go there, they do the CT scan and then they find that there is no neurosurgeon on call, so what do they do now? They just send the patient back. So, in terms of trauma, we still have a long way to go."—Medical officer
