# Supplementary material for: External injuries, trauma and avoidable deaths in Agincourt, South Africa: a retrospective observational and qualitative study
Source: BMJ Open. 2019 Jun 4;9(6):e027576. doi: 10.1136/bmjopen-2018-027576 (PMC6561452; doi:10.1136/bmjopen-2018-027576)
Supplement: Author's manuscript [file bmjopen-2018-027576.draft_revisions.pdf]

BMJ Open is committed to open peer review. As part of this commitment we make the peer review history of every article we publish publicly available.

When an article is published we post the peer reviewers' comments and the authors' responses online. We also post the versions of the paper that were used during peer review. These are the versions that the peer review comments apply to.

The versions of the paper that follow are the versions that were submitted during the peer review process. They are not the versions of record or the final published versions. They should not be cited or distributed as the published version of this manuscript.

BMJ Open is an open access journal and the full, final, typeset and author-corrected version of record of the manuscript is available on our site with no access controls, subscription charges or pay-per-view fees (http://bmjopen.bmj.com).

If you have any questions on BMJ Open's open peer review process please email info.bmjopen@bmj.com

BMJ Open

EXTERNAL INJURIES, TRAUMA AND AVOIDABLE DEATHS IN AGINCOURT, SOUTH AFRICA

Journal:	*BMJ Open*
Manuscript ID	bmjopen-2018-027576
Article Type:	Research
Date Submitted by the Author:	04-Nov-2018
Complete List of Authors:	EDEM, IDARA; UNIVERISITY OF OTTAWA, NEUROSURGERY<br>Dare, Anna; University of Toronto Faculty of Medicine, Department of Surgery<br>Byass, Peter; Umea Universitet, Umeå Centre for Global Health Research<br>D'Ambruoso, L; University of Aberdeen School of Medicine and Dentistry, Centre for Global Development and Institute of Applied Health Sciences<br>Kahn, Kathleen; University of the Witwatersrand School of Public Health<br>Leather, Andy; King's Health Partners and King's College London, King's Centre for Global Health<br>Tollman, Stephen; University of the Witwatersrand School of Public Health<br>Whitaker , John; King's Health Partners, King's Centre for Global Health<br>Davies, Justine ; King's College London, Centre for Global Health
Keywords:	ACCIDENT & EMERGENCY MEDICINE, EPIDEMIOLOGY, Quality in health care < HEALTH SERVICES ADMINISTRATION & MANAGEMENT, ORTHOPAEDIC & TRAUMA SURGERY, PUBLIC HEALTH

SCHOLARONE™
Manuscripts

External injuries, trauma and avoidable deaths in Agincourt, South Africa

Idara Edem – 1; Anna Dare – 2; Peter Byass – 3, 4; Lucia D'Ambruoso – 3, 5; Kathleen Kahn – 3; Andy Leather – 6; Stephen Tollman – 3; John Whitaker -6; Justine Davies – 7, 3.

Authors are in alphabetical order, apart from first, second, and last authors.

1. Division of Neurosurgery, Department of Surgery, University of Ottawa, Ottawa, Ontario, Canada
2. Centre for Global Health Research, St. Michael's Hospital and University of Toronto, Toronto, Ontario, Canada
3.MRC/Wits Rural Public Health and Health Transitions Research Unit—School of Public Health, Faculty of Health Sciences, University of the Witwatersrand, Johannesburg/Acornhoek, South Africa
4. WHO Collaborating Centre for Verbal Autopsy, Epidemiology & Global Health, Dept. of Public Health & Clinical Medicine, Umeå University, Umeå, Sweden
5. Centre for Global Development, University of Aberdeen, Aberdeen, Scotland
6. King's Centre for Global Health, King's College London, Denmark Hill, London, UK
7. Institute for Applied Health Research, University of Birmingham, Birmingham, UK

Corresponding Author
Idara Edem
725 Parkdale Avenue, WM 150F, Mailbox 411
Ottawa, Ontario, Canada
K1Y4E9
iedem@toh.ca
613-282-8690

For peer review only

ABSTRACT

Objective: Injury burden is highest in Low and Middle Income Countries (LMICs). To reduce avoidable deaths, it is necessary to identify health system deficiencies that prevent timely, quality care. We developed criteria to use Verbal Autopsy (VA) data to identify avoidable deaths and associated health system deficiencies.

Setting: Agincourt, rural Bushbuckridge municipality, Mpumalanga province, South Africa.

Participants: Agincourt Health and Socio-Demographic Surveillance System (HDSS) and 7 Health Care Providers (HCPs) from local hospitals.

Methods: Retrospective study using VA data (2012-2015). Trauma deaths were a subset of External Injury Deaths (EIDs). Criteria to identify avoidable deaths and a three-delays framework applicable for use with VA data was constructed based on literature review and descriptive analysis of data from 2015. This definition was then applied to capture the burden of avoidable deaths and the implicated delays to care. The findings were validated by external expert review. Key informant interviews (KIIs) with HCPs were performed to further explore delays to care.

Results: Avoidable death was defined with a focus on injury survivability, using level of consciousness at the scene and ability to seek care post-injury as indicators. Of 260 EIDs (189 trauma deaths) from 2012-2015, there were 104 (40%) avoidable EIDs and 78 (30%) avoidable trauma deaths (41% of trauma deaths). Delay in receiving care was the largest contributor to avoidable EIDs (61%) and trauma deaths (59%) followed by delay in seeking care (24% and 23%) and in reaching care (15% and 18%). KIIs revealed context-specific factors contributing to the third delay, including difficult referral systems, and inadequately trained staff.

Conclusions: Forty-one percent of trauma deaths in this cohort were avoidable, mainly occurring due to facility-based delays in care. Interventions, including strengthening referral networks, may substantially reduce trauma deaths. These methods could be expanded to other contexts with VAs, to develop context-appropriate interventions.

Key words: Trauma, Avoidable death, Verbal autopsy, Rural, South Africa

For peer review only

ARTICLE SUMMARY: STRENGTHS AND LIMITATIONS OF THIS STUDY

- In this study, we have used Verbal Autopsy (VA) reports to ascertain avoidable mortality and access to care in trauma. As far as we are aware, this is the first time that verbal autopsy data has been used in this manner.

- As well, it is the first time that the three delays framework for access to emergency medical care has been systematically applied to trauma and avoidable mortality, allowing for the quantification of the delays to trauma care.

- Although this study was performed in a single Health and Socio-Demographic Surveillance System (HDSS), the findings are novel, pertinent to the Sustainable Development Goals, and should be of interest to readers, especially as the methodology can be more widely applied to other such HDSSs worldwide.

- VA data does not capture detailed vitals at the scene or the nature of pre-hospital and in-hospital emergency care received. Also, key informant interviews of health care workers added useful information to the VA data, but this information is likely to be skewed towards healthcare facilities.

- The VA methodology involves interviews conducted by well-trained but non-medical personnel, which can result in missing important clinical aspects of the injury and the care sought. Also, since the VA interviews are conducted months after the death of the individual, recall bias becomes a factor, affecting the reliability and validity of the information provided.

For peer review only

INTRODUCTION

Injuries account for an estimated 5.8 million deaths per year, 32% more than HIV, malaria and TB, combined.[1] The burden of trauma deaths is largest in Low and Middle Income Countries (LMICs) which are also the most ill-equipped to manage this burden.[2] Although there has been a global drive for injury prevention and we are in the "decade of action for road safety", morbidity and mortality from injuries continues to rise.[3] Alongside injury prevention efforts, optimization of medical care provided after injury occurrence is important. In many LMICs, there are deficiencies in Emergency Medical Services (EMS) and definitive hospital care for the injured. Where present, these are hampered by limited geographical coverage, resources, and trained staff.[4]

In order to improve a health system's ability to manage a large burden of injuries, gaps in care provision must be identified to guide resource allocation. To assist with this, the concept of avoidable (preventable) deaths—defined as deaths that are completely or partially preventable, given the provision of effective health care—can be used[5] in association with exploration of the factors which led to an avoidable death occuring.

The three-delays framework (delays in seeking care, reaching care and receiving care) for accessing safe, affordable, and timely medical care is regarded as a classic conceptualization of delayed care in emergency situations, reflected in its application in obstetric emergencies[6], sepsis management,[7] perinatal care,[8] and hip fractures.[9] To the best of our knowledge, the three-delays model has not previously been analytically applied to trauma, but given the similarities between trauma and other time-critical conditions in which it has been used,[6] it is an appropriate model for investigating gaps in the health system contributing to avoidable trauma deaths.

To explore avoidable trauma deaths, a complete understanding of deaths and the events surrounding these deaths is needed. A lack of Civil Registration and Vital Statistics (CRVS) systems in many LMICs,[10] coupled with limited physician-assigned cause of death documentation, means that the verbal autopsy (VA) has become a practical tool for ascertaining cause of death, including for injuries.[11] VAs have not previously been used to examine avoidable deaths in trauma. Our aims were: 1) To develop and validate criteria applicable to VA data to estimate numbers of avoidable deaths in trauma and other external injures, and, in cases where deaths were avoidable, determine the barriers to access of care using a three delays framework; 2) To combine information from the literature, VA, and qualitative analysis to construct a three delay's framework applicable to avoidable mortality in trauma.

METHODS

Setting

The study was performed in Agincourt, located in the rural Bushbuckridge municipality of Mpumalanga province, South Africa. A Health and Socio-Demographic Surveillance System

For peer review only

(HDSS) has regularly collected household data on health and vital events (births, deaths, migrations) for over 115,000 people in a geographically defined area since 1992. All deaths identified during surveys are followed up and a VA is conducted within one year of the death.

Literature review

To determine definitions and criteria for avoidable mortality and factors contributing to the three delays in trauma and external injuries, PubMed, Ovid and EMBASE were searched using the terms: "trauma" AND "avoidable death" OR "preventable death" OR "mortality" OR "delays" and limited to papers published in English, from 1990 onwards, in HICs and LMICs. The titles and abstracts were reviewed to select papers addressing avoidable death in trauma and a discussion of delays to care.

Verbal Autopsy

VA involves interview of the primary caregiver of the deceased by a trained fieldworker about signs, symptoms and circumstances surrounding death, using a validated questionnaire[12]. Currently, a computer algorithm, InterVA4, is utilized to determine cause of death, using standardized probabilistic models.[13]

VA data collected using the WHO 2012 short-form VA[14] was utilized. In addition to standard medical questions to allow cause of death to be ascertained, this contains a *trauma module*—41 questions to further query circumstances surrounding trauma deaths—and 10 "circumstances of mortality" (COM) questions that address the "household, community and health systems determinants of health"[15] influencing mortality. In the trauma module, for each category of injury, there are further specific questions to pinpoint the exact details of the deaths. COM questions are grouped under 4 themes—recognition of severity, mobilizing assets to seek care, access to care, and quality of care. Additionally, VAs contain a free text summary of the circumstances surrounding deaths, obtained from the respondent, that allows information not captured in the survey questions to be ascertained.

Data from all VAs between 2012 and 2015 categorized as "external injury deaths" (EIDs)[1] by INTERVA-4 were extracted.[13] Trauma deaths are a subclassification of EIDs, which also include poisoinings and deaths from natural disasters. Additionally, free-text searches of the open free text summary portion of the VAs were performed to identify injury deaths that may have been incorrectly categorized. Search terms included: injury, trauma, fall, accident, and fire.

Defining avoidable death and developing a three-delays framework for application to VA data

Findings from the literature review informed which variables contained in the VA questions and factors extractable from the free text had utility for defining avoidable deaths and in the creation of a three-delays framework. To support the information contained in the VA questions, each narrative in the free text was reviewed for details pertaining to location of injury or death, acuity or chronicity of injury, signs and symptoms after injury, ability to seek

[1] EIDs correspond to external causes of death in chapter 12 of the WHO 2012 VA instrument (WHO, 2012).

For peer review only

care, factors facilitating or inhibiting access to care, the provision of medical care, results of care delivered, and understanding of the effects of care on the patient's outcome.

VA reports on deaths from 2015 were first utilized to identify relevant variables and categorise deaths by avoidability and delay. The choice of variables and categorisations of avoidability and three delays in a subsample of 20 cases were then validated by external review with a researcher and trauma care provider (JW) familiar with the geographic context. Discrepancies were discussed until consensus was achieved in order to improve classification of subsequent data. These criteria for avoidability and delays were then applied to all EIDs from 2012 to 2014. Where there were multiple delays contributing to the avoidable mortality, that which was found to be the most immediate factor was considered the primary contributing delay.

Qualitative analysis

Emergency-care providers were purposively identified to participate in Key Informant Interviews (KIIs) from local hospitals and snowball sampling was used until responses reached saturation.[16] The interviews were face-to-face semi-structured and open-ended. They were completed in English, recorded, transcribed, and transferred for analysis into NVivo version 11.4 (QSR International, 2017). Transcripts were analysed using framework methods [17] within a thematic analysis. This allowed for unanticipated meanings to emerge from the data and analysis, as well as for the data to be structured based on the overall aims and objectives of the study, thus permitting both inductive and deductive data analyses.[18] This was an iterative process, to ensure that the content of the narrative data was as fully represented as possible. Identified factors were then compared with those garnered from the literature review and the VA analysis.

Final three delays framework

The findings from the literature review, VA, and qualitative analyses were combined to construct a holistic three delays framework for assessing avoidable deaths in trauma.

Statistical considerations

This is an exploratory study and a power calculation was not performed. Data are described. Analysis was performed using SPSS Statistics software (IBM, version 24), NVivo version 11.4 (QSR International, 2017) and Microsoft Excel.

Ethics approval and consent

Ethics approval was obtained from the University of the Witwatersrand Human Research Ethics Committee (M170269) and King's College London Research Ethics Committee (LRU-16/17-4313). Participation in the qualitative study was voluntary and informed consent was obtained for the interview and its recording. All interviews were confidential and narratives were anonymized .

RESULTS

Defining avoidable mortality

For peer review only

Literature review

Fifty-seven papers which explored the conceptualisations of avoidable death in trauma and delays in access to care were identified, as were nine additional papers from review of references sections. Despite differences in the definition of avoidable death in trauma, common themes included: injury survivability, deviations from delivery of optimal care and implication of errors in care delivery on the death of the patient. These themes are also reflected in the WHO guideline for the definition of preventable death in trauma—note that this guideline definition also includes calculations of the probability of survival and injury severity scores.[2] Definitions of injury survivability which do not use injury severity scores were based on vital signs at the scene;[19] being awake at the scene;[19-21] or being able to seek care after injury; or having reversible, stable,[22] or non-severe injuries involving a single-system.[23,21,19,20] Most studies used a combination of these definitions (see appendix 1 for details on approaches to determine criteria for injury survivability).

VA Data

Figure 1 shows VA numbers included in the study. Between 2012 and 2015, there were 3001 deaths; of these 260 deaths were categorized as EIDs (8.7% of total) of which 189 were due to trauma (Table 1). The sub-category distribution for both EIDs and trauma deaths are shown in Appendix 2. Traffic and assault injuries were the most common causes of EID and trauma deaths.

Table 1. Demographic characteristics of the EID and trauma groups, for all, avoidable, and non-avoidable deaths from 2012-2015

Category	EIDs	Trauma deaths	Avoidable EID	Non-avoidable EID	Avoidable trauma deaths	Non-avoidable trauma deaths
Total	**260**	**189**	**104**	**156**	**78**	**111**
**Age group**						
Older (>65 y)	19 (7.3%)	12 (6%)	12 (11%)	7 (4.5%)	9 (11%)	3(2.7%)
Mid-age (50-64 y)	27 (10%)	20 (11%)	10 (9.6%)	17 (11%)	8 (10%)	12(11%)
Adult (15-49 y)	188 (72%)	143 (76%)	74 (71%)	114 (73%)	57 (73%)	86 (78%)
Child (5-14 y)	14 (5.4%)	7 (3.7%)	4 (3.8%)	10 (6.4%)	1 (1.3%)	6(5.4%)
Under-5 (1-4 y)	10 (3.8%)	5 (2.6%)	3 (2.9%)	7 (4.5%)	2 (2.6%)	3(2.7%)
Infant (1-11 months.)	1 (0.4%)	1 (0.5%)	0	1 (0.6%)	0	1(0.9%)

For peer review only

Neonate (<28 days)	1 (0.4%)	1 (0.5%)	1 (1.0%)	0	1 (1.3%)	0
**Sex**						
Male	198 (76%)	147 (78%)	75 (72%)	123 (79%)	59 (76%)	88(79%)
Female	62 (24%)	42 (22%)	29 (28%)	33 (21%)	19 (24%)	23(21%)

In 2015, out of 482 total deaths, there were 48 EID cases which were used to develop criteria for determining avoidability. Descriptive information on injury survivability were the only criteria available. Factors indicating survivability were: being alive at the scene, being able to seek help, or non-severe single-system injuries (Table 2). Twenty (42%) EIDs were determined to be avoidable (Table 1). The external reviewer agreed on classification of avoidability of death in 90% of cases.

Table 2. Details on the data sections of VAs used to define injury survivability and determine delays to care

DESCRIPTION	VA SECTIONS CONTAINING RELEVANT DATA	VARIABLES EXTRACTED
Injury survivability	Demographics	Age
	Acuity of death	Acute versus chronic injury/death
	Previous medical history	List of medical conditions
	Signs and symptoms	System-based list of signs and symptoms
	External injury classification	Category of injury
	Trauma module	Details of injury classification and mechanism of injury
	Free-text disease descriptions	Vitals at the scene<br>Injured body system<br>Signs and symptoms at the scene and at hospital presentation<br>Injury severity<br>Actions taken immediately after injury<br>Ability to seek care
First delay	COM questions	Doubts about the need for care<br>Use of traditional medicine

For peer review only

		Did not use a mobile phone to call for help
	Free-text disease descriptions	Healthcare literacy and perceived severity of the injury<br>Perceived costs of seeking care—transport, medical costs<br>Perceived quality of care<br>Previous experiences with medical care
	Trauma module	Traffic accident location i.e. rural versus urban<br>Death at site or time of injury
Second delay	COM questions	Did not use motorised transport to get to care<br>>2 hrs travel to care<br>Prohibitive costs<br>Did not travel to a hospital or health facility
	Free-text disease descriptions	EMS accessibility<br>EMS timeliness of response<br>EMS versus personal transport<br>Prehospital care
Third delay	COM questions	Problems with admission<br>Problems with treatment (medical treatment, procedures, interpersonal attitudes, respect, dignity)<br>Problems with tests and medications
	Free-text disease descriptions	Inadequate and/or delayed diagnosis and/or treatment

For peer review only

		Delayed inter-hospital transfers<br>Poorly staffed and/or equipped facilities
	Treatment and health care utilization	Medical and/or surgical treatment<br>Traditional medicine treatment<br>If medical and traditional medicine treatment sought, which was sought first?<br>Medical aid or employer support for healthcare<br>Medical actions<br>If no medical actions taken, reasons for not taking medical action

Applying the definition of avoidable deaths to the 2012-2015 VA data revealed 104 avoidable EIDs (40%) and 78 avoidable trauma deaths (41%) (Table 1, Appendix 2).

Developing a three-delays framework for avoidable deaths

Literature review

Relevant components of the three delays model identified in the literature were as follows: deciding to seek care (socio-economic and cultural factors, perceived accessibility and perceived quality of care e.g. perceived severity, transportation, costs, EMS transport protocols, reputation of previous experience); identifying and reaching care (actual accessibility factors e.g. EMS accessibility and timeliness of response, prehospital care); receiving adequate and appropriate treatment (actual quality of care factors e.g. poorly staffed facilities and inadequate management) (Appendices 1 and 3)

VA data

The 48 EIDs from 2015 revealed several extractable factors relevant to each of the three delays (Table 2). There was 80% agreement with the external expert for classification of delays contributing to death. Further analysis and discussion of VA free-text data was performed together, until consensus was reached and a three delay's framework using data relevant to VA was constructed. Data relevant to the COM module are contained in appendix 4.

Applying this three delay's framework to deaths between 2012-2015 showed 58% of people with avoidable EIDs experienced one delay, 38% two delays, and 4% three delays. For avoidable trauma deaths, 57% experienced one delay, 38% two delays, and 5% three delays. For all avoidable deaths, perceived injury severity, deficiencies in prehospital care, and inadequate

For peer review only

and/or delayed diagnosis and/or treatments were the primary contributing factors in delays 1, 2 and 3, respectively (Table 3). Delay three was the most commonly occurring contributing delay to avoidable deaths, in 61% and 59% of EIDs and trauma deaths, respectively.

Table 3. Primary contributing factors in each delay category for avoidable EID and trauma deaths, 2012-2015

**Delay**	**Number (%)**	**Primary contributing delay factors**	**Number (%)**
**Avoidable EIDs (n=109)**			
1—Seeking care	25 (24%)	Perceived severity	35 (74%)
		Perceived etiology	6 (12.7%)
2—Reaching care	16 (15%)	Prehospital care	28 (61%)
		EMS timeliness and response	17 (37%)
3—Receiving care	63 (61%)	Inadequate and/or delayed diagnosis and/or treatment	82 (78%)
		Delayed inter-hospital transfer	11 (10%)
**Avoidable trauma deaths (n=81)**			
1—Seeking care	18 (23%)	Perceived severity	23 (77%)
		Delayed discovery	3 (9.6%)
2—Reaching care	14 (18%)	Prehospital care	25 (63%)
		EMS timeliness and response	13 (34%)
3—Receiving care	46 (59%)	Inadequate and/or delayed diagnosis and/or treatment	59 (77%)
		Delayed inter-hospital transfer	9 (11%)

The COM indicators captured minimal data about the quality of care. Also, there were no cases where respondents reported having “greater than 2 hours’ travel to care” or “doubts about the need for care”. However, a mobile phone was not used to call for help in 26% of EID cases and 28% did not travel to a hospital for care. For avoidable trauma deaths, 27% did not use a phone to call for help and 27% did not travel to a hospital for care. Also, issues with the prohibitive costs of seeking care were present in 20% of the avoidable EIDs and 21% of the avoidable trauma deaths.

For peer review only

KIIs and qualitative analysis

Seven KII were needed to reach saturation; these were done with two clinical associates (three-year training for a Bachelor of Clinical Medical Practice), four medical officers (four-year medical degree, two-year internship, one-year community service), and one Casualty (ER) manager working in the three hospitals in Bushbuckridge: Matikwane, Tintswalo and Mapulaneng. Important themes included healthcare literacy, EMS versus personal transport, mistrust of clinics (delay 1); EMS accessibility and timelines, prehospital care (delay 2); poorly staffed facilities, inadequately trained staff,lack of equipment and supplies, difficult referral systems and staff morale (delay 3). See below and Appendix 5 for full details on these themes).[2]

Seeking care

Health or healthcare literacy

KIs felt that most patients were unaware of proper channels for seeking care, especially when an injury was not acutely severe.

> *"So, there are those who say straight to the hospital and those who say no but you can never actually tell which one because they don't know themselves… it's just a matter of their specific attitude, and there are mistakes in both ways …"*—Medical officer

EMS vs. personal transport

KIs recounted that patients are aware of delayed responses from EMS and previous experience with EMS delays may affect the decision to seek care.

> *"From what we've heard, it's delayed response from the ambulances, the emergency services …it's a problem… and also for the patients, I think because this place is surrounded by villages…if they don't have transport, their own transport to come here, it becomes a bit of a challenge"* –Clinical associate

Reaching care

EMS accessibility and timeliness

The KIs felt that the largest barrier for patients reaching care was EMS accessibility. Given the rural area and the limited number of ambulances, several descriptions were provided of patients waiting for long periods to be taken to hospital.

> *"… the biggest problem I think they have is access to emergency care personnel, the paramedics, the ambulance. People may have trauma cases and when they call, the ambulance is already out, attending to trauma care somewhere else. It might take two to three hours for this ambulance to come and pick this patient. Now that is just too long*

[2] Note that an abridged version of the KII data analysis is presented here, but more details are presented in Appendix 5.

For peer review only

for some cases. That might mean the difference between being normal again and being invalid or even dead."—Medical officer

Prehospital care

As well as the issues with EMS timeliness, the care provided by EMS providers was described as limited by the training received, as well as by lack of equipment to provide early resuscitation.

> *"It's not only death, we can also prevent the sequelae people come in with…they get brain damage, because the care was too late. The thing of the transport is very important, because the other guys, the EMS, if something happens, they are not well trained to resuscitate those patients. I've seen people die here because of that."* –Medical officer

Receiving care

Inadequately trained staff

A key recurring theme was the need for more training. Many KIs reported that they did not receive standardized training in trauma care (e.g. Advanced Trauma Life Support [ATLS] courses) and those that did expressed the lack of continued learning.

> *"There is no specific ATLS course given to us, actually in the province as a whole…In other hospitals, they send their doctors, especially their junior doctors, to other places, to do their ATLS training, but with us, there is not that. So, you have to be organized and find out when an ATLS course is going to be, find out for yourself and pay for yourself. Which is not convenient also for the hospital … you cannot go away for a week…let's say it's me and my colleague who decide to go. That means for that week, we will leave a hole in the hospital."*—Medical officer

Lack of equipment and supplies

KIs reported that given financial constraints, there is often a lack of important medical supplies and when they are present, they easily become non-functional without being repaired or replaced on time.

> *"…the second problem is life support equipment. Today it is there, tomorrow, it is broken down, yes …there is a blood corner, where we keep emergency blood, maybe two or three units in a fridge."*—Medical officer

Difficult referral system

A major problem in the care of severely injured patients, who are most commonly neurotrauma patients, is the ability to transfer them to a tertiary care institution, which is the only location for a computed tomography (CT) scan and access to a neurosurgeon. KIs recounted that some of the barriers include miscommunication about where to transfer patients to and limited ICU ambulances.

For peer review only

> *"Ok, the first, the big barrier, is the referral system. I can have a patient here, head injury, low GCS [Glasgow Coma Scale], they bring the patient in respiratory distress. I'll try to stabilize the patient…tube the patient…I'll get the doctors at the higher level, they will accept the patient, but to transfer that patient, I need an ICU ambulance but in the province, there are only two ICU ambulances…maybe it can take three to four hours…Then take the patient from here to [the other hospital], that is six hours, then it is a problem…especially since we don't have good ventilation equipment."*—Medical officer

> *"Well, sometimes, it's difficult because our referral system is contradictory. Like last week, we were surprised that our referrals should be going to [this] hospital. Meanwhile, we have been arranging them with [another hospital] …They said that memo had been written in March, but we didn't know about it."*—Medical officer

Composite three-delays framework for avoidable deaths in trauma

The creation of the final three-delays framework for avoidable death in trauma based on analysis of information from three methodologies (literature review, VA analysis, and KIIs) is shown in Figure 2.

DISCUSSION

Between 2012 to 2015, a large proportion of external injury and trauma deaths were avoidable. Most avoidable EID and trauma deaths occurred in male adults between 15-49 years old and were a result of traffic injury or assault. A delay in receiving definitive care (the 'third delay') was the largest contributing factor in avoidable deaths, and occurred as a result of delays in diagnosis and treatment, inadequate referral systems for injured patients and poorly staffed and equipped health facilities, particularly at the district hospital level. These findings demonstrate that VA data examining the nature of the injury and circumstances around death can be used to determine avoidable injury deaths and explore the factors contributing to these. Qualitative information from local health care providers provided useful additional information.

Globally, there is an increasing prevalence of trauma morbidity and mortality, especially in people younger than 45 years old,[24] with road traffic injuries being the second leading cause of death in 15-49 years old and the first in males.[25-26] In Mpumalanga, road traffic injuries were the second leading cause of years of life lost in 2012, after HIV/AIDS.[27] Trauma burden is worse in LMICs, given the limited scope of trauma systems and the challenges of service delivery in already strained health systems.[4] When injuries do occur, it is important to have trauma systems in place that ensure that the injured person receives care from the time of injury occurrence, to reduce avoidable mortality. The logical way to develop such systems in countries with nascent health care systems is to first identify gaps in current services. To our knowledge, this is the first time that data from VAs has been used to do this. Findings from this study should contribute to future local health system planning and create a path for future studies at other HDSSs collecting VA data.

For peer review only

Avoidable mortality was indentified using data available from VA with criteria informed by a review of the literature. In trauma, there is heterogeneity in methods for categorizing avoidable deaths, but injury survivability—defined using injury severity scores and expert panels—is the commonest methodology.[28] Although injury survivability scores were not available in this case, VA contained data which were used – with good agreement – to define survivability of injury. It is reassuring that identified themes here are also reflected in the WHO guideline for the definition of preventable death in trauma. The results on rates of avoidability are also similar to findings from other LMICs.[4,29-31] However, the avoidable death rate found in this study is greater than that found in high income countries.[28,32-34] The findings that most avoidable deaths were in the group defined by VA as adults (between 15 and 49 years old) and in males are also consistent with previous studies on trauma deaths.[21,35,24]

In this study, delays in receiving care could not be ascertained from VA review alone and KIIs provided useful additional data. HCPs revealed that factors including difficult referral systems, poorly staffed facilities, inadequately trained staff, and lack of equipment and supplies, all contributed to delayed or inadequate diagnosis and/or treatment, and thus avoidable death. These factors have also been shown to be contributors to preventable trauma deaths in other studies[36-39] and they represent possible intervention points. Potentially, the widespread introduction of trauma training, including ATLS and training provided to HCPs in LMICs by institutions like Primary Trauma Care Foundation, could lead to reduced trauma avoidable mortality, as has been seen in other studies.[40] A review of the interventions to improve trauma care quality in LMICs, their cost-effectiveness and possible application in this setting, may be a worthwhile future study to pursue. Although the delays are presented linearly to enable understanding, they are actually interconnected, as delays in reaching and receiving care at one instance will influence the decision to seek care in future instances. Therefore, what is determined to be the most immediate factor contribuiting to death and thus the largest contributing delay, may actually be the outcome of the effect of other delays in the past. As well, patients cycle through the delays model as their care plans change and they require transfer to other facilities, such that these patients face new and compounding delays as their care provision progresses.

The result that a substantial proportion of mortality is avoidable seems at odds with the findings in a recent study showing that, in South Africa, only 5.2% of the population is estimated to be outside a 2-hour travel time to emergency care.[41] These results are reflected in the analysis of the COM data where there were no reports of being outside a 2-hour travel distance to care. Yet, on examination of other parameters, including the free text, delays in reaching care – especially *appropriate* care – did contribute to avoidable mortality. Indeed, avoidable mortality may be best reduced by introducing effective prehospital trauma care[36] and by ensuring that a bypass protocol is implemented,[42] so that patients with severe trauma injuries are transported immediately to secondary or tertiary hospitals without first being delayed in district facilities. Many severely injured patients in this study experienced a substantial delay when sent first to district hospitals before awaiting transfer to regional or tertiary hospitals. The goal of a well-functioning trauma system should be that the “right patient is treated at the right hospital at the right time”.[43] So, if patients are severely injured,

For peer review only

they are taken directly to hospitals with appropriate specialists. This has the advantage of eliminating inter-hospital transfer delays. However, the success of a trauma system with targeted patient transfers also depends on an appropriately equipped and staffed prehospital care system, which is lacking in many LMICs, as shown here.

Neurotrauma was a significant cause of avoidable trauma deaths in this study, as seen both from findings of the KIIs and VA review, where 35% of the avoidable trauma deaths had neurotrauma. This finding is likely an under-estimation, given the lack of detailed medical information. Other studies have also found that most of the avoidable deaths in trauma are due to neurotrauma[44,19], usually related to poor airway management.[45,24] In Bushbuckridge, where access to a neurosurgeon occurs only in a tertiary hospital outside of the municipality, it is paramount to provide adequate prehospital care, including proper airway management, as well as reduce inter-hospital transfer delays.

This study had several limitations. VA data does not capture detailed vitals at the scene, exact pre-hospital delays, or the nature of pre-hospital and in-hospital emergency care received. Furthermore, the COM indicators provided information for the delays, but they did not capture important information about delays in reaching appropriate or quality care. The use of these indicators in South Africa was analysed in the census rounds of 2012-2013 and the researchers found that problems with not calling for help or not going to a medical facility were more pronounced than problems with overall costs in the younger age groups and in those with acute conditions, as in most trauma cases.[15] In this study, KIIs added useful information to the VA data, but this information is likely to be skewed towards healthcare facilities. If other service providers or users were interviewed, further information about delays to access in care which are not contained in VA data may have been gleaned. An additional limitation is that the acute nature of trauma may also bias caregivers to report the care provided in hospital in more detail, as was seen in the free-text, so that if not asked specifically about the pre-hospital delays, these are not recollected or recorded. The VA methodology involves interviews conducted by well-trained but non-medical personnel, which can result in important clinical aspects of the injury and the care sought being missed. Since the VA interviews are conducted months after the death of the individual, recall bias becomes a major factor, affecting the reliability and validity of the information provided. Yet, although the VA is not the ideal tool for information on death and causes of death, it is a pragmatic method that provides useful information and its use continues to widen with greater innovations for its application.[15]

CONCLUSION

In this study, VA was shown to be a feasible method for defining avoidable deaths in trauma and ascertaining which of the three delays contribute to death. Between 2012 and 2015, a large percentage of external injury (40%) or trauma (41%) deaths were avoidable. The third delay in the three-delays model was found to be the largest contributor to avoidable deaths and the qualitative study supported the findings from the VA. To combat the burden of avoidable trauma deaths in LMICs, there is a need for a functioning trauma system, with adequate health systems interventions in trauma prevention, access to medical care, EMS, prehospital care and

For peer review only - http://bmjopen.bmj.com/site/about/guidelines.xhtml

adequately staffed and supplied hospitals. “When it comes to trauma care, where people live ought not to determine if they live”.[46]

FIGURE LEGENDS

Figure 1. Deaths considered in this study (EIDs and trauma), relative to total deaths in the HDSS

Figure 2. Composite three-delays framework for avoidable death in trauma.

*The most frequently cited factors from the VA analysis and KIIs analysis are shaded in blue.

ACKNOWLEDGMENTS

We thank Chodziwadziwa Kabudula (MRC/Wits Rural Public Health and Health Transitions Research Unit—School of Public Health, Faculty of Health Sciences, University of the Witwatersrand, Johannesburg/Acornhoek, South Africa) for his assistance with assembling the Agincourt HDSS dataset for our use.

This work has been previously presented at the following conferences: AANS 2018, New Orleans, USA and CNS Bethune Round Table 2018, Toronto, Canada.

FUNDING

Funding for part of this project (travel scholarship) was provided by the King’s College London, Centre for Global Health. This funding source was not involved in the study design; in the collection, analysis and interpretation of the data; in the writing of the manuscript; and in the decision to submit the paper for publication. Otherwise, this research received no specific grant from an agency of the public, commercial or not-for-profit sectors.

COMPETING INTERESTS

The authors do not report any competing interests or conflicts of interest.

CONTRIBUTIONS

IE helped to develop the idea, did the analysis, drafted and revised the paper; AD helped to develop the idea, and inputted into the paper drafts; PB inputted into the idea and the paper drafts; LD inputted into the idea, supported the analyses, and inputted into the paper drafts; AL, KK, and ST inputted into the idea and the paper drafts; KK set up and is overall responsible for the system of verbal autopsies; ST set up and is overall responsible for the Agincourt HDSS; JW contributed to the analyses and inputted into the paper drafts; JD led the project, developed the idea, supported the analyses, and inputted into the paper drafts; all authors approve the final paper for submission.

DATA SHARING

For peer review only

The data used for the VA analysis is available from the INDEPTH Data Repository platform at: http://www.indepth-ishare.org/index.php/home
The supplementary file (appendices) contains further details on the qualitative interviews. More data is available by emailing: iedem@toh.ca

PATIENT AND PUBLIC INVOLVEMENT STATEMENT

Patients were not directly involved in this study, as the patient information was obtained from the INDEPTH VA data repository. No new patient data was collected.

Before the study began, there were meetings between members of the KCGH team and researchers at the Agincourt site. These meetings helped to clarify and refine the research idea as well as determine the external assistance that would be required to ensure its completion. Assistance from experts at the Agincourt site was sought for local ethics approval, data retrieval and KII recruitment. These responsibilities for the Agincourt site were developed early on, to reflect the expertise and knowledge of the team. Constant communication and feedback with the team allowed for early intervention when problems arose, like the issue of delayed local ethics approval.

Involvement of all stakeholders in the design and implementation of this study occurred in different phases. So, although the focus of the study was on creating tools that are generalizable to other HDSS sites and the focus of the research unit was on consolidating the methodologies for future capacity development, the local Public Health experts help ensure that the results are contextual and reflective of the community needs.

Since it is also important to utilize the most effective communication channels, the platform of the INDEPTH website would be a useful avenue for fellow researchers. The results will be disseminated to those providing healthcare in Bushbuckridge and the Mpumalanga Province in the form of printed reports. Also, a presentation of the findings was given to the staff of the MRC/Wits-Agincourt unit, in the format of a critical review and training seminar. These efforts serve to improve the learning of those involved in the VA process and increase the capacity of local researchers. Dissemination to local community members will also be incorporated into the established public engagement strategies of the MRC/Wits-Agincourt unit.

For peer review only

REFERENCES

1. Lozano R, Naghavi M, Foreman K et al. Global and regional mortality from 235 causes of death for 20 age groups in 1990 and 2010: A systematic analysis for the Global Burden of Disease Study 2010. *Lancet* 2012;380:2095–128 doi: 10.1016/S0140-6736(12)61728-0.
2. World Health Organization. Guidelines for Trauma Quality Improvement Programmes. Edited by C. Mock, C. Juillard, S. Brunage, J. Goosen, and M. Joshipura. Geneva: World Health Organization. 2009 doi: 10.1016/j.puhe.2010.01.011.
3. World Health Organization. *Saving Millions of lives: Decade of action for road safety 2011-2020, WHO publication*. Available at: http://who.int/violence_injury_prevention/publications/road_traffic/saving_millions_lives_en.pdf. Accessed March 2017.
4. Yeboah D, Mock C, Karikari P et al. Minimizing preventable trauma deaths in a limited-resource setting: A test-case of a multidisciplinary panel review approach at the Komfo Anokye Teaching Hospital in Ghana. *World J Surg* 2014;38:1707-12. doi: 10.1007/s00268-014-2452-z.
5. Rutstein D, Berenberg W, Chalmers T et al. Measuring the quality of medical care. A clinical method. *N Engl J Med* 1976;294:582-88.
6. Thaddeus S and Maine D. Too Far To Walk: Maternal Mortality in Context. *Soc Sci Med* 1994;38:1091–110.
7. Papali A, McCurdy M, Calvello E. A "three delays" model for severe sepsis in resource-limited countries. *J Crit Care* 2015;30:e9-14 doi: 10.1016/j.jcrc.2015.04.003.
8. Musafili A, Persson L, Baribwira C. et al. Case review of perinatal deaths at hospitals in Kigali, Rwanda: Perinatal audit with application of a three-delays analysis. *BMC Pregnancy and Childbirth* 2017;17:1–13. doi: 10.1186/s12884-017-1269-9.
9. Tewari A, Rath S, Yadav L et al. Care Seeking Behaviour of Older Adults with Hip Fracture in India: A Qualitative Study. *J Pat Care* 2017;3:128. doi: 10.4172/2573-4598.1000128.
10. Mikkelsen L, Phillips D, AbouZahr C et al. A global assessment of civil registration and vital statistics systems: monitoring data quality and progress. *Lancet* 2015;6736: 60171–4.
11. Byass, P. Minimally Invasive Autopsy: A New Paradigm for Understanding Global Health? *PLoS Medicine* 2016;13: 8–11. doi: 10.1371/journal.pmed.1002173.
12. Kahn K, Collinson M, Gomez-Olive F et al. Profile: Agincourt health and socio-demographic surveillance system. *Int J Epidemiol* 2012;41:988–1001.
13. Murray CJ, Lozano R, Flaxman AD et al. Robust metrics for assessing the performance of different verbal autopsy cause assignment methods in validation studies. *Popul Health Metr* 2011;9:28.
14. World Health Organization. Verbal autopsy standards: The 2012 WHO verbal autopsy instrument. Geneva. Available at: http://www.who.int/healthinfo/statistics/verbalautopsystandards/en/index2.html. Accessed January 2017.
15. D'Ambruoso L, Kahn K, Wager RG et al. Moving from medical to health systems classifications of deaths: extending verbal autopsy to collect information on the

For peer review only

circumstances of mortality. *Glob Health Res Policy*. *Global Health Research and Policy* 2016;1:2. doi: 10.1186/s41256-016-0002-y.
16. Ritchie J, Lewis J. Qualitative research practice: A guide for social science students and researchers. Los Angeles: Sage Publishing Ltd 2014.
17. Ritchie J, Lewis J. Qualitative research practice: a guide for social science students and researchers. London: Sage Publishing Ltd 2003.
18. Gale N, Heath G, Cameron E et al. Using the framework method for the analysis of qualitative data in multi-disciplinary health research. *BMC Medical Research Methodology* 2013;13:117. doi: 10.1186/1471-2288-13-117.
19. MacLeod JBA, Cohn SM, Johnson EW et al. Trauma deaths in the first hour: are they all unsalvageable injuries? *Am J Surg* 2007;193:195–9. doi: 10.1016/j.amjsurg.2006.09.010.
20. Kleber C, Giesecke MT, Tsokos M et al. Trauma-related preventable deaths in Berlin 2010: Need to change prehospital management strategies and trauma management education. *World J Surg* 2013;37: 1154–61. doi: 10.1007/s00268-013-1964-2.
21. Ray JJ, Meizoso JP, Satahoo SS et al. Potentially preventable prehospital deaths from motor vehicle collisions. *Traffic Inj Prev* 2016;17:676–80. doi: 10.1080/15389588.2016.1149580.
22. Schoeneberg C, Schilling M, Probst T et al. Preventable and potentially preventable deaths in severely injured elderly patients: A single-center retrospective data analysis of a german trauma center. *World J Surg* 2014;38:3125–32. doi: 10.1007/s00268-014-2755-0.
23. Papadopoulos IN, Bukis D, Karalas E et al. Preventable prehospital trauma deaths in a Hellenic urban health region: an audit of prehospital trauma care. *J Trauma* 1996;41:864–9.
24. Harris T, Davenport R, Hurst T et al. Improving outcome in severe trauma: trauma systems and initial management--intubation, ventilation and resuscitation. *Postgrad Med Journal*, 2012;88:588–94. doi: 10.1136/postgradmedj-2010-74245.
25. Institute for Health Metrics and Evaluation (IHME). GBD Compare. GBD 2016. https://vizhub.healthdata.org/gbd-compare/. Accessed February 18, 2018.
26. Abajobir AA, Abbafati C, Abbas K et al. Global, regional, and national age-sex specific mortality for 264 causes of death, 1980–2016: a systematic analysis for the Global Burden of Disease Study 2016. *Lancet* 2017;390:1151–210. doi: 10.1016/S0140-6736(17)32152-9.
27. Pillay-van Wyk V, Msemburi W, Laubscher et al. Mortality trends and differentials in South Africa from 1997 to 2012: second National Burden of Disease Study. *Lancet Glob Health* 2016;4:e642–e653. doi: 10.1016/S2214-109X(16)30113-9.
28. Oliver GJ and Walter DP. A Call for Consensus on Methodology and Terminology to Improve Comparability in the Study of Preventable Prehospital Trauma Deaths: A Systematic Literature Review. *Acad Emerg Med* 2016;23:503–10. doi: 10.1111/acem.12932.
29. Zafarghandi MR, Modaghegh MHS, Boudsari BS. Preventable Trauma Death in Tehran: An Estimate of Trauma Care Quality in Teaching Hospitals. *J Trauma* 2003;55:459–65. doi: 10.1097/01.TA.0000027132.39340.FE.
30. Moon S, Lee SH, Ryoo HW et al. Preventable trauma death rate in Daegu, South Korea.

For peer review only

Clini Exp Emerg Med 2015;2:236–43. doi: 10.15441/ceem.15.085.

31. Iau PTC, Ong CL, Chan STF. Preventable trauma deaths in Singapore. *Aust N Z J Surg* 1998;68:820–5. doi: 10.1046/j.1440-1622.1998.01461.x.
32. Chiara O, Cimbanassi S, Pitidis A et al. Preventable trauma deaths: from panel review to population based-studies. *World J Emerg Surg* 2006;1:1–7. doi: 10.1186/1749-7922-1-12.
33. Kwon AM, Garbett NC, Kloecker GH. Pooled preventable death rates in trauma patients: Meta analysis and systematic review since 1990. *Eur J Trauma Emerg Surg* 2014;40:279–85. doi: 10.1007/s00068-013-0364-5.
34. Settervall CHC, Domingues C. de A, Cardoso de Sousa RM et al. Preventable Trauma Deaths. *Rev Saude Publica*, 2012;46:1–8. doi: 10.1001/jama.1985.03360080071032.
35. Castillo-Rodríguez L, Díaz-Jiménez D, Castañeda-Orjuela C. et al. Years of Life Lost (YLL) in Colombia 1998-2011: Overall and avoidable causes of death analysis. *PLoS ONE* 2015;10:1–11. doi: 10.1371/journal.pone.0125456.
36. Henry JA, Reingold AL. Prehospital trauma systems reduce mortality in developing countries. *J Trauma Acute Care Surg* 2012;73:261–8. doi: 10.1097/TA.0b013e31824bde1e.
37. Montmany S, Pallisera A, Rebasa P et al. Preventable deaths and potentially preventable deaths. What are our errors? *Injury* 2016;47:669-73
38. Sugrue M, Caldwell E, D'Amours S et al. Time for a change in injury and trauma care delivery: A trauma death review analysis. *Aust N Z J Surg* 2008;78:949–54. doi: 10.1111/j.1445-2197.2008.04711.x.
39. Vioque SM, Kim PK, McMaster J. Classifying errors in preventable and potentially preventable trauma deaths: A 9-year review using the Joint Commission's standardized methodology. *American Journal of Surgery* 2014;208:187–94. doi: 10.1016/j.amjsurg.2014.02.006.
40. Navarro S, Montmany S, Rebasa P. Impact of ATLS training on preventable and potentially preventable deaths. *World J Surg* 2014;38:2273–8. doi: 10.1007/s00268-014-2587-y.
41. Ouma PO, Maina J, Thuranira PN. Access to emergency hospital care provided by the public sector in sub-Saharan Africa in 2015: a geocoded inventory and spatial analysis. *Lancet Glob Health* 2018;2214–109. doi: 10.1016/S2214-109X(17)30488-6.
42. Goosen J, Bowley DM, Degiannis E et al. Trauma care systems in South Africa. *Injury*, 2003;34:704–8. doi: 10.1016/S0020-1383(03)00153-0.
43. McSwain N, Rotondo M, Meade et al. A model for rural trauma care. *Br J Surg* 2012;99:309–14. doi: 10.1002/bjs.7734.
44. Hussain LM, Redmond AD. Are pre-hospital deaths from accidental injury preventable? *Br Med J (Clin Res Ed)* 1994;308:1077–80. doi: 10.1136/bmj.309.6946.57.
45. London JA, Mock CN, Quansah RE. Priorities for improving hospital-based trauma care in an African city. *J Trauma* 2001;51:747–53. doi: 10.1097/00005373-200110000-00021.
46. Berwick DM, Downey AS, Cornett EA. A National Trauma Care System to Achieve Zero Preventable Deaths After Injury Recommendations From a National Academies of Sciences, Engineering, and Medicine Report. *JAMA* 2016;316:927–28. doi: 10.1001/jama.2016.8524.Conflict.

For peer review only

47. Boman H, Bjornstig U, Hedelin A et al. Avoidable deaths in two areas of Sweden--analysis of deaths in hospital after injury. *European Journal of Surgery* 1999;165:828-33
48. Pellicane J, Byrnne K, DeMaria E. Preventable complications and death from multiple organ failure among geriatric trauma victims. *J Trauma* 1992;33:440-44
49. Oliver GJ, Walter DP, Redmond AD. Prehospital deaths from trauma: are injuries survivable and do bystanders help? *Injury* 2017;48:985-91
50. Diamond I, Parkin P, Wales P et al. Preventable pediatric deaths in Ontario: a comparative population-based study. *J Trauma* 2009;66:1189-94.
51. Lerer L, Knottebnelt J. Preventable mortality following sharp penetrating chest trauma. *J Trauma* 1994;37:9-12
52. Oliver GJ, Walter DP, Redmond AD. Are prehospital deaths from trauma and accidental injury preventable? A direct historical comparison to assess what has changed in two decades. *Injury* 2017;48:978-84
53. McDermott F, Cordner S, Tremayne A. Evaluation of the medical management and preventability of death in 137 road traffic fatalities in Victoria, Australia: an overview. Consultative Committee on Road Traffic Fatalities in Victoria. *J Trauma* 1996;40:520-33.
54. Esposito T, Sanddal N, Hansen J et al. Analysis of preventable trauma deaths and inappropriate trauma care in a rural state. *J Trauma* 1995;39:955-62.
55. Esposito T, Sanddal N, Dean J et al. Analysis of preventable pediatric trauma deaths and inappropriate trauma care in Montanna. *J Trauma* 1999;47:243-51.
56. Esposito T, Sanddal T, Reynolds S et al Effect of a voluntary trauma system on preventable death and inappropriate care in a rural state. *J Trauma* 2003;54:663-70.
57. Henriksson E, Ostrom M, Eriksson A. Preventability of vehicle-related fatalities. *Accid Anal Prev* 2001;33:467-75.
58. Rautji R, Bhardwaj D, Dogra T. The Abbreviated Injury Scale and its correlation with preventable traumatic accidental deaths: A study from South Delhi. *Medicine Science and the Law* 2006;46:157-65
59. Chiara O, Scott J, Cimbanassi S et al. Trauma deaths in an Italian urban area: An audit of prehospital and in-hospital trauma care. *Injury* 2002;33:553-62
60. Saltzherr T, Wendt K, Nieboer P et al. Preventabilityof trauma deaths in a Dutch level-1 trauma centre. *Injury* 2011;42:870-73.
61. Motomura T, Mashiko K, Matsumoto H et al. Preventable trauma deaths after traffic accidents in Chiba Prefecture, Japan, 2011: Problems and Solutions. *Journal of Nippon Medical School* 2014;81:320-27
62. Sampalis J, Boukas S, Nikolis A et al. Preventable death classification: Inter-rater reliability and comparison with ISS-based survival probability estimates. *Accident Analysis and Prevention* 1995;27:199-206.
63. Schoeneberg C, Schilling M, Hussmann B et al. Preventable and potentially preventable deaths in severely injured patients: a retrospective analysis including patterns of errors. *Eur J Trauma Emerg Surg* 2016;1-9
64. Kuo L, Kaufman E, Hoffman R et al. Failure to rescue after injury is associated with preventability: the results of mortality panel review of failure to rescue cases in trauma. *Surgery* 2017;161:782-90.
65. Kim Y, Jung K. Utility of the international classification of diseases injury severity score:

For peer review only

detecting preventable deaths and comparing the performance of emergency medical centres. *J Trauma* 2003;54:775-80.
66. Sanddal T, Esposito T, Whitney J et al. Analysis of preventable trauma deaths and opportunities for trauma care improvement in Utah. *J Trauma* 2011;70:970-77
67. Maxson T, Mabry C, Sutherland M. Does the institution of a statewide trauma system reduce preventable mortality and yield a positive return on investmnt for taxpayers. *J Am Coll Surg* 2017;224:489-99
68. Lauriola P, Tosatti F, Schiavi A et al. Confidential enquiry into avoidable vehicle accidental deaths in the province of Modena, Italy. *Eur J Epidemiol*, 2000;16:67-74.
69. Stocchetti N. Risk prevention, avoidable deaths and mortality-morbidity reduction in head injury. *Eur J Emerg Med* 2001;8:215-219.
70. Simons R, Brasher P, Taulu T et al. A population-based analysis of injury-related deaths and access to trauma care in rural-remote Northwest British Columbia. *J Trauma* 2010;69:11-9.
71. Ha M, Kim B, Choi S et al. Preventable and potentially preventable traumatic death rates in a neurosurgery department: a single centre experience. *Korean J Neurotrauma* 2016;12:67-71

For peer review only

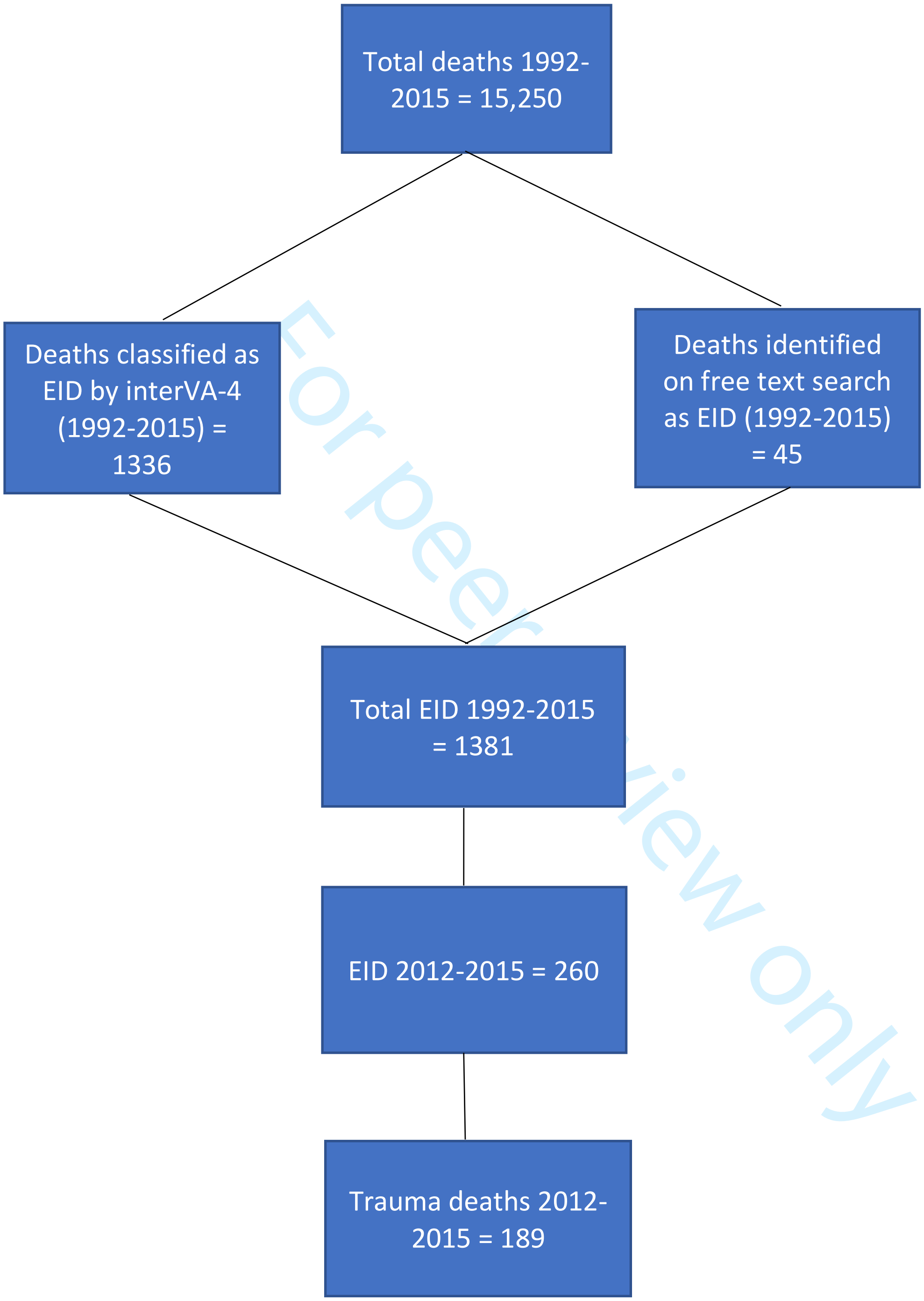

Figure 1. Deaths considered in this study (EIDs and trauma), relative to total deaths in the HDSS

For peer review only

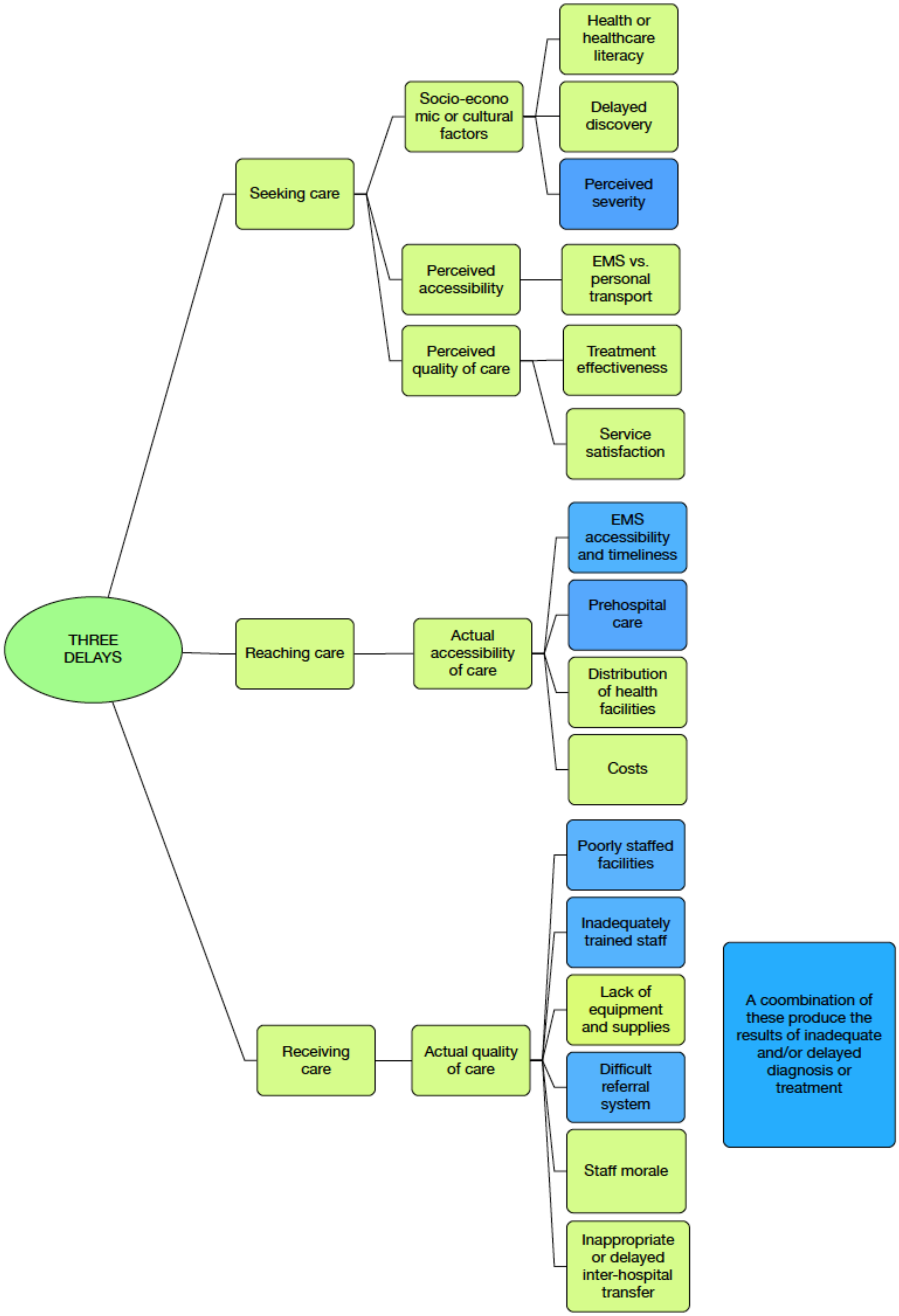

Figure 2. Composite three-delays framework for avoidable death in trauma.

*The most frequently cited factors from the VA analysis and KIIs analysis are shaded in blue.

APPENDICES

Appendix 1 . Details of the definitions of injury survivability

DEFINITIONS OF SURVIVABILITY	STUDIES EXAMINING INJURY SURIVABILITY
Vitals at the scene of injury e.g. palpable pulse or blood pressure	[19]
Awake at the scene	[19-21]
Reversible or treatable injury	[23, 21]
Generally stable at initial treatment or if unstable, becomes stable with initial treatment	[22]
Non-severe injuries	[21]
Non-severe single-system or single-organ or single-vessel injuries	[19-20]
Non-severe non-central nervous system (CNS) injuries	[21]
**APPROACHES TO CRITERIA FOR DEFINITION OF SURVIVABILITY**	**STUDIES**
Abbreviated Injury Scale (AIS)/Injury Severity Scores (ISS)/Probability of Survival (PS)/Trauma Score (TS)	[47-49]
AIS/ISS/PS and specific preventable factors	[44, 50-52]
AIS/ISS/PS and impact of care delivered	[53-59, 39, 37, 60-63, 30, 71, 64-65, 31, 38]
Survivable injuries (subjective/panel)	[22]
Survivable injuries (subjective/panel) and impact of care delivered	[20, 23, 66, 4, 29, 38, 19, 22, 67, 40, 21]
Impact of care delivered	[68-69]

For peer review only

Appendix 2. Distribution of cause of death categories for EIDs, trauma, avoidable EIDs and avoidable trauma deaths, 2012-2015.

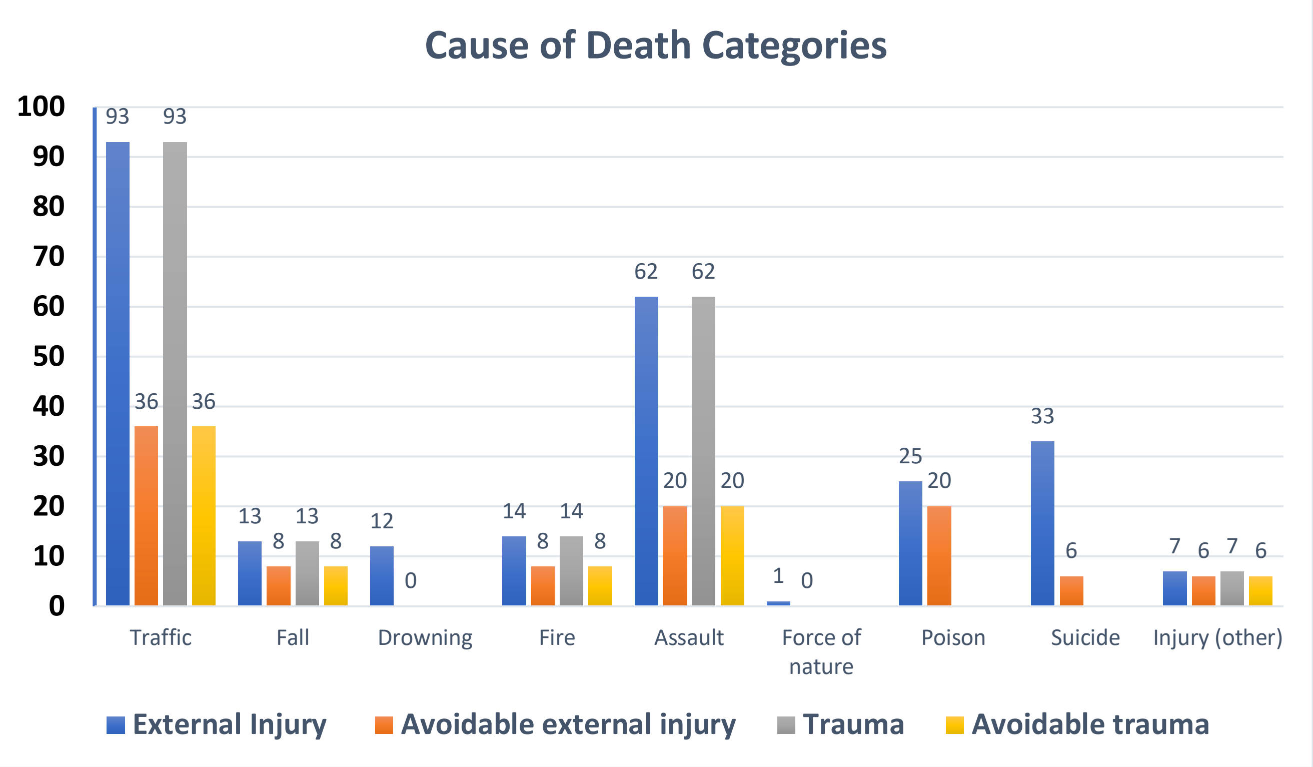

Injury (other) category reflects injuries from objects hitting patients e.g. a football.

view only

Appendix 3. Details of the three-delays model for care extacted from literature review*

DELAY	FACTORS AFFECTING UTILIZATION OR OUTCOME
DECIDING TO SEEK CARE	**Socio-economic/Cultural**<br>Illness factors<br>• Healthcare literacy<br>• Delayed discovery [23,54]<br>• Perceived severity<br>• Perceived etiology<br>Economic status [70]<br>Educational status
	**Perceived Accessibility**<br>Distance<br>Transportation [23]<br>• EMS versus personal transport**<br>• Road conditions<br>• Season—dry or rainy<br>Costs (Simons et al., 2010)<br>• Transportation<br>• Medical care<br>• Medications<br>• Opportunity costs<br>• Lost income<br>EMS transport protocols [70]<br>• Closest hospital versus best equipped hospital
	**Perceived quality of care**<br>Reputation or previous experience<br>Satisfaction with outcomes<br>• Treatment effectiveness<br>Satisfaction with service<br>• Staff attitudes<br>• Hospital capacity and surgery<br>• Supplies availability<br>• Waiting time<br>• Hospital efficiency<br>• Care consistent with local beliefs<br>• Timely care<br>• Visitation rules affecting social support
IDENTIFYING AND REACHING CARE	**Actual accessibility of care**<br>Distribution of health facilities [38,68,70]<br>EMS accessibility and timeliness of response [20,68]<br>Travel time [19,32,23,54]

For peer review only

	Prehospital care [4,36,42,68,70]<br>Costs<br>EMS transport protocols<br>• Closest hospital versus most equipped hospital [71]
RECEIVING ADEQUATE AND APPROPRIATE TREATMENT	**Actual quality of care**<br>Poorly staffed facilities [4]<br>• Number of staff/lack of personnel<br>• Competence of staff—training of personnel [32,39]<br>• Maldistribution of staff<br>• Lack of managers<br>Poorly equipped facilities [4,70]<br>• Lack of a blood bank<br>• Lack of a functioning operating room (OR)<br>• Lack of medications or supplies<br>• Lack of infrastructure<br>Inadequate management [4,20,23,37-39,42,43,60,68,70,71]<br>• Inadequate or delayed diagnosis<br>• Inadequate or delayed treatment<br>• Inappropriate or delayed inter-hospital transfer<br>• Functioning trauma system<br>• Ward care after ER treatment

*Model based on that created for obstetric emergencies (Thaddeus and Maine, 1994). Delay factors examined in other studies, with respect to trauma, are cited here.
**EMS refers not only to the mode of transportation, but also to the care provided while in transport. As perceptions of EMS can affect decisions to seek care, EMS appears in both seeking care and reaching care categories.

For peer review only

Appendix 4. Details of the circumstances of mortality module for the avoidable EIDs (n=109) and trauma (n=81), 2012-2015

**Social module category**	**Indicator**	**EIDs n (%)**	**Trauma n (%)**
Recognition of severity	Doubts about the need for care	0 (0)	0(0)
	Use of traditional medicine	1(1.0)	1 (1.3)
Mobilising assets to seek care	Did not use a mobile phone to call for help	27 (26)	21 (27)
	Did not use motorised transport to get to care	24 (23)	18 (23)
Access to care	Prohibitive costs	21 (20)	16 (21)
	Did not travel to a hospital or health facility	29 (28)	21 (27)
	>2 hrs travel to care	0 (0)	0 (0)
Quality of care	Problems with admission	2 (1.9)	2 (2.6)
	Problems with treatment (medical treatment, procedures, interpersonal attitudes, respect, dignity)	9 (8.6)	8 (10.2)
	Problems with tests and medications	6 (7.5)	6 (10.2)

Note, for the quality of care indicators and "did not use motorized transport to get to care", n=80 for avoidable EIDs and n=59 for avoidable trauma deaths. This reflects the fact that the denominator in these cases is the total number of eopoe who traveled to hospital

For peer review only

Appendix 5. Details of the key informant interview results.

<u>Seeking care</u>

Health or healthcare literacy

For the general population, HCPs felt that most patients were unaware of proper channels for seeking care, especially when an injury was not acutely severe.

> *"So, there are those who say straight to the hospital and those who say no but you can never actually tell which one because they don't know themselves…it's just a matter of their specific attitude, and there are mistakes in both ways …"*—Medical officer

EMS vs. personal transport

HCPs know that patients are aware of delayed responses from EMS services, so most patients decide between waiting for EMS and seeking personal transport. Previous experience with EMS delays may affect the decision to seek care, as some patients will decide to seek care only when personal transport can be arranged. For those who can afford private ambulance services, this is an easier decision to make, as the private ambulances arrive earlier and are better equipped.

> *"From what we've heard, it's delayed response from the ambulances, the emergency services…the emergency service…it's a problem… and also for the patients, I think because this place is surrounded by villages…if they don't have transport, their own transport to come here, it becomes a bit of a challenge"* –Clinical associate

Mistrust of clinics

Although there may be clinics available, they are not open outside business hours and not well equipped, so patients often decide to wait in the Casualty department of district hospitals, even when their conditions may have been treated in clinics. Although seeking any kind of care after an injury is better than seeking no care at all, for most injuries, seeking care at a hospital may be more appropriate than seeking care in a clinic.

> *"…the patients …they come with their referral, but you will find that most of them don't have a referral, which means that they don't trust their clinic, the clinic for them. Even those cases that can be seen there, but most of the time, they are not seen there."* – Medical officer

> *"They don't really like going to the clinics, because to them, they feel like, they're going to send me to the hospital anyways, so they prefer coming straight to the hospitals, because they feel like they're wasting time."* –Medical officer

<u>Reaching care</u>

EMS accessibility and timeliness

The HCPs felt that the largest barrier for patients reaching care was EMS accessibility. Given the rural area and the limited number of ambulances, patients often wait a long time to be taken to hospital.

For peer review only

"… the biggest problem I think they have is access to emergency care personnel, the paramedics, the ambulance. People may have trauma cases and when they call, the ambulance is already out, attending to trauma care somewhere else. It might take two to three hours for this ambulance to come and pick this patient. Now that is just too long for some cases. That might mean the difference between being normal again and being invalid or even dead. So, that is it. It is just the availability of the service…it's not optimal."—Medical officer

Prehospital care

As well as the issues with EMS timeliness, the care provided by EMS providers is limited by the training they receive, as well as the lack of equipment to provide early resuscitation.

"It's not only death, we can also prevent the sequelae people come in with…they get brain damage, because the care was too late. The thing of the transport is very important, because the other guys, the EMS, if something happens, they are not well trained to resuscitate those patients. I've seen people die here because of that." – Medical officer

Receiving care

Poorly staffed facilities

In the district hospitals, there is a shortage of staff to provide care. The Casualty departments are largely run by clinical associates or junior doctors, who sometimes have difficulty getting senior supervision. Also, in the regional hospital, there is one orthopaedic surgeon and one general surgeon. In the tertiary hospital, there are more orthopaedic and general surgeons but only one neurosurgeon. When these experts are not available, care for the patients is severely delayed.

"From my side, as a clinical associate, some of the patients, you need a second opinion from a doctor or something like that, but it is almost impossible, if not impossible, to get that person whom you can talk to about the patient. So, that's the biggest challenge."— Clinical associate

Inadequately trained staff

An important recurring theme was the need for more training. Many HCPs did not receive standardized training in trauma care e.g. ATLS courses and those that did expressed the lack of continued learning. Not only are there barriers of poor staffing that limit the ability to explore training options located in other provinces, but there are also financial barriers, as HCPs said they financed the course and travel costs independently.

"There is no specific ATLS course given to us, actually in the province as a whole. I understand that in Guateng, and in Limpopo maybe. In other hospitals, they send their doctors, especially their junior doctors, to other places, to do their ATLS training, but with us, there is not that. So, you have to be organized and find out when an ATLS course

For peer review only

> *is going to be, find out for yourself and pay for yourself. Which is not convenient also for the hospital … you cannot go away for a week … how many of us… let's say it's me and my colleague who decide to go. That means for that week, we will leave a hole in the hospital, leave people struggling here, while you are out to educate yourself. Yes, so it is not the best."*—Medical officer

> *"…now after you've stabilized your patient and there is another trauma case, you have to take your team with you, but someone has to stay and take care of the first patient and now you're faced with a situation where you have to give the most junior person the responsibility of watching the vitals, but they don't really know what they are watching and the next thing you know, the machine alarms go off … they do not know what they are watching… they do not know that the [oxygen] saturation is not supposed to drop below 85% and then eventually you find yourself certifying a patient who … (heavy sigh)… you see what I mean?"*—Medical officer

Lack of equipment and supplies
Given financial constraints, there is often a lack of important medical equipment and supplies. When they are present, they easily become non-functional and are not repaired or replaced on time.

> *"…the second problem is life support equipment. Today it is there, tomorrow, it is broken down, yes …there is a blood corner, where we keep emergency blood, maybe two or three units in a fridge."*—Medical officer

Difficult referral system
A major problem in the care of severely injured patients, who are most commonly neurotrauma patients, is the ability to transfer them to a tertiary care institution, which is the only location for a computed tomography (CT) scan and access to a neurosurgeon. Some of the barriers include miscommunication about which hospitals receive referrals from the district hospitals and limited number of ambulances, especially ICU ambulances.

> *"the problem with serious trauma is the referral. When you have to refer to a higher institution, it's a hassle, it's a problem"*—Clinical associate

> *"Ok, uhh, the first, the big barrier, is the referral system. I can have a patient here, head injury, low GCS, they bring the patient in respiratory distress. I'll try to stabilize the patient. I'll try to stop the bleeding, stabilize the patient, tube the patient, and then…I'll get the doctors at the higher level, they will accept the patient, but to transfer that patient, I need an ICU ambulance but in the province, there are only two ICU ambulances. So, the ICU ambulance probably has to leave Witbank to come here, maybe it can take three to four hours. Then take the patient from here to Nelspruit, that is six hours, then it is a problem…especially since we don't have good ventilation equipment."*—Medical officer

For peer review only

> *"Well, sometimes, it's difficult because our referral system is contradictory. Like last week, we were surprised that our referrals should be going to Themba hospital. Meanwhile, we have been arranging them with Mapulaneng …They said that memo had been written in March, but we didn't know about it. Now, when we communicate with Themba, like last night, the doctor on call was trying to arrange with Themba for an acute abdomen…but they wanted him to send to Mapulaneng…it's still a bit of a confusion. It's difficult."*—Medical officer

Staff morale

Given the numerous challenges described above, the HCPs described feelings of frustration and disappointment with the amount of care they could provide, especially knowing that in better circumstances, they could save more lives.

> *"If you get an ICU ambulance, you have to get the intermediate trained ICU paramedics, or you have to get a chopper and that takes long so honestly, there is only so much you can do here. Because after you've given your drugs and you've intubated and ventilated, what more can you do? You're just sitting there and the CSF is just leaking and then 6 hours later, they come and you know, come on, you think, why are we even doing this? Then they go there, they do the CT scan and then they find that there is no neurosurgeon on call, so what do they do now? They just send the patient back. So, in terms of trauma, we still have a long way to go."*—Medical officer

For peer review only

BMJ Open

EXTERNAL INJURIES, TRAUMA AND AVOIDABLE DEATHS IN AGINCOURT, SOUTH AFRICA: A RETROSPECTIVE OBSERVATIONAL AND QUALITATIVE STUDY

Journal:	*BMJ Open*
Manuscript ID	bmjopen-2018-027576.R1
Article Type:	Research
Date Submitted by the Author:	01-Mar-2019
Complete List of Authors:	EDEM, IDARA; UNIVERISITY OF OTTAWA, NEUROSURGERY<br>Dare, Anna; University of Toronto Faculty of Medicine, Department of Surgery<br>Byass, Peter; Umea Universitet, Umeå Centre for Global Health Research<br>D’Ambruoso, L; University of Aberdeen School of Medicine and Dentistry, Centre for Global Development and Institute of Applied Health Sciences<br>Kahn, Kathleen; University of the Witwatersrand School of Public Health<br>Leather, Andy; King’s Health Partners and King’s College London, King’s Centre for Global Health<br>Tollman, Stephen; University of the Witwatersrand School of Public Health<br>Whitaker , John; King's Health Partners, King's Centre for Global Health<br>Davies, Justine ; King's College London, Centre for Global Health
**Primary Subject Heading**:	Global health
Secondary Subject Heading:	Emergency medicine, Public health, Qualitative research, Epidemiology
Keywords:	ACCIDENT & EMERGENCY MEDICINE, EPIDEMIOLOGY, Quality in health care < HEALTH SERVICES ADMINISTRATION & MANAGEMENT, PUBLIC HEALTH, TRAUMA MANAGEMENT

SCHOLARONE™
Manuscripts

External injuries, trauma and avoidable deaths in Agincourt, South Africa: A Retrospective Observational and Qualitative Study

Idara Edem – 1; Anna Dare – 2; Peter Byass – 3, 4; Lucia D'Ambruoso – 3, 5; Kathleen Kahn – 3; Andy Leather – 6; Stephen Tollman – 3; John Whitaker -6; Justine Davies – 7, 3.

Authors are in alphabetical order, apart from first, second, and last authors.

1. Division of Neurosurgery, Department of Surgery, University of Ottawa, Ottawa, Ontario, Canada
2. Centre for Global Health Research, St. Michael's Hospital and University of Toronto, Toronto, Ontario, Canada
3.MRC/Wits Rural Public Health and Health Transitions Research Unit—School of Public Health, Faculty of Health Sciences, University of the Witwatersrand, Johannesburg/Acornhoek, South Africa
4. WHO Collaborating Centre for Verbal Autopsy, Epidemiology & Global Health, Dept. of Public Health & Clinical Medicine, Umeå University, Umeå, Sweden
5. Centre for Global Development, University of Aberdeen
7. Institute for Applied Health Research, University of Birmingham, Birmingham, UK

Corresponding Author
Idara Edem
725 Parkdale Avenue, WM 150F, Mailbox 411
Ottawa, Ontario, Canada
K1Y4E9
iedem@toh.ca
613-282-8690

For peer review only - http://bmjopen.bmj.com/site/about/guidelines.xhtml

ABSTRACT

Objective: Injury burden is highest in Low and Middle Income Countries (LMICs). To reduce avoidable deaths, it is necessary to identify health system deficiencies that prevent timely, quality care. We developed criteria to use Verbal Autopsy (VA) data to identify avoidable deaths and associated health system deficiencies.

Setting: Agincourt, rural Bushbuckridge municipality, Mpumalanga province, South Africa.

Participants: Agincourt Health and Socio-Demographic Surveillance System (HDSS) and Health Care Providers (HCPs) from local hospitals.

Methods: A literature review to explore definitions of avoidable deaths after external injury (including trauma) and barriers to access to care using the "three delays framework" (seeking, reaching, and receiving care) was performed. Based on these definitions, this study developed criteria, applicable for use with VA data, for identifying avoidable death and which of the three delays contributed to avoidable deaths. These criteria were then applied retrospectively to VA-defined External Injury Deaths (EIDs) from 2012-2015. The findings were validated by external expert review. Key informant interviews (KIIs) with Health Care Providers (HCPs) were performed to further explore delays to care.

Results: Using VA data, avoidable death was defined with a focus on injury survivability, using level of consciousness at the scene and ability to seek care post-injury as indicators. Of 260 EIDs (189 trauma deaths), there were 104 (40%) avoidable EIDs and 78 (30%) avoidable trauma deaths (41% of trauma deaths). Delay in receiving care was the largest contributor to avoidable EIDs (61%) and trauma deaths (59%) followed by delay in seeking care (24% and 23%) and in reaching care (15% and 18%). KIIs revealed context-specific factors contributing to the third delay, including difficult referral systems, and inadequately trained staff.

Conclusions: A substantial proportion of EIDs and trauma deaths were avoidable, mainly occurring due to facility-based delays in care. Interventions, including strengthening referral networks, may substantially reduce trauma deaths

Key words: Trauma, Avoidable death, Verbal autopsy, Rural, South Africa

For peer review only - http://bmjopen.bmj.com/site/about/guidelines.xhtml

ARTICLE SUMMARY: STRENGTHS AND LIMITATIONS OF THIS STUDY

- As far as we are aware, this is the first time that verbal autopsy data has been used to ascertain avoidable mortality and access to care after trauma.
- As well, it is the first time that the three delays framework for access to emergency medical care has been systematically applied to trauma and avoidable mortality, allowing for the quantification of the delays to trauma care.
- Although this study was performed in a single Health and Socio-Demographic Surveillance System (HDSS), the findings are novel, pertinent to the Sustainable Development Goals and the methodology can be more widely applied to other VA data worldwide.
- Key informant interviews of health care workers added useful information to the VA data, but this information is likely to be skewed towards healthcare facilities.
- The VA methodology involves interviews conducted by well-trained but non-medical personnel, and does not capture detailed vitals at the scene or the nature of pre-hospital and in-hospital emergency care received, which can result in missing important clinical aspects of the injury and the care sought.

For peer review only

For peer review only - http://bmjopen.bmj.com/site/about/guidelines.xhtml

INTRODUCTION

Injuries account for an estimated 5.8 million deaths per year, 32% more than HIV, malaria and TB, combined.[1] The burden of trauma deaths is largest in Low and Middle Income Countries (LMICs) which are also the most ill-equipped to manage this burden.[2] Although there has been a global drive for injury prevention and we are in the "decade of action for road safety", morbidity and mortality from injuries continues to rise.[3] Alongside injury prevention efforts, optimization of medical care provided after injury occurrence is important. In many LMICs, there are deficiencies in Emergency Medical Services (EMS) and definitive hospital care for the injured. Where present, these are hampered by limited geographical coverage, resources, and trained staff.[4]

In order to improve a health system's ability to manage a large burden of injuries, gaps in care provision must be identified to guide resource allocation. To assist with this, the concept of completely or partially avoidable deaths—given the provision of effective health care—can be used[5] in association with exploration of the factors which led to an avoidable death occurring.

The three-delays framework (delays in seeking, reaching and receiving care) for accessing safe, affordable, and timely medical care is regarded as a classic conceptualization of delayed care in emergency situations, reflected in its application in obstetric emergencies,[6] sepsis management,[7] perinatal care[8] and hip fractures.[9] To the best of our knowledge, the three-delays model has not previously been analytically applied to trauma, but given the similarities between trauma and other time-critical conditions in which it has been used,[6] it is an appropriate model for investigating gaps in the health system contributing to avoidable trauma deaths.

To explore avoidable trauma deaths, a complete understanding of deaths and the events surrounding these deaths is needed. A lack of Civil Registration and Vital Statistics (CRVS) systems in many LMICs,[10] coupled with limited physician-assigned cause of death documentation, means that the verbal autopsy (VA) has become a practical tool for ascertaining cause of death, including for injuries.[11] VAs have not previously been used to examine avoidable deaths in trauma.

Our aims were: 1) To develop and validate criteria applicable to VA data to estimate numbers of avoidable deaths in trauma and other external injures; 2) In cases where deaths were avoidable, to develop criteria for assessing where in a three delays framework delays leading to deaths occurred, and estimate the numbers of cases where such delays were experienced; 3) Perform qualitative interviews with HCWs to further explore delays to care; 4) To combine information from the literature, VA, and qualitative analysis to construct a three delay's framework applicable to avoidable mortality in trauma.

For peer review only - http://bmjopen.bmj.com/site/about/guidelines.xhtml

METHODS

Literature review

To determine definitions and criteria for avoidable mortality and factors contributing to the three delays in trauma and external injuries, PubMed, Ovid and EMBASE were searched using the terms: "trauma" AND "avoidable death" OR "preventable death" OR "mortality" OR "delays" and limited to papers published in English, from 1990 onwards, in HICs and LMICs. The titles and abstracts were reviewed to select papers addressing avoidable death in trauma and delays to care.

Setting

The study was performed in Agincourt, located in the rural Bushbuckridge municipality of Mpumalanga province, South Africa. A Health and Socio-Demographic Surveillance System (HDSS) has regularly collected household data on health and vital events (births, deaths, migrations) for over 115,000 people in a geographically defined area since 1992. All deaths identified during surveys are followed up and a VA is conducted within one year of the death.

Verbal Autopsy

VA involves interview of the lay-primary caregiver of the deceased by a trained fieldworker about signs, symptoms and circumstances surrounding death. The interviews are completed within 12 months of the death, using a validated questionnaire, with good previously demonstrated recall.[12] [12] Currently, a computer algorithm, InterVA-4, is utilized to determine cause of death, using standardized probabilistic models.[13]

VA data used in this study was collected using the WHO 2012 short-form. [14] In addition to standard medical questions to allow cause of death to be ascertained, this contains a *trauma module*—41 questions to further query circumstances surrounding trauma deaths—and 10 "circumstances of mortality" (COM) questions that address the "household, community and health systems determinants of health" [15] influencing mortality. In the trauma module, for each category of injury, there are further specific questions to pinpoint the exact details of the deaths. COM questions are grouped under 4 themes—recognition of severity, mobilizing assets to seek care, access to care, and quality of care. Additionally, VAs contain a free text summary of the circumstances surrounding deaths, obtained from the respondent, that allows information not captured in the survey questions to be ascertained.

Data from all VAs between 2012 and 2015 categorized as "external injury deaths" (EIDs)[1] by InterVA-4 were extracted.[13] InterVA-4 classifies as EIDs, poisonings, drownings, deaths from natural disasters and traumas—which, as a subclass of EIDs, include road traffic accidents, assaults, fires and falls. Additionally, free-text searches of the open free-text summary portion of the VAs were performed to identify trauma deaths that may have been incorrectly categorized. Search terms included: injury, trauma, fall, accident, traffic and fire.

Defining avoidable death and developing a three-delays framework

[1] EIDs correspond to external causes of death in chapter 12 of the WHO 2012 VA instrument (WHO, 2012).

For peer review only - http://bmjopen.bmj.com/site/about/guidelines.xhtml

Findings from the literature review informed which variables contained in the VA questions and factors extractable from the free text had utility for defining avoidable deaths and in the creation of a three-delays framework. In particular, each narrative in the free text was reviewed for details pertaining to location of injury or death, acuity or chronicity of injury, signs and symptoms after injury, ability to seek care, factors facilitating or inhibiting access to care, the provision of medical care, results of care delivered, and understanding of the effects of care on the patient's outcome.

VA reports on deaths from 2015 were first utilized to categorise deaths by avoidability and delay. The categorisations of avoidability and three delays in a subsample of 20 cases were then validated by external review with a researcher and trauma care provider (JW) familiar with the geographic context. Discrepancies were discussed until consensus was achieved in order to improve classification of subsequent data. Once agreement was reached on categorizing deaths by avoidability and then using the three delays framework, avoidable deaths and delays were discerned for all EIDs from 2012 to 2014. Where there were multiple delays contributing to the avoidable mortality, that which was found to be the most immediate factor was considered the primary contributing delay.

Qualitative analysis
Purposive sampling was utilized to identify emergency-care providers from local hospitals to participate in Key Informant Interviews (KIIs) and snowball sampling was used until responses reached saturation and no new ideas were revealed.[16] The interviews were face-to-face semi-structured and open-ended. They were completed in English, recorded on tape, transcribed, and transferred for analysis into NVivo version 11.4 (QSR International, 2017). Transcripts were analysed using framework methods [17] within a thematic analysis. This allowed for unanticipated meanings to emerge from the data and analysis, as well as for the data to be structured based on the overall aims and objectives of the study, thus permitting both inductive and deductive data analyses.[18] This was an iterative process, to ensure that the content of the narrative data was as fully represented as possible. Identified factors were then compared with those garnered from the literature review and the VA analysis.

Final three delays framework
The findings from the literature review, VA, and qualitative analyses were combined to construct a holistic three delays framework for assessing avoidable deaths in trauma.

Statistical considerations
This is an exploratory study and a power calculation was not performed. Data are described. Analysis was performed using SPSS Statistics software (IBM, version 24), NVivo version 11.4 (QSR International, 2017) and Microsoft Excel.

Ethics approval and consent
Ethics approval from the University of the Witwatersrand Human Research Ethics Committee to use data from the VAs in the Agincourt HDSS in secondary analyses had been previously

For peer review only - http://bmjopen.bmj.com/site/about/guidelines.xhtml

obtained (M960720 and M110138). All analysed VA data was anonymized and no individual deceased person or respondent was identifiable from the data used and presented.

Ethics approval for the independent KIIs was obtained from the University of the Witwatersrand Human Research Ethics Committee (M170269) and King's College London Research Ethics Committee (LRU-16/17-4313). Participation in the qualitative study was voluntary and informed consent was obtained for the interview and its recording. All interviews were confidential and narratives were anonymized.

Patient and Public Involvement Statement

Patients were not directly involved in this exploratory study and no new patient data was collected. However, although no patients were involved in this study, Agincourt employs community engagement officers to ensure that community members are aware of research being done in the area – even if community dwellers are not involved – and that results are fed-back to the community.

RESULTS

Literature review

Fifty-seven papers which explored the conceptualisations of avoidable death in trauma and delays in access to care were identified, as were nine additional papers from review of references sections. Of these, 43 papers were included in the literature review. Despite differences in the definition of avoidable death in trauma, common themes included: injury survivability, deviations from delivery of optimal care and implication of errors in care delivery on the death of the patient. These themes are also reflected in the WHO guideline for the definition of avoidable death in trauma—note that this guideline definition also includes calculations of the probability of survival and injury severity scores.[2] Definitions of injury survivability which do not use injury severity scores were based on vital signs at the scene;[19]being awake at the scene; [19-21]or being able to seek care after injury; or having reversible, stable,[22] or non-severe injuries involving a single-system.[23,21,19,20] Most studies used a combination of these definitions (see appendix 1 for details on approaches to determine criteria for injury survivability).

Relevant components of the three delays model identified in the literature were as follows: deciding to seek care (socio-economic and cultural factors, perceived accessibility and perceived quality of care e.g. perceived severity, transportation, costs, EMS transport protocols, reputation of previous experience); identifying and reaching care (actual accessibility factors e.g. EMS accessibility and timeliness of response, prehospital care); receiving adequate and appropriate treatment (actual quality of care factors e.g. poorly staffed facilities and inadequate management) (Appendices 1 and 2). See Appendix 3 for the full list of articles included in the literature review.

VA Data

For peer review only - http://bmjopen.bmj.com/site/about/guidelines.xhtml

Figure 1 shows VA numbers included in the study. Between 2012 and 2015, there were 3001 deaths; of these 260 deaths were categorized as EIDs (8.7% of total) of which 189 were due to trauma (Table 1). The sub-category distribution for both EIDs and trauma deaths are shown in Appendix 4. Traffic and assault injuries were the most common causes of EID and trauma deaths.

Table 1. Demographic characteristics of the EID and trauma groups, for all, avoidable, and non-avoidable deaths from 2012-2015

Category	EIDs	Trauma deaths	Avoidable EID	Non-avoidable EID	Avoidable trauma deaths	Non-avoidable trauma deaths
Total	**260**	**189**	**104**	**156**	**78**	**111**
**Age group**						
Older (>65 y)	19 (7.3%)	12 (6%)	12 (11%)	7 (4.5%)	9 (11%)	3(2.7%)
Mid-age (50-64 y)	27 (10%)	20 (11%)	10 (9.6%)	17 (11%)	8 (10%)	12(11%)
Adult (15-49 y)	188 (72%)	143 (76%)	74 (71%)	114 (73%)	57 (73%)	86 (78%)
Child (5-14 y)	14 (5.4%)	7 (3.7%)	4 (3.8%)	10 (6.4%)	1 (1.3%)	6(5.4%)
Under-5 (1-4 y)	10 (3.8%)	5 (2.6%)	3 (2.9%)	7 (4.5%)	2 (2.6%)	3(2.7%)
Infant (1-11 months.)	1 (0.4%)	1 (0.5%)	0	1 (0.6%)	0	1(0.9%)
Neonate (<28 days)	1 (0.4%)	1 (0.5%)	1 (1.0%)	0	1 (1.3%)	0
**Sex**						
Male	198 (76%)	147 (78%)	75 (72%)	123 (79%)	59 (76%)	88(79%)
Female	62 (24%)	42 (22%)	29 (28%)	33 (21%)	19 (24%)	23(21%)

Development of avoidable death and three delays criteria for use with VA

In 2015, out of 482 total deaths, there were 48 EID cases which were used to develop criteria for determining avoidability. Descriptive information on injury survivability were the only criteria available. Factors indicating survivability were: being alive at the scene, being able to seek help, or non-severe single-system injuries (Table 2). Twenty (42%) EIDs were determined to be avoidable (Table 1). The external reviewer agreed on classification of avoidability of death in 90% of cases.

From this 2015 VA data, there were several extractable factors relevant to each of the three delays (Table 2). There was 80% agreement with the external expert for classification of delays contributing to death. Further analysis and discussion of VA free-text data was performed

For peer review only - http://bmjopen.bmj.com/site/about/guidelines.xhtml

together, until consensus was reached and a three delay's framework using data relevant to VA was constructed. Data relevant only to the circumstances of mortality module are contained in Appendix 5.

Table 2. Details on the data sections of VAs used to define injury survivability and determine delays to care

DESCRIPTION	VA SECTIONS CONTAINING RELEVANT DATA	VARIABLES EXTRACTED
Injury survivability	Demographics	Age
	Acuity of death	Acute versus chronic injury/death
	Previous medical history	List of medical conditions
	Signs and symptoms	System-based list of signs and symptoms
	External injury classification	Category of injury
	Trauma module	Details of injury classification and mechanism of injury
	Free-text disease descriptions	Vitals at the scene<br>Injured body system<br>Signs and symptoms at the scene and at hospital presentation<br>Injury severity<br>Actions taken immediately after injury<br>Ability to seek care
First delay	Circumstances of mortality questions	Doubts about the need for care<br>Use of traditional medicine<br>Did not use a mobile phone to call for help
	Free-text disease descriptions	Healthcare literacy and perceived severity of the injury<br>Perceived costs of seeking care—transport, medical costs<br>Perceived quality of care

For peer review only - http://bmjopen.bmj.com/site/about/guidelines.xhtml

		Previous experiences with medical care
	Trauma module	Traffic accident location i.e. rural versus urban<br>Death at site or time of injury
Second delay	Circumstances of mortality questions	Did not use otorized transport to get to care<br>>2 hrs travel to care<br>Prohibitive costs<br>Did not travel to a hospital or health facility
	Free-text disease descriptions	EMS accessibility<br>EMS timeliness of response<br>EMS versus personal transport<br>Prehospital care
Third delay	Circumstances of mortality questions	Problems with admission<br>Problems with treatment (medical treatment, procedures, interpersonal attitudes, respect, dignity)<br>Problems with tests and medications
	Free-text disease descriptions	Inadequate and/or delayed diagnosis and/or treatment<br>Delayed inter-hospital transfers<br>Poorly staffed and/or equipped facilities
	Treatment and health care utilization	Medical and/or surgical treatment<br>Traditional medicine treatment<br>If medical and traditional medicine treatment sought, which was sought first?

For peer review only - http://bmjopen.bmj.com/site/about/guidelines.xhtml

		Medical aid or employer support for healthcare<br>Medical actions<br>If no medical actions taken, reasons for not taking medical action

Avoidable mortality and three delays framework for EIDs and trauma, 2012-2015
Applying the definition of avoidable deaths to the 2012-2015 VA data revealed 104 avoidable EIDs (40%) and 78 avoidable trauma deaths (41%) (Table 1, Appendix 4).

Based on the three delays framework, 58% of people with avoidable EIDs experienced one delay, 38% two delays, and 4% three delays. For avoidable trauma deaths, 57% experienced one delay, 38% two delays, and 5% three delays. For all avoidable deaths, perceived injury severity, deficiencies in prehospital care, and inadequate and/or delayed diagnosis and/or treatments were the primary contributing factors in delays 1, 2 and 3, respectively (Table 3). Delay three was the most commonly occurring contributing delay to avoidable deaths, in 61% and 59% of EIDs and trauma deaths, respectively.

Table 3. Primary contributing factors in each delay category for avoidable EID and trauma deaths, 2012-2015.*

**Delay**	**Number (%)**	**Primary contributing delay factors**	**Number (%)**
**Avoidable EIDs (n=109)**			
1—Seeking care	25 (24%)	Perceived severity<br><br>Perceived etiology	35 (74%)<br><br>6 (12.7%)
2—Reaching care	16 (15%)	Prehospital care<br><br>EMS timeliness and response	28 (61%)<br><br>17 (37%)
3—Receiving care	63 (61%)	Inadequate and/or delayed diagnosis and/or treatment<br><br>Delayed inter-hospital transfer	82 (78%)<br><br>11 (10%)
**Avoidable trauma deaths (n=81)**			
1—Seeking care	18 (23%)	Perceived severity<br><br>Delayed discovery	23 (77%)<br><br>3 (9.6%)

For peer review only - http://bmjopen.bmj.com/site/about/guidelines.xhtml

2—Reaching care	14 (18%)	Prehospital care<br><br>EMS timeliness and response	25 (63%)<br><br>13 (34%)
3—Receiving care	46 (59%)	Inadequate and/or delayed diagnosis and/or treatment<br><br>Delayed inter-hospital transfer	59 (77%)<br><br>9 (11%)

Note that one individual may experience more than one delay.

When considering just the circumstances of mortality indicators (appendix 5), these captured minimal data about the quality of care. Also, there were no cases where respondents reported having "greater than 2 hours' travel to care" or "doubts about the need for care". However, a mobile phone was not used to call for help in 26% of EID cases and 28% did not travel to a hospital for care. For avoidable trauma deaths, 27% did not use a phone to call for help and 27% did not travel to a hospital for care. Also, issues with the prohibitive costs of seeking care were present in 20% of the avoidable EIDs and 21% of the avoidable trauma deaths.

KIIs and qualitative analysis

Seven KII were needed to reach saturation; these were done with two clinical associates (three-year training for a Bachelor of Clinical Medical Practice), four medical officers (four-year medical degree, two-year internship, one-year community service), and one Casualty (ER) manager working in the three hospitals in Bushbuckridge: Matikwane, Tintswalo and Mapulaneng. Important themes included healthcare literacy, EMS versus personal transport, mistrust of clinics (delay 1); EMS accessibility and timelines, prehospital care (delay 2); poorly staffed facilities, inadequately trained staff, lack of equipment and supplies, difficult referral systems and staff morale (delay 3). See below and appendix 6 for full details on these themes).[2]

Seeking care

Health or healthcare literacy

KIs felt that most patients were unaware of proper channels for seeking care, especially when an injury was not acutely severe.

> *"So, there are those who say straight to the hospital and those who say no but you can never actually tell which one because they don't know themselves… it's just a matter of their specific attitude, and there are mistakes in both ways …"*—Medical officer

EMS vs. personal transport

[2] Note that an abridged version of the KII data analysis is presented here, but more details are presented in Appendix 6.

For peer review only - http://bmjopen.bmj.com/site/about/guidelines.xhtml

KIs recounted that patients are aware of delayed responses from EMS and previous experience with EMS delays may affect the decision to seek care.

> *"From what we've heard, it's delayed response from the ambulances, the emergency services …it's a problem… and also for the patients, I think because this place is surrounded by villages…if they don't have transport, their own transport to come here, it becomes a bit of a challenge"* –Clinical associate

Reaching care

EMS accessibility and timeliness

The KIs felt that the largest barrier for patients reaching care was EMS accessibility. Given the rural area and the limited number of ambulances, several descriptions were provided of patients waiting for long periods to be taken to hospital.

> *"… the biggest problem I think they have is access to emergency care personnel, the paramedics, the ambulance. People may have trauma cases and when they call, the ambulance is already out, attending to trauma care somewhere else. It might take two to three hours for this ambulance to come and pick this patient. Now that is just too long for some cases. That might mean the difference between being normal again and being invalid or even dead."*—Medical officer

Prehospital care

As well as the issues with EMS timeliness, the care provided by EMS providers was described as limited by the training received, as well as by lack of equipment to provide early resuscitation.

> *"It's not only death, we can also prevent the sequelae people come in with…they get brain damage, because the care was too late. The thing of the transport is very important, because the other guys, the EMS, if something happens, they are not well trained to resuscitate those patients. I've seen people die here because of that."* –Medical officer

Receiving care

Inadequately trained staff

A key recurring theme was the need for more training. Many KIs reported that they did not receive standardized training in trauma care (e.g. Advanced Trauma Life Support [ATLS] courses) and those that did expressed the lack of continued learning.

> *"There is no specific ATLS course given to us, actually in the province as a whole…In other hospitals, they send their doctors, especially their junior doctors, to other places, to do their ATLS training, but with us, there is not that. So, you have to be organized and find out when an ATLS course is going to be, find out for yourself and pay for yourself. Which is not convenient also for the hospital … you cannot go away for a week…let's say it's me and my colleague who decide to go. That means for that week, we will leave a hole in the hospital."*—Medical officer

For peer review only - http://bmjopen.bmj.com/site/about/guidelines.xhtml

Lack of equipment and supplies

KIs reported that given financial constraints, there is often a lack of important medical supplies and when they are present, they easily become non-functional without being repaired or replaced on time.

> *"…the second problem is life support equipment. Today it is there, tomorrow, it is broken down, yes …there is a blood corner, where we keep emergency blood, maybe two or three units in a fridge."*—Medical officer

Difficult referral system

A major problem in the care of severely injured patients, who are most commonly neurotrauma patients, is the ability to transfer them to a tertiary care institution, which is the only location for a computed tomography (CT) scan and access to a neurosurgeon. KIs recounted that some of the barriers include miscommunication about where to transfer patients to and limited ICU ambulances.

> *"Ok, the first, the big barrier, is the referral system. I can have a patient here, head injury, low GCS [Glasgow Coma Scale], they bring the patient in respiratory distress. I'll try to stabilize the patient…tube the patient…I'll get the doctors at the higher level, they will accept the patient, but to transfer that patient, I need an ICU ambulance but in the province, there are only two ICU ambulances…maybe it can take three to four hours…Then take the patient from here to [the other hospital], that is six hours, then it is a problem…especially since we don't have good ventilation equipment."*—Medical officer

> *"Well, sometimes, it's difficult because our referral system is contradictory. Like last week, we were surprised that our referrals should be going to [this] hospital. Meanwhile, we have been arranging them with [another hospital] …They said that memo had been written in March, but we didn't know about it."*—Medical officer

Composite three-delays framework for avoidable deaths in trauma

The creation of the final three-delays framework for avoidable death in trauma based on analysis of information from three methodologies (literature review, VA analysis, and KIIs) is shown in Figure 2.

DISCUSSION

Between 2012 to 2015, a large proportion of external injury and trauma deaths were avoidable. Most avoidable EID and trauma deaths occurred in male adults between 15-49 years old and were a result of traffic injury or assault. A delay in receiving definitive care (the 'third delay') was the largest contributing factor in avoidable deaths, and occurred as a result of delays in diagnosis and treatment, inadequate referral systems for injured patients and poorly staffed and equipped health facilities, particularly at the district hospital level. These findings

For peer review only - http://bmjopen.bmj.com/site/about/guidelines.xhtml

demonstrate that VA data examining the nature of the injury and circumstances around death can be used to determine avoidable injury deaths and explore the factors contributing to these. Qualitative information from local health care providers provided useful additional information.

Globally, there is an increasing prevalence of trauma morbidity and mortality, especially in people younger than 45 years old,[24] with road traffic injuries being the second leading cause of death in 15-49 years old and the first in males.[25,26] In Mpumalanga, road traffic injuries were the second leading cause of years of life lost in 2012, after HIV/AIDS.[27] Trauma burden is worse in LMICs, given the limited scope of trauma systems and the challenges of service delivery in already strained health systems.[4] When injuries do occur, it is important to have trauma systems in place that ensure that the injured person receives care from the time of injury occurrence, to reduce avoidable mortality. The logical way to develop such systems in countries with nascent health care systems is to first identify gaps in current services. To our knowledge, this is the first time that data from VAs has been used to do this. Findings from this study should contribute to future local health system planning and create a path for future studies at other HDSSs collecting VA data.

Avoidable mortality was identified using data available from VA with criteria informed by a review of the literature. In trauma, there is heterogeneity in methods for categorizing avoidable deaths, but injury survivability—defined using injury severity scores and expert panels—is the commonest methodology.[28] Although injury survivability scores were not available in this study, VA contained data which were used – with good agreement – to define survivability of injury. It is reassuring that identified themes here are also reflected in the WHO guideline for the definition of avoidable death in trauma. The results on rates of avoidability are also similar to findings from other LMICs.[4,29-31] However, the avoidable death rate found in this study is greater than that found in high income countries.[28,32-34] The findings that most avoidable deaths were in the group defined by VA as adults (between 15 and 49 years old) and in males are also consistent with previous studies on trauma deaths.[21,35,24]

In this study, delays in receiving care could not be ascertained from VA review alone and KIIs provided useful additional data. HCPs revealed that factors including difficult referral systems, poorly staffed facilities, inadequately trained staff, and lack of equipment and supplies, all contributed to delayed or inadequate diagnosis and/or treatment, and thus avoidable death. These factors have also been shown to be contributors to avoidable trauma deaths in other studies [36-39] and they represent possible intervention points. Potentially, the widespread introduction of trauma training, including ATLS and training provided to HCPs in LMICs by institutions like Primary Trauma Care Foundation, could lead to reduced trauma avoidable mortality, as has been seen in other studies.[40] A review of the interventions to improve trauma care quality in LMICs, their cost-effectiveness and possible application in this setting, may be a worthwhile future study to pursue. Although the delays are presented linearly to enable understanding, they are actually interconnected, as delays in reaching and receiving care at one instance will influence the decision to seek care in future instances. Therefore, what is determined to be the most immediate factor contributing to death and thus the largest contributing delay, may actually be the outcome of the effect of other delays in the past. As

For peer review only - http://bmjopen.bmj.com/site/about/guidelines.xhtml

well, patients cycle through the delays model as their care plans change and they require transfer to other facilities, such that these patients face new and compounding delays as their care provision progresses.

The result that a substantial proportion of mortality is avoidable seems at odds with the findings in a recent study showing that, in South Africa, only 5.2% of the population is estimated to be outside a 2-hour travel time to emergency care.[41] These results are reflected in the analysis of the COM data where there were no reports of being outside a 2-hour travel distance to care. Yet, on examination of other parameters, including the free text, delays in reaching care – especially *appropriate* care – did contribute to avoidable mortality. Indeed, avoidable mortality may be best reduced by introducing effective prehospital trauma care [36] and by ensuring that a bypass protocol is implemented,[42] so that patients with severe trauma injuries are transported immediately to secondary or tertiary hospitals without first being delayed in district facilities. Many severely injured patients in this study experienced a substantial delay when sent first to district hospitals before awaiting transfer to regional or tertiary hospitals. This was corroborated by information from VA narratives and KIIs with emergency care providers. The goal of a well-functioning trauma system should be that the "right patient is treated at the right hospital at the right time".[43] So, if patients are severely injured, they are taken directly to hospitals with appropriate specialists. This has the advantage of eliminating inter-hospital transfer delays. However, the success of a trauma system with targeted patient transfers also depends on an appropriately equipped and staffed prehospital care system, which is lacking in many LMICs, as shown here.

Neurotrauma was a significant cause of avoidable trauma deaths in this study, as seen both from findings of the KIIs and VA review, where 35% of the avoidable trauma deaths had neurotrauma. This finding is likely an underestimation, given the lack of detailed medical information. Other studies have also found that most of the avoidable deaths in trauma are due to neurotrauma,[44,19] usually related to poor airway management.[45,24] In Bushbuckridge, where access to a neurosurgeon occurs only in a tertiary hospital outside of the municipality, it is paramount to provide adequate prehospital care, including proper airway management, as well as reduce inter-hospital transfer delays.

This study had several limitations. VA data does not capture detailed vitals at the scene, exact pre-hospital delays, or the nature of pre-hospital and in-hospital emergency care received. Therefore, although non-severe single-system injuries was used as a criteria for avoidable deaths, many more deaths could have occurred due to missed major secondary injuries, information that may not be well captured in VA data. Furthermore, the circumstances of mortality indicators provided information for the delays, but they did not capture important information about delays in reaching appropriate or quality care. The use of these indicators in South Africa was analysed in the census rounds of 2012-2013 and the researchers found that problems with not calling for help or not going to a medical facility were more pronounced than problems with overall costs in the younger age groups and in those with acute conditions, as in most trauma cases.[15] In this study, KIIs added useful information to the VA data, but this information is likely to be skewed towards healthcare facilities. If other service providers or

For peer review only - http://bmjopen.bmj.com/site/about/guidelines.xhtml
For peer review only

users were interviewed, further information about delays to access in care which are not contained in VA data may have been gleaned. An additional limitation is that the acute nature of trauma may also bias caregivers to report the care provided in hospital in more detail, as was seen in the free-text, so that if not asked specifically about the pre-hospital delays, these are not recollected or recorded. The VA methodology involves interviews conducted by well-trained but non-medical personnel, which can result in important clinical aspects of the injury and the care sought being missed. Since the VA interviews are conducted months after the death of the individual, recall bias becomes a major factor, affecting the reliability and validity of the information provided. Yet, although the VA is not the ideal tool for information on death and causes of death, it is a pragmatic method that provides useful information and its use continues to widen with greater innovations for its application.[15]

CONCLUSION

In this study, VA was shown to be a feasible method for defining avoidable deaths in trauma and ascertaining which of the three delays contribute to death. Between 2012 and 2015, a large percentage of external injury (40%) or trauma (41%) deaths were avoidable. The third delay in the three-delays model was found to be the largest contributor to avoidable deaths and the qualitative study supported the findings from the VA. To combat the burden of avoidable trauma deaths in LMICs, there is a need for a functioning trauma system, with adequate health systems interventions in trauma prevention, access to medical care, EMS and adequately staffed and supplied hospitals. “When it comes to trauma care, where people live ought not to determine if they live”.[46]

FIGURE LEGENDS

Figure 1. Deaths considered in this study (EIDs and trauma), relative to total deaths in the HDSS

Figure 2. Composite three-delays framework for avoidable death in trauma.

*The most frequently cited factors from the VA analysis and KIIs analysis are shaded in blue.

ACKNOWLEDGMENTS

We thank Chodziwadziwa Kabudula (MRC/Wits Rural Public Health and Health Transitions Research Unit—School of Public Health, Faculty of Health Sciences, University of the Witwatersrand, Johannesburg/Acornhoek, South Africa) for his assistance with assembling the Agincourt HDSS dataset for our use.

This work has been previously presented at the following conferences: AANS 2018, New Orleans, USA and CNS Bethune Round Table 2018, Toronto, Canada.

For peer review only - http://bmjopen.bmj.com/site/about/guidelines.xhtml

FUNDING

Funding for part of this project (travel scholarship) was provided by the King's College London, Centre for Global Health. This funding source was not involved in the study design; in the collection, analysis and interpretation of the data; in the writing of the manuscript; and in the decision to submit the paper for publication. Otherwise, this research received no specific grant from an agency of the public, commercial or not-for-profit sectors.

COMPETING INTERESTS

The authors do not report any competing interests or conflicts of interest.

CONTRIBUTIONS

IE helped to develop the idea, did the analysis, drafted and revised the paper; AD helped to develop the idea, and inputted into the paper drafts; PB inputted into the idea and the paper drafts; LD inputted into the idea, supported the analyses, and inputted into the paper drafts; AL, KK, and ST inputted into the idea and the paper drafts; KK set up and is overall responsible for the system of verbal autopsies; ST set up and is overall responsible for the Agincourt HDSS; JW contributed to the analyses and inputted into the paper drafts; JD led the project, developed the idea, supported the analyses, and inputted into the paper drafts; all authors approve the final paper for submission.

DATA SHARING

The data used for the VA analysis is available from the INDEPTH Data Repository Platform at: http://www.indepth-ishare.org/index.php/home
More data is available by emailing: iedem@toh.ca

For peer review only

REFERENCES

1. Lozano R, Naghavi M, Foreman K et al. Global and regional mortality from 235 causes of death for 20 age groups in 1990 and 2010: A systematic analysis for the Global Burden of Disease Study 2010. *Lancet* 2012;380:2095–128 doi: 10.1016/S0140-6736(12)61728-0

2. World Health Organization. Guidelines for Trauma Quality Improvement Programmes. Edited by C. Mock, C. Juillard, S. Brunage, J. Goosen, and M. Joshipura. Geneva: World Health Organization. 2009 doi: 10.1016/j.puhe.2010.01.011.

3. World Health Organization. *Saving Millions of lives: Decade of action for road safety 2011-2020, WHO publication*. Available at: http://who.int/violence_injury_prevention/publications/road_traffic/saving_millions_lives_en.pdf. Accessed March 2017.

4. Yeboah D, Mock C, Karikari P et al. Minimizing preventable trauma deaths in a limited-resource setting: A test-case of a multidisciplinary panel review approach at the Komfo Anokye Teaching Hospital in Ghana. *World J Surg* 2014;38:1707-12. doi: 10.1007/s00268-014-2452-z.

5. Rutstein D, Berenberg W, Chalmers T et al. Measuring the quality of medical care. A clinical method. *N Engl J Med* 1976;294:582-88.

6. Thaddeus S and Maine D. Too Far To Walk: Maternal Mortality in Context. *Soc Sci Med* 1994;38:1091–110.

7. Papali A, McCurdy M, Calvello E. A "three delays" model for severe sepsis in resource- limited countries. *J Crit Care* 2015;30:e9-14 doi: 10.1016/j.jcrc.2015.04.003.

8. Musafili A, Persson L, Baribwira C. et al. Case review of perinatal deaths at hospitals in Kigali, Rwanda: Perinatal audit with application of a three-delays analysis. *BMC Pregnancy and Childbirth* 2017;17:1–13. doi: 10.1186/s12884-017-1269-9.

9. Tewari A, Rath S, Yadav L et al. Care Seeking Behaviour of Older Adults with Hip Fracture in India: A Qualitative Study. *J Pat Care* 2017;3:128. doi: 10.4172/2573-4598.1000128.

10. Mikkelsen L, Phillips D, AbouZahr C et al. A global assessment of civil registration and vital statistics systems: monitoring data quality and progress. *Lancet* 2015;6736: 60171– 4.

11. Byass, P. Minimally Invasive Autopsy: A New Paradigm for Understanding Global Health? *PLoS Medicine* 2016;13: 8–11. doi: 10.1371/journal.pmed.1002173.

12. Kahn K, Collinson M, Gomez-Olive F et al. Profile: Agincourt health and socio- demographic surveillance system. *Int J Epidemiol* 2012;41:988–1001.

For peer review only - http://bmjopen.bmj.com/site/about/guidelines.xhtml

13. Murray CJ, Lozano R, Flaxman AD et al. Robust metrics for assessing the performance of different verbal autopsy cause assignment methods in validation studies. *Popul Health Metr* 2011;9:28.

14. World Health Organization. Verbal autopsy standards: The 2012 WHO verbal autopsy instrument. Geneva. Available at: http://www.who.int/healthinfo/statistics/verbalautopsystandards/en/index2.html. Accessed January 2017.

15. D'Ambruoso L, Kahn K, Wager RG et al. Moving from medical to health systems classifications of deaths: extending verbal autopsy to collect information on the circumstances of mortality. *Glob Health Res Policy*. *Global Health Research and Policy* 2016;1:2. doi: 10.1186/s41256-016-0002-y.

16. Ritchie J, Lewis J. Qualitative research practice: A guide for social science students and researchers. Los Angeles: Sage Publishing Ltd 2014.

17. Ritchie J, Lewis J. Qualitative research practice: a guide for social science students and researchers. London: Sage Publishing Ltd 2003.

18. Gale N, Heath G, Cameron E et al. Using the framework method for the analysis of qualitative data in multi-disciplinary health research. *BMC Medical Research Methodology* 2013;13:117. doi: 10.1186/1471-2288-13-117.

19. MacLeod JBA, Cohn SM, Johnson EW et al. Trauma deaths in the first hour: are they all unsalvageable injuries? *Am J Surg* 2007;193:195–9. doi: 10.1016/j.amjsurg.2006.09.010.

20. Kleber C, Giesecke MT, Tsokos M et al. Trauma-related preventable deaths in Berlin 2010: Need to change prehospital management strategies and trauma management education. *World J Surg* 2013;37: 1154–61. doi: 10.1007/s00268-013-1964-2.

21. Ray JJ, Meizoso JP, Satahoo SS et al. Potentially preventable prehospital deaths from motor vehicle collisions. *Traffic Inj Prev* 2016;17:676–80. doi: 10.1080/15389588.2016.1149580.

22. Schoeneberg C, Schilling M, Probst T et al. Preventable and potentially preventable deaths in severely injured elderly patients: A single-center retrospective data analysis of a german trauma center. *World J Surg* 2014;38:3125–32. doi: 10.1007/s00268-014- 2755-0.

23. Papadopoulos IN, Bukis D, Karalas E et al. Preventable prehospital trauma deaths in a Hellenic urban health region: an audit of prehospital trauma care. *J Trauma* 1996;41:864–9.

24. Harris T, Davenport R, Hurst T et al. Improving outcome in severe trauma: trauma systems and initial management--intubation, ventilation and resuscitation. *Postgrad Med Journal*, 2012;88:588–94. doi: 10.1136/postgradmedj-2010-74245.

25. Institute for Health Metrics and Evaluation (IHME). GBD Compare. GBD 2016.

For peer review only - http://bmjopen.bmj.com/site/about/guidelines.xhtml

https://vizhub.healthdata.org/gbd-compare/. Accessed February 18, 2018.

26. Abajobir AA, Abbafati C, Abbas K et al. Global, regional, and national age-sex specific mortality for 264 causes of death, 1980–2016: a systematic analysis for the Global Burden of Disease Study 2016. *Lancet* 2017;390:1151–210. doi: 10.1016/S0140- 6736(17)32152-9.

27.Pillay-van Wyk V, Msemburi W, Laubscher et al. Mortality trends and differentials in South Africa from 1997 to 2012: second National Burden of Disease Study. *Lancet Glob Health* 2016;4:e642–e653. doi: 10.1016/S2214-109X(16)30113-9.

28.Oliver GJ and Walter DP. A Call for Consensus on Methodology and Terminology to Improve Comparability in the Study of Preventable Prehospital Trauma Deaths: A Systematic Literature Review. *Acad Emerg Med* 2016;23:503–10. doi: 10.1111/acem.12932.

29. Zafarghandi MR, Modaghegh MHS, Boudsari BS. Preventable Trauma Death in Tehran: An Estimate of Trauma Care Quality in Teaching Hospitals. *J Trauma* 2003;55:459–65. doi: 10.1097/01.TA.0000027132.39340.FE.

30. Moon S, Lee SH, Ryoo HW et al. Preventable trauma death rate in Daegu, South Korea. *Clini Exp Emerg Med* 2015;2:236–43. doi: 10.15441/ceem.15.085.

31. Iau PTC, Ong CL, Chan STF. Preventable trauma deaths in Singapore. *Aust N Z J Surg* 1998;68:820–5. doi: 10.1046/j.1440-1622.1998.01461.x.

32. Chiara O, Cimbanassi S, Pitidis A et al. Preventable trauma deaths: from panel review to population based-studies. *World J Emerg Surg* 2006;1:1–7. doi: 10.1186/1749-7922-1- 12.

33. Kwon AM, Garbett NC, Kloecker GH. Pooled preventable death rates in trauma patients: Meta analysis and systematic review since 1990. *Eur J Trauma Emerg Surg* 2014;40:279– 85. doi: 10.1007/s00068-013-0364-5.

34. Settervall CHC, Domingues C. de A, Cardoso de Sousa RM et al. Preventable Trauma Deaths. *Rev Saude Publica*, 2012;46:1–8. doi: 10.1001/jama.1985.03360080071032.

35. Castillo-Rodríguez L, Díaz-Jiménez D, Castañeda-Orjuela C. et al. Years of Life Lost (YLL) in Colombia 1998-2011: Overall and avoidable causes of death analysis. *PLoS ONE* 2015;10:1–11. doi: 10.1371/journal.pone.0125456.

36. Henry JA, Reingold AL. Prehospital trauma systems reduce mortality in developing countries. *J Trauma Acute Care Surg* 2012;73:261–8. doi: 10.1097/TA.0b013e31824bde1e.

37. Montmany S, Pallisera A, Rebasa P et al. Preventable deaths and potentially preventable deaths. What are our errors? *Injury* 2016;47:669-73

38. Sugrue M, Caldwell E, D'Amours S et al. Time for a change in injury and trauma care

For peer review only - http://bmjopen.bmj.com/site/about/guidelines.xhtml

delivery: A trauma death review analysis. *Aust N Z J Surg* 2008;78:949–54. doi: 10.1111/j.1445-2197.2008.04711.x.

39. Vioque SM, Kim PK, McMaster J. Classifying errors in preventable and potentially preventable trauma deaths: A 9-year review using the Joint Commission's standardized methodology. *American Journal of Surgery* 2014;208:187–94. doi: 10.1016/j.amjsurg.2014.02.006.

40. Navarro S, Montmany S, Rebasa P. Impact of ATLS training on preventable and potentially preventable deaths. *World J Surg* 2014;38:2273–8. doi: 10.1007/s00268-014- 2587-y.

41. Ouma PO, Maina J, Thuranira PN. Access to emergency hospital care provided by the public sector in sub-Saharan Africa in 2015: a geocoded inventory and spatial analysis. *Lancet Glob Health* 2018;2214–109. doi: 10.1016/S2214-109X(17)30488-6.

42. Goosen J, Bowley DM, Degiannis E et al. Trauma care systems in South Africa. *Injury*, 2003;34:704–8. doi: 10.1016/S0020-1383(03)00153-0.

43. McSwain N, Rotondo M, Meade et al. A model for rural trauma care. *Br J Surg* 2012;99:309–14. doi: 10.1002/bjs.7734.

44. Hussain LM, Redmond AD. Are pre-hospital deaths from accidental injury preventable? *Br Med J (Clin Res Ed)* 1994;308:1077–80. doi: 10.1136/bmj.309.6946.57.

45. London JA, Mock CN, Quansah RE. Priorities for improving hospital-based trauma care in an African city. *J Trauma* 2001;51:747–53. doi: 10.1097/00005373-200110000-00021.

46. Berwick DM, Downey AS, Cornett EA. A National Trauma Care System to Achieve Zero Preventable Deaths After Injury Recommendations From a National Academies of Sciences, Engineering, and Medicine Report. *JAMA* 2016;316:927–28. doi: 10.1001/jama.2016.8524.Conflict.

For peer review only

For peer review only - http://bmjopen.bmj.com/site/about/guidelines.xhtml

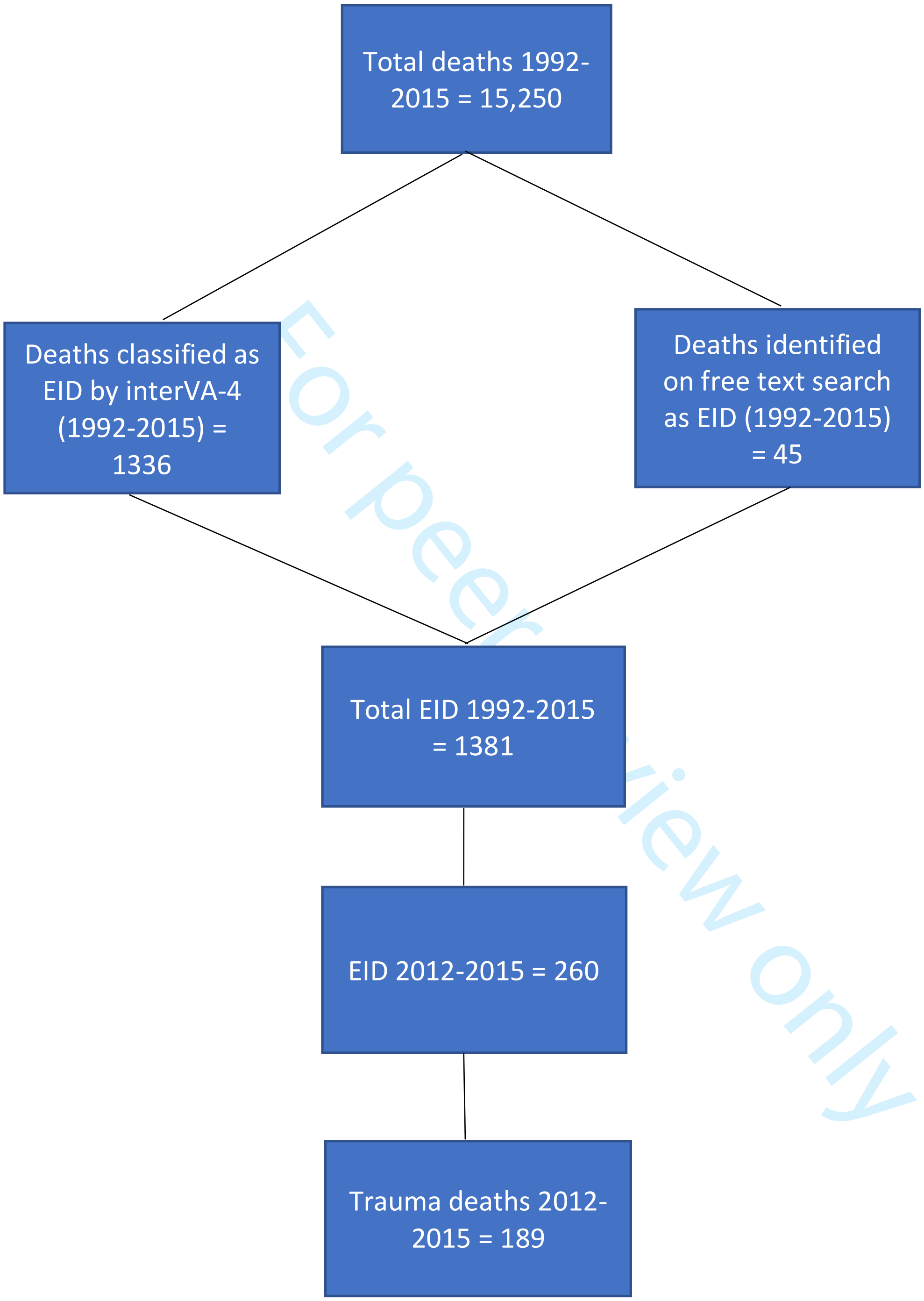

Figure 1. Deaths considered in this study (EIDs and trauma), relative to total deaths in the HDSS

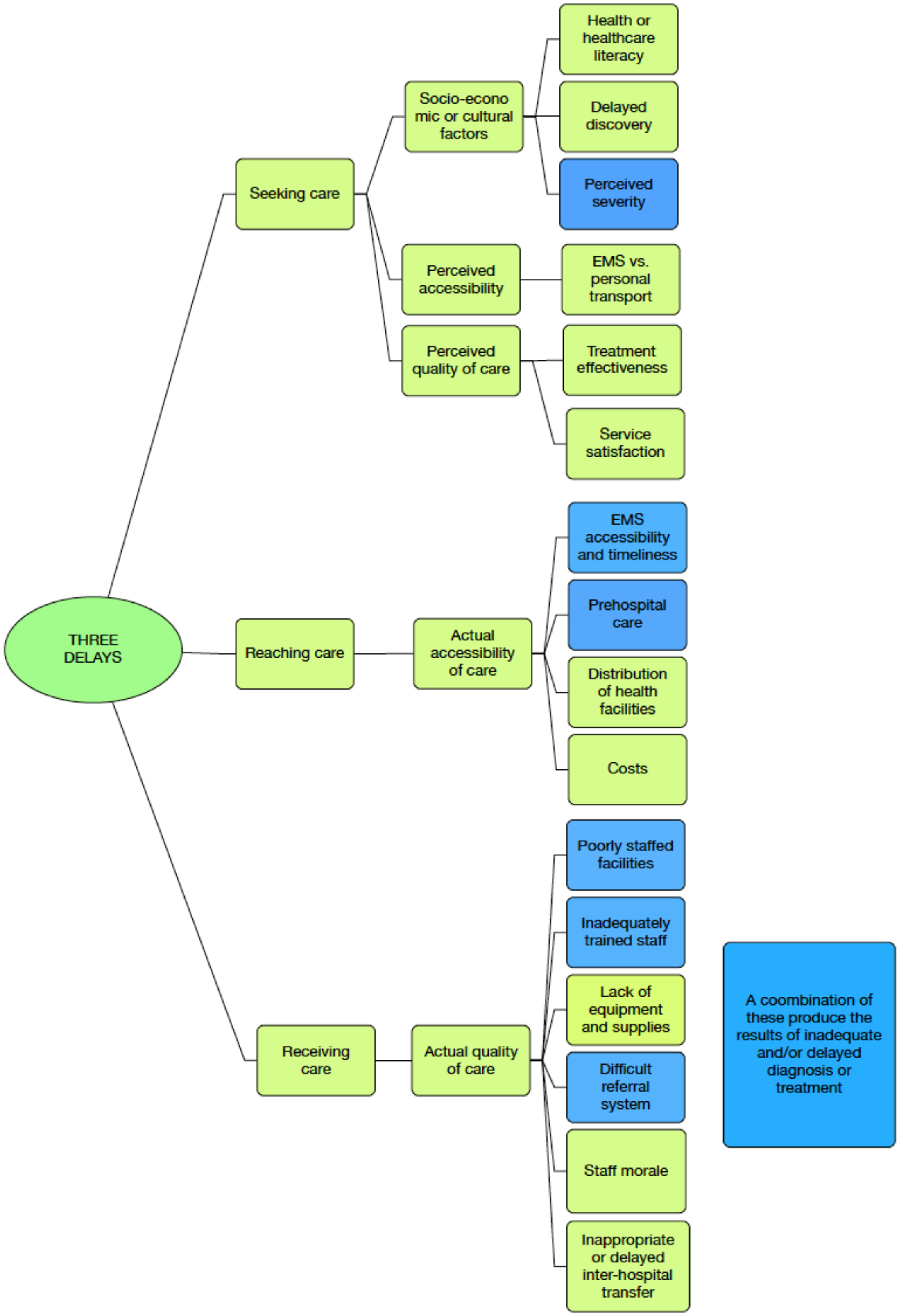

Figure 2. Composite three-delays framework for avoidable death in trauma.
*The most frequently cited factors from the VA analysis and KIIs analysis are shaded in blue.

Appendices

Appendix 1. Details of the definitions of injury survivability

DEFINITIONS OF SURVIVABILITY	STUDIES EXAMINING INJURY SURIVABILITY
Vitals at the scene of injury e.g. palpable pulse or blood pressure	[1]
Awake at the scene	[1-3]
Reversible or treatable injury	[4,3]
Generally stable at initial treatment or if unstable, becomes stable with initial treatment	[5]
Non-severe injuries	[3]
Non-severe single-system or single-organ or single-vessel injuries	[1,2]
Non-severe non-central nervous system (CNS) injuries	[3]
**APPROACHES TO CRITERIA FOR DEFINITION OF SURVIVABILITY**	**STUDIES**
Abbreviated Injury Scale (AIS)/Injury Severity Scores (ISS)/Probability of Survival (PS)/Trauma Score (TS)	[6-8]
AIS/ISS/PS and specific preventable factors	[9,10-12]
AIS/ISS/PS and impact of care delivered	[13-31]
Survivable injuries (subjective/panel)	[5]
Survivable injuries (subjective/panel) and impact of care delivered	[2,4,32-34,31,1,5,35,36,3]
Impact of care delivered	[37-38]

For peer review only

Appendix 2. Details of the three-delays model for care extracted from literature review*

DELAY	FACTORS AFFECTING UTILIZATION OR OUTCOME
DECIDING TO SEEK CARE	**Socio-economic/Cultural**<br>Illness factors<br>● Healthcare literacy<br>● Delayed discovery [4,14]<br>● Perceived severity<br>● Perceived etiology<br>Economic status [39]<br>Educational status
	**Perceived Accessibility**<br>Distance<br>Transportation [4]<br>● EMS versus personal transport**<br>● Road conditions<br>● Season—dry or rainy<br>Costs [39]<br>● Transportation<br>● Medical care<br>● Medications<br>● Opportunity costs<br>● Lost income<br>EMS transport protocols [39]<br>● Closest hospital versus best equipped hospital
	**Perceived quality of care**<br>Reputation or previous experience<br>Satisfaction with outcomes<br>● Treatment effectiveness<br>Satisfaction with service<br>● Staff attitudes<br>● Hospital capacity and surgery<br>● Supplies availability<br>● Waiting time<br>● Hospital efficiency<br>● Care consistent with local beliefs<br>● Timely care<br>● Visitation rules affecting social support
IDENTIFYING AND REACHING CARE	**Actual accessibility of care**<br>Distribution of health facilities [31,37,39]<br>EMS accessibility and timeliness of response [2,37]<br>Travel time [1,40,4,14]<br>Prehospital care [33,41,42,37,39]<br>Costs

For peer review only

	EMS transport protocols<br>● Closest hospital versus most equipped hospital [43]
RECEIVING ADEQUATE AND APPROPRIATE TREATMENT	**Actual quality of care**<br>Poorly staffed facilities [33]<br>● Number of staff/lack of personnel<br>● Competence of staff—training of personnel [40,20]<br>● Maldistribution of staff<br>● Lack of managers<br>Poorly equipped facilities [33,39]<br>● Lack of a blood bank<br>● Lack of a functioning operating room (OR)<br>● Lack of medications or supplies<br>● Lack of infrastructure<br>Inadequate management [33,2,4,20,21,31,42,43,22,37,39,27]<br>● Inadequate or delayed diagnosis<br>● Inadequate or delayed treatment<br>● Inappropriate or delayed inter-hospital transfer<br>● Functioning trauma system<br>● Ward care after ER treatment

*Model based on that created for obstetric emergencies (Thaddeus and Maine, 1994). Delay factors examined in other studies, with respect to trauma, are cited here.
**EMS refers not only to the mode of transportation, but also to the care provided while in transport. As perceptions of EMS can affect decisions to seek care, EMS appears in both seeking care and reaching care categories.

For peer review only

Appendix 3. References for articles in literature review.

1. MacLeod JBA, Cohn SM, Johnson EW et al. Trauma deaths in the first hour: are they all unsalvageable injuries? *Am J Surg* 2007;193:195–9. doi: 10.1016/j.amjsurg.2006.09.010.
2. Kleber C, Giesecke MT, Tsokos M et al. Trauma-related preventable deaths in Berlin 2010: Need to change prehospital management strategies and trauma management education. *World J Surg* 2013;37: 1154–61. doi: 10.1007/s00268-013-1964-2.
3. Ray JJ, Meizoso JP, Satahoo SS et al. Potentially preventable prehospital deaths from motor vehicle collisions. *Traffic Inj Prev* 2016;17:676–80. doi: 10.1080/15389588.2016.1149580.
4. Papadopoulos IN, Bukis D, Karalas E et al. Preventable prehospital trauma deaths in a Hellenic urban health region: an audit of prehospital trauma care. *J Trauma* 1996;41:864–9.
5. Schoeneberg C, Schilling M, Probst T et al. Preventable and potentially preventable deaths in severely injured elderly patients: A single-center retrospective data analysis of a german trauma center. *World J Surg* 2014;38:3125–32. doi: 10.1007/s00268-014- 2755-0.
6. Boman H, Bjornstig U, Hedelin A et al. Avoidable deaths in two areas of Sweden--analysis of deaths in hospital after injury. *European Journal of Surgery* 1999;165:828-33
7. Pellicane J, Byrnne K, DeMaria E. Preventable complications and death from multiple organ failure among geriatric trauma victims. *J Trauma* 1992;33:440-44
8. Oliver GJ, Walter DP, Redmond AD. Prehospital deaths from trauma: are injuries survivable and do bystanders help? *Injury* 2017;48:985-91
9. Hussain LM, Redmond AD. Are pre-hospital deaths from accidental injury preventable? *Br Med J (Clin Res Ed)* 1994;308:1077–80. doi: 10.1136/bmj.309.6946.57.
10. Diamond I, Parkin P, Wales P et al. Preventable pediatric deaths in Ontario: a comparative population-based study. *J Trauma* 2009;66:1189-94.
11. Lerer L, Knottebnelt J. Preventable mortality following sharp penetrating chest trauma. *J Trauma* 1994;37:9-12
12. Oliver GJ, Walter DP, Redmond AD. Are prehospital deaths from trauma and accidental injury preventable? A direct historical comparison to assess what has changed in two decades. *Injury* 2017;48:978-84
13. McDermott F, Cordner S, Tremayne A. Evaluation of the medical management and preventability of death in 137 road traffic fatalities in Victoria, Australia: an overview. Consultative Committee on Road Traffic Fatalities in Victoria. *J Trauma* 1996;40:520-33.
14. Esposito T, Sanddal N, Hansen J et al. Analysis of preventable trauma deaths and inappropriate trauma care in a rural state. *J Trauma* 1995;39:955-62.
15. Esposito T, Sanddal N, Dean J et al. Analysis of preventable pediatric trauma deaths and inappropriate trauma care in Montanna. *J Trauma* 1999;47:243-51.

16. Esposito T, Sanddal T, Reynolds S et al Effect of a voluntary trauma system on preventable death and inappropriate care in a rural state. *J Trauma* 2003;54:663-70.
17. Henriksson E, Ostrom M, Eriksson A. Preventability of vehicle-related fatalities. *Accid Anal Prev* 2001;33:467-75.
18. Rautji R, Bhardwaj D, Dogra T. The Abbreviated Injury Scale and its correlation with preventable traumatic accidental deaths: A study from South Delhi. *Medicine Science and the Law* 2006;46:157-65
19. Chiara O, Scott J, Cimbanassi S et al. Trauma deaths in an Italian urban area: An audit of prehospital and in-hospital trauma care. *Injury* 2002;33:553-62
20. Vioque SM, Kim PK, McMaster J. Classifying errors in preventable and potentially preventable trauma deaths: A 9-year review using the Joint Commission's standardized methodology. *American Journal of Surgery* 2014;208:187–94. doi: 10.1016/j.amjsurg.2014.02.006.
21. Montmany S, Pallisera A, Rebasa P et al. Preventable deaths and potentially preventable deaths. What are our errors? *Injury* 2016;47:669-73
22. Saltzherr T, Wendt K, Nieboer P et al. Preventabilityof trauma deaths in a Dutch level-1 trauma centre. *Injury* 2011;42:870-73.
23. Motomura T, Mashiko K, Matsumoto H et al. Preventable trauma deaths after traffic accidents in Chiba Prefecture, Japan, 2011: Problems and Solutions. *Journal of Nippon Medical School* 2014;81:320-27
24. Sampalis J, Boukas S, Nikolis A et al. Preventable death classification: Inter-rater reliability and comparison with ISS-based survival probability estimates. *Accident Analysis and Prevention* 1995;27:199-206.
25. Schoeneberg C, Schilling M, Hussmann B et al. Preventable and potentially preventable deaths in severely injured patients: a retrospective analysis including patterns of errors. *Eur J Trauma Emerg Surg* 2016;1-9
26. Moon S, Lee SH, Ryoo HW et al. Preventable trauma death rate in Daegu, South Korea. *Clini Exp Emerg Med* 2015;2:236–43. doi: 10.15441/ceem.15.085.
27. Ha M, Kim B, Choi S et al. Preventable and potentially preventable traumatic death rates in a neurosurgery department: a single centre experience. *Korean J Neurotrauma* 2016;12:67-71
28. Kuo L, Kaufman E, Hoffman R et al. Failure to rescue after injury is associated with preventability: the results of mortality panel review of failure to rescue cases in trauma. *Surgery* 2017;161:782-90.
29. 65. Kim Y, Jung K. Utility of the international classification of diseases injury severity score: detecting preventable deaths and comparing the performance of emergency medical centres. *J Trauma* 2003;54:775-80.
30. Iau PTC, Ong CL, Chan STF. Preventable trauma deaths in Singapore. *Aust N Z J Surg* 1998;68:820–5. doi: 10.1046/j.1440-1622.1998.01461.x.
31. Sugrue M, Caldwell E, D'Amours S et al. Time for a change in injury and trauma care delivery: A trauma death review analysis. *Aust N Z J Surg* 2008;78:949–54. doi: 10.1111/j.1445-2197.2008.04711.x.

For peer review only

32. Sanddal T, Esposito T, Whitney J et al. Analysis of preventable trauma deaths and opportunities for trauma care improvement in Utah. *J Trauma* 2011;70:970-77
33. Yeboah D, Mock C, Karikari P et al. Minimizing preventable trauma deaths in a limited-resource setting: A test-case of a multidisciplinary panel review approach at the Komfo Anokye Teaching Hospital in Ghana. *World J Surg* 2014;38:1707-12. doi: 10.1007/s00268-014-2452-z.
34. Zafarghandi MR, Modaghegh MHS, Boudsari BS. Preventable Trauma Death in Tehran: An Estimate of Trauma Care Quality in Teaching Hospitals. *J Trauma* 2003;55:459–65. doi: 10.1097/01.TA.0000027132.39340.FE.
35. Maxson T, Mabry C, Sutherland M. Does the institution of a statewide trauma system reduce preventable mortality and yield a positive return on investmnt for taxpayers. *J Am Coll Surg* 2017;224:489-99
36. Navarro S, Montmany S, Rebasa P. Impact of ATLS training on preventable and potentially preventable deaths. *World J Surg* 2014;38:2273–8. doi: 10.1007/s00268-014-2587-y.
37. Lauriola P, Tosatti F, Schiavi A et al. Confidential enquiry into avoidable vehicle accidental deaths in the province of Modena, Italy. *Eur J Epidemiol*, 2000;16:67-74.
38. Stocchetti N. Risk prevention, avoidable deaths and mortality-morbidity reduction in head injury. *Eur J Emerg Med* 2001;8:215-219.
39. Simons R, Brasher P, Taulu T et al. A population-based analysis of injury-related deaths and access to trauma care in rural-remote Northwest British Columbia. *J Trauma* 2010;69:11-9.
40. Chiara O, Cimbanassi S, Pitidis A et al. Preventable trauma deaths: from panel review to population based-studies. *World J Emerg Surg* 2006;1:1–7. doi: 10.1186/1749-7922-1-12.
41. Henry JA, Reingold AL. Prehospital trauma systems reduce mortality in developing countries. *J Trauma Acute Care Surg* 2012;73:261–8. doi: 10.1097/TA.0b013e31824bde1e.
42. Goosen J, Bowley DM, Degiannis E et al. Trauma care systems in South Africa. *Injury*, 2003;34:704–8. doi: 10.1016/S0020-1383(03)00153-0.
43. McSwain N, Rotondo M, Meade et al. A model for rural trauma care. *Br J Surg* 2012;99:309–14. doi: 10.1002/bjs.7734.

For peer review only

Appendix 4. Distribution of cause of death categories for EIDs, trauma, avoidable EIDs and avoidable trauma deaths, 2012-2015.

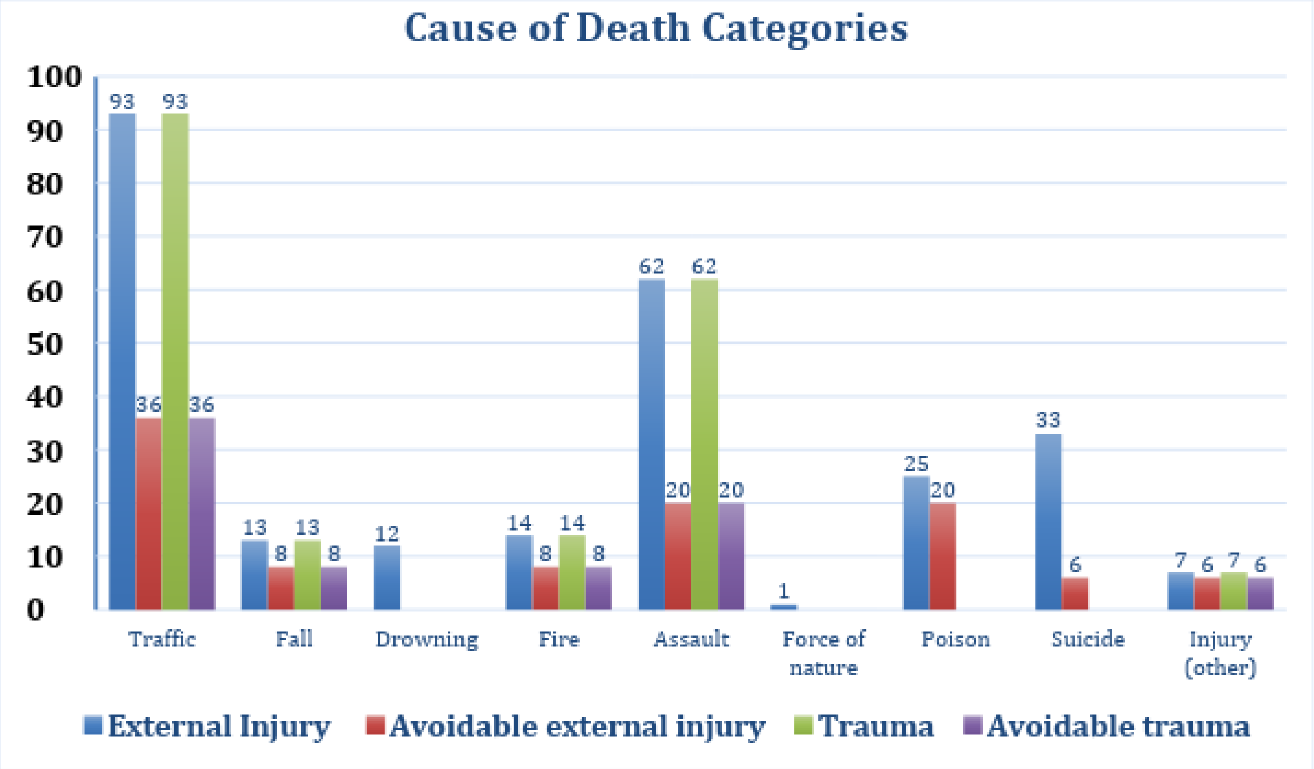

Injury (other) category reflects injuries from objects hitting patients e.g. a football.

Appendix 5. Details of the circumstances of mortality module for the avoidable EIDs (n=109) and trauma (n=81), 2012-2015

**Social module category**	**Indicator**	**EIDs n (%)**	**Trauma n (%)**
Recognition of severity	Doubts about the need for care	0 (0)	0(0)
	Use of traditional medicine	1(1.0)	1 (1.3)
Mobilising assets to seek care	Did not use a mobile phone to call for help	27 (26)	21 (27)
	Did not use motorised transport to get to care	24 (23)	18 (23)
Access to care	Prohibitive costs	21 (20)	16 (21)
	Did not travel to a hospital or health facility	29 (28)	21 (27)
	>2 hrs travel to care	0 (0)	0 (0)
Quality of care	Problems with admission	2 (1.9)	2 (2.6)
	Problems with treatment (medical treatment, procedures, interpersonal attitudes, respect, dignity)	9 (8.6)	8 (10.2)
	Problems with tests and medications	6 (7.5)	6 (10.2)

Note, for the quality of care indicators and "did not use motorized transport to get to care", n=80 for avoidable EIDs and n=59 for avoidable trauma deaths. This reflects the fact that the denominator in these cases is the total number of eopoe who traveled to hospital

For peer review only

Appendix 6. Details of the key informant interview results.

<u>Seeking care</u>

Health or healthcare literacy

For the general population, HCPs felt that most patients were unaware of proper channels for seeking care, especially when an injury was not acutely severe.

> *"So, there are those who say straight to the hospital and those who say no but you can never actually tell which one because they don't know themselves…it's just a matter of their specific attitude, and there are mistakes in both ways …"*—Medical officer

EMS vs. personal transport

HCPs know that patients are aware of delayed responses from EMS services, so most patients decide between waiting for EMS and seeking personal transport. Previous experience with EMS delays may affect the decision to seek care, as some patients will decide to seek care only when personal transport can be arranged. For those who can afford private ambulance services, this is an easier decision to make, as the private ambulances arrive earlier and are better equipped.

> *"From what we've heard, it's delayed response from the ambulances, the emergency services…the emergency service…it's a problem… and also for the patients, I think because this place is surrounded by villages…if they don't have transport, their own transport to come here, it becomes a bit of a challenge"* –Clinical associate

Mistrust of clinics

Although there may be clinics available, they are not open outside business hours and not well equipped, so patients often decide to wait in the Casualty department of district hospitals, even when their conditions may have been treated in clinics. Although seeking any kind of care after an injury is better than seeking no care at all, for most injuries, seeking care at a hospital may be more appropriate than seeking care in a clinic.

> *"…the patients …they come with their referral, but you will find that most of them don't have a referral, which means that they don't trust their clinic, the clinic for them. Even those cases that can be seen there, but most of the time, they are not seen there."* –Medical officer

> *"They don't really like going to the clinics, because to them, they feel like, they're going to send me to the hospital anyways, so they prefer coming straight to the hospitals, because they feel like they're wasting time."* –Medical officer

<u>Reaching care</u>

EMS accessibility and timeliness

The HCPs felt that the largest barrier for patients reaching care was EMS accessibility. Given the rural area and the limited number of ambulances, patients often wait a long time to be taken to hospital.

For peer review only

"… the biggest problem I think they have is access to emergency care personnel, the paramedics, the ambulance. People may have trauma cases and when they call, the ambulance is already out, attending to trauma care somewhere else. It might take two to three hours for this ambulance to come and pick this patient. Now that is just too long for some cases. That might mean the difference between being normal again and being invalid or even dead. So, that is it. It is just the availability of the service…it's not optimal."—Medical officer

Prehospital care
As well as the issues with EMS timeliness, the care provided by EMS providers is limited by the training they receive, as well as the lack of equipment to provide early resuscitation.

"It's not only death, we can also prevent the sequelae people come in with…they get brain damage, because the care was too late. The thing of the transport is very important, because the other guys, the EMS, if something happens, they are not well trained to resuscitate those patients. I've seen people die here because of that." – Medical officer

Receiving care
Poorly staffed facilities
In the district hospitals, there is a shortage of staff to provide care. The Casualty departments are largely run by clinical associates or junior doctors, who sometimes have difficulty getting senior supervision. Also, in the regional hospital, there is one orthopaedic surgeon and one general surgeon. In the tertiary hospital, there are more orthopaedic and general surgeons but only one neurosurgeon. When these experts are not available, care for the patients is severely delayed.

"From my side, as a clinical associate, some of the patients, you need a second opinion from a doctor or something like that, but it is almost impossible, if not impossible, to get that person whom you can talk to about the patient. So, that's the biggest challenge."—Clinical associate

Inadequately trained staff
An important recurring theme was the need for more training. Many HCPs did not receive standardized training in trauma care e.g. ATLS courses and those that did expressed the lack of continued learning. Not only are there barriers of poor staffing that limit the ability to explore training options located in other provinces, but there are also financial barriers, as HCPs said they financed the course and travel costs independently.

"There is no specific ATLS course given to us, actually in the province as a whole. I understand that in Guateng, and in Limpopo maybe. In other hospitals, they send their doctors, especially their junior doctors, to other places, to do their ATLS training, but with us, there is not that. So, you have to be organized and find out when an ATLS course is going to be, find out for yourself and pay for yourself. Which is not convenient also for the hospital … you cannot go away for a week … how many of us… let's say it's me and my colleague who decide to go. That means for that week, we will leave a hole

For peer review only

in the hospital, leave people struggling here, while you are out to educate yourself. Yes, so it is not the best."—Medical officer

"...now after you've stabilized your patient and there is another trauma case, you have to take your team with you, but someone has to stay and take care of the first patient and now you're faced with a situation where you have to give the most junior person the responsibility of watching the vitals, but they don't really know what they are watching and the next thing you know, the machine alarms go off ... they do not know what they are watching... they do not know that the [oxygen] saturation is not supposed to drop below 85% and then eventually you find yourself certifying a patient who ... (heavy sigh)... you see what I mean?"—Medical officer

Lack of equipment and supplies

Given financial constraints, there is often a lack of important medical equipment and supplies. When they are present, they easily become non-functional and are not repaired or replaced on time.

"...the second problem is life support equipment. Today it is there, tomorrow, it is broken down, yes ...there is a blood corner, where we keep emergency blood, maybe two or three units in a fridge."—Medical officer

Difficult referral system

A major problem in the care of severely injured patients, who are most commonly neurotrauma patients, is the ability to transfer them to a tertiary care institution, which is the only location for a computed tomography (CT) scan and access to a neurosurgeon. Some of the barriers include miscommunication about which hospitals receive referrals from the district hospitals and limited number of ambulances, especially ICU ambulances.

"the problem with serious trauma is the referral. When you have to refer to a higher institution, it's a hassle, it's a problem"—Clinical associate

"Ok, uhh, the first, the big barrier, is the referral system. I can have a patient here, head injury, low GCS, they bring the patient in respiratory distress. I'll try to stabilize the patient. I'll try to stop the bleeding, stabilize the patient, tube the patient, and then...I'll get the doctors at the higher level, they will accept the patient, but to transfer that patient, I need an ICU ambulance but in the province, there are only two ICU ambulances. So, the ICU ambulance probably has to leave Witbank to come here, maybe it can take three to four hours. Then take the patient from here to Nelspruit, that is six hours, then it is a problem...especially since we don't have good ventilation equipment."—Medical officer

"Well, sometimes, it's difficult because our referral system is contradictory. Like last week, we were surprised that our referrals should be going to Themba hospital. Meanwhile, we have been arranging them with Mapulaneng ...They said that memo had been written in March, but we didn't know about it. Now, when we communicate with Themba, like last night, the doctor on call was trying to arrange with Themba for

For peer review only

> *an acute abdomen…but they wanted him to send to Mapulaneng…it's still a bit of a confusion. It's difficult."*—Medical officer

Staff morale

Given the numerous challenges described above, the HCPs described feelings of frustration and disappointment with the amount of care they could provide, especially knowing that in better circumstances, they could save more lives.

> *"If you get an ICU ambulance, you have to get the intermediate trained ICU paramedics, or you have to get a chopper and that takes long so honestly, there is only so much you can do here. Because after you've given your drugs and you've intubated and ventilated, what more can you do? You're just sitting there and the CSF is just leaking and then 6 hours later, they come and you know, come on, you think, why are we even doing this? Then they go there, they do the CT scan and then they find that there is no neurosurgeon on call, so what do they do now? They just send the patient back. So, in terms of trauma, we still have a long way to go."*—Medical officer

For peer review only

STROBE Statement—Checklist of items that should be included in reports of ***cross-sectional studies***

	Item No	Recommendation	Page No
**Title and abstract**	1	(*a*) Indicate the study's design with a commonly used term in the title or the abstract	1
		(*b*) Provide in the abstract an informative and balanced summary of what was done and what was found	2
**Introduction**			
Background/rationale	2	Explain the scientific background and rationale for the investigation being reported	4
Objectives	3	State specific objectives, including any prespecified hypotheses	4
**Methods**			
Study design	4	Present key elements of study design early in the paper	4
Setting	5	Describe the setting, locations, and relevant dates, including periods of recruitment, exposure, follow-up, and data collection	5
Participants	6	(*a*) Give the eligibility criteria, and the sources and methods of selection of participants	5,6
Variables	7	Clearly define all outcomes, exposures, predictors, potential confounders, and effect modifiers. Give diagnostic criteria, if applicable	5,6
Data sources/ measurement	8*	For each variable of interest, give sources of data and details of methods of assessment (measurement). Describe comparability of assessment methods if there is more than one group	*5,6*
Bias	9	Describe any efforts to address potential sources of bias	5,6
Study size	10	Explain how the study size was arrived at	5,6
Quantitative variables	11	Explain how quantitative variables were handled in the analyses. If applicable, describe which groupings were chosen and why	6
Statistical methods	12	(*a*) Describe all statistical methods, including those used to control for confounding	6
		(*b*) Describe any methods used to examine subgroups and interactions	-
		(*c*) Explain how missing data were addressed	5
		(*d*) If applicable, describe analytical methods taking account of sampling strategy	6
		(*e*) Describe any sensitivity analyses	-
**Results**			
Participants	13*	(a) Report numbers of individuals at each stage of study—eg numbers potentially eligible, examined for eligibility, confirmed eligible, included in the study, completing follow-up, and analysed	8,13
		(b) Give reasons for non-participation at each stage	-
		(c) Consider use of a flow diagram	Fig1
Descriptive data	14*	(a) Give characteristics of study participants (eg demographic, clinical, social) and information on exposures and potential confounders	8-13
		(b) Indicate number of participants with missing data for each variable of interest	-
Outcome data	15*	Report numbers of outcome events or summary measures	8-12
Main results	16	(*a*) Give unadjusted estimates and, if applicable, confounder-adjusted estimates and their precision (eg, 95% confidence interval). Make clear which confounders were adjusted for and why they were included	-

For peer review only

For peer review only - http://bmjopen.bmj.com/site/about/guidelines.xhtml

		(*b*) Report category boundaries when continuous variables were categorized	-
		(*c*) If relevant, consider translating estimates of relative risk into absolute risk for a meaningful time period	-
Other analyses	17	Report other analyses done—eg analyses of subgroups and interactions, and sensitivity analyses	-
**Discussion**			
Key results	18	Summarise key results with reference to study objectives	15
Limitations	19	Discuss limitations of the study, taking into account sources of potential bias or imprecision. Discuss both direction and magnitude of any potential bias	17
Interpretation	20	Give a cautious overall interpretation of results considering objectives, limitations, multiplicity of analyses, results from similar studies, and other relevant evidence	15-16
Generalisability	21	Discuss the generalisability (external validity) of the study results	15,17
**Other information**			
Funding	22	Give the source of funding and the role of the funders for the present study and, if applicable, for the original study on which the present article is based	18

*Give information separately for exposed and unexposed groups.

Note: An Explanation and Elaboration article discusses each checklist item and gives methodological background and published examples of transparent reporting. The STROBE checklist is best used in conjunction with this article (freely available on the Web sites of PLoS Medicine at http://www.plosmedicine.org/, Annals of Internal Medicine at http://www.annals.org/, and Epidemiology at http://www.epidem.com/). Information on the STROBE Initiative is available at www.strobe-statement.org.

For peer review only

BMJ Open

EXTERNAL INJURIES, TRAUMA AND AVOIDABLE DEATHS IN AGINCOURT, SOUTH AFRICA: A RETROSPECTIVE OBSERVATIONAL AND QUALITATIVE STUDY

Journal:	*BMJ Open*
Manuscript ID	bmjopen-2018-027576.R2
Article Type:	Research
Date Submitted by the Author:	21-Apr-2019

Complete List of Authors:	EDEM, IDARA; UNIVERISITY OF OTTAWA, NEUROSURGERY<br>Dare, Anna; University of Toronto Faculty of Medicine, Department of Surgery<br>Byass, Peter; Umea Universitet, Umeå Centre for Global Health Research<br>D'Ambruoso, L; University of Aberdeen School of Medicine and Dentistry, Centre for Global Development and Institute of Applied Health Sciences<br>Kahn, Kathleen; University of the Witwatersrand School of Public Health<br>Leather, Andy; King's Health Partners and King's College London, King's Centre for Global Health<br>Tollman, Stephen; University of the Witwatersrand School of Public Health<br>Whitaker , John; King's Health Partners, King's Centre for Global Health<br>Davies, Justine ; King's College London, Centre for Global Health

**Primary Subject Heading**:	Global health
Secondary Subject Heading:	Emergency medicine, Public health, Qualitative research, Epidemiology
Keywords:	ACCIDENT & EMERGENCY MEDICINE, EPIDEMIOLOGY, Quality in health care < HEALTH SERVICES ADMINISTRATION & MANAGEMENT, PUBLIC HEALTH, TRAUMA MANAGEMENT

SCHOLARONE™
Manuscripts

External injuries, trauma and avoidable deaths in Agincourt, South Africa: A Retrospective Observational and Qualitative Study

Idara Edem – 1; Anna Dare – 2; Peter Byass – 3, 4; Lucia D'Ambruoso – 3, 5; Kathleen Kahn – 3; Andy Leather – 6; Stephen Tollman – 3; John Whitaker -6; Justine Davies – 7, 3.

Authors are in alphabetical order, apart from first, second, and last authors.

1. Division of Neurosurgery, Department of Surgery, University of Ottawa, Ottawa, Ontario, Canada
2. Centre for Global Health Research, St. Michael's Hospital and University of Toronto, Toronto, Ontario, Canada
3.MRC/Wits Rural Public Health and Health Transitions Research Unit—School of Public Health, Faculty of Health Sciences, University of the Witwatersrand, Johannesburg/Acornhoek, South Africa
4. WHO Collaborating Centre for Verbal Autopsy, Epidemiology & Global Health, Dept. of Public Health & Clinical Medicine, Umeå University, Umeå, Sweden
5. Centre for Global Development, University of Aberdeen
7. Institute for Applied Health Research, University of Birmingham, Birmingham, UK

Corresponding Author
Idara Edem
725 Parkdale Avenue, WM 150F, Mailbox 411
Ottawa, Ontario, Canada
K1Y4E9
iedem@toh.ca
613-282-8690

For peer review only

ABSTRACT

Objective: Injury burden is highest in Low and Middle Income Countries (LMICs). To reduce avoidable deaths, it is necessary to identify health system deficiencies preventing timely, quality care. We developed criteria to use Verbal Autopsy (VA) data to identify avoidable deaths and associated health system deficiencies.

Setting: Agincourt, rural Bushbuckridge municipality, Mpumalanga province, South Africa.

Participants: Agincourt Health and Socio-Demographic Surveillance System (HDSS) and Health Care Providers (HCPs) from local hospitals.

Methods: A literature review to explore definitions of avoidable deaths after trauma and barriers to access to care using the "three delays framework" (seeking, reaching, and receiving care) was performed. Based on these definitions, this study developed criteria, applicable for use with VA data, for identifying avoidable death and which of the three delays contributed to avoidable deaths. These criteria were then applied retrospectively to the VA-defined category External Injury Deaths (EIDs—a subset of which are trauma deaths) from 2012-2015. The findings were validated by external expert review. Key informant interviews (KIIs) with Health Care Providers (HCPs) were performed to further explore delays to care.

Results: Using VA data, avoidable death was defined with a focus on survivability, using level of consciousness at the scene and ability to seek care as indicators. Of 260 EIDs (189 trauma deaths), there were 104 (40%) avoidable EIDs and 78 (30%) avoidable trauma deaths (41% of trauma deaths). Delay in receiving care was the largest contributor to avoidable EIDs (61%) and trauma deaths (59%) followed by delay in seeking care (24% and 23%) and in reaching care (15% and 18%). KIIs revealed context-specific factors contributing to the third delay, including difficult referral systems.

Conclusions: A substantial proportion of EIDs and trauma deaths were avoidable, mainly occurring due to facility-based delays in care. Interventions, including strengthening referral networks, may substantially reduce trauma deaths.

Key words: Trauma, Avoidable death, Verbal autopsy, Rural, South Africa

For peer review only - http://bmjopen.bmj.com/site/about/guidelines.xhtml

ARTICLE SUMMARY: STRENGTHS AND LIMITATIONS OF THIS STUDY

- As far as we are aware, this is the first time that verbal autopsy data has been used to ascertain avoidable mortality and access to care after trauma.
- As well, it is the first time that the three delays framework for access to emergency medical care has been systematically applied to trauma and avoidable mortality, allowing for the quantification of the delays to trauma care.
- Although this study was performed in a single Health and Socio-Demographic Surveillance System (HDSS), the findings are novel, pertinent to the Sustainable Development Goals and the methodology can be more widely applied to other VA data worldwide.
- Key informant interviews of health care workers added useful information to the VA data, but this information is likely to be skewed towards healthcare facilities.
- The VA methodology involves interviews conducted by well-trained but non-medical personnel, and does not capture detailed vitals at the scene or the nature of pre-hospital and in-hospital emergency care received, which can result in missing important clinical aspects of the trauma and the care sought.

For peer review only

For peer review only - http://bmjopen.bmj.com/site/about/guidelines.xhtml

INTRODUCTION

Injuries account for an estimated 5.8 million deaths per year, 32% more than HIV, malaria and TB, combined.[1] The burden of trauma deaths is largest in Low and Middle Income Countries (LMICs) which are also the most ill-equipped to manage this burden.[2] Although there has been a global drive for injury prevention and we are in the "decade of action for road safety", morbidity and mortality from injuries continues to rise.[3] Alongside injury prevention efforts, optimization of medical care provided after injury occurrence is important. In many LMICs, there are deficiencies in Emergency Medical Services (EMS) and definitive hospital care for the injured. Where present, these are hampered by limited geographical coverage, resources, and trained staff.[4]

In order to improve a health system's ability to manage a large burden of injuries, gaps in care provision must be identified to guide resource allocation. To assist with this, the concept of completely or partially avoidable deaths—given the provision of effective health care—can be used[5] in association with exploration of the factors which led to an avoidable death occurring.

The three-delays framework (delays in seeking, reaching and receiving care) for accessing safe, affordable, and timely medical care is regarded as a classic conceptualization of delayed care in emergency situations, reflected in its application in obstetric emergencies,[6] sepsis management,[7] perinatal care[8] and hip fractures.[9] To the best of our knowledge, the three-delays model has not previously been analytically applied to trauma, but given the similarities between trauma and other time-critical conditions in which it has been used,[6] it is an appropriate model for investigating gaps in the health system contributing to avoidable trauma deaths.

To explore avoidable trauma deaths, a complete understanding of deaths and the events surrounding these deaths is needed. A lack of Civil Registration and Vital Statistics (CRVS) systems in many LMICs,[10] coupled with limited physician-assigned cause of death documentation, means that the verbal autopsy (VA) has become a practical tool for ascertaining cause of death, including for injuries.[11] VAs have not previously been used to examine avoidable deaths in trauma.

Our aims were: 1) To develop and validate criteria applicable to VA data to estimate numbers of avoidable deaths in trauma; 2) In cases where deaths were avoidable, to develop criteria for assessing where in a three delays framework delays leading to deaths occurred, and estimate the numbers of cases where such delays were experienced; 3) Perform qualitative interviews with HCWs to further explore delays to care; 4) To combine information from the literature, VA, and qualitative analysis to construct a three delay's framework applicable to avoidable mortality in trauma.

For peer review only - http://bmjopen.bmj.com/site/about/guidelines.xhtml

METHODS

Literature review

To determine definitions and criteria for avoidable mortality and factors contributing to the three delays in trauma, PubMed, Ovid and EMBASE were searched using the terms: “trauma” AND “avoidable death” OR “preventable death” OR “mortality” OR “delays” and limited to papers published in English, from 1990 onwards, in HICs and LMICs. The titles and abstracts were reviewed to select papers addressing avoidable death in trauma and delays to care.

Setting

The study was performed in Agincourt, located in the rural Bushbuckridge municipality of Mpumalanga province, South Africa. A Health and Socio-Demographic Surveillance System (HDSS) has regularly collected household data on health and vital events (births, deaths, migrations) for over 115,000 people in a geographically defined area since 1992. All deaths identified during surveys are followed up and a VA is conducted within one year of the death.

Verbal Autopsy

VA involves interview of the lay-primary caregiver of the deceased by a trained fieldworker about signs, symptoms and circumstances surrounding death. The interviews are completed within 12 months of the death, using a validated questionnaire, with good previously demonstrated recall.[12] Currently, a computer algorithm, InterVA-4, is utilized to determine cause of death, using standardized probabilistic models.[13]

VA data used in this study was collected using the WHO 2012 short-form. [14] In addition to standard medical questions to allow cause of death to be ascertained, this contains a *trauma module*—41 questions to further query circumstances surrounding trauma deaths—and 10 “circumstances of mortality” (COM) questions that address the “household, community and health systems determinants of health” [15] influencing mortality. In the trauma module, for each category of death, there are further specific questions to pinpoint the exact details of the deaths. COM questions are grouped under 4 themes—recognition of severity, mobilizing assets to seek care, access to care, and quality of care. Additionally, VAs contain a free text summary of the circumstances surrounding deaths, obtained from the respondent, that allows information not captured in the survey questions to be ascertained.

Data from all VAs between 2012 and 2015 categorized as “external injury deaths” (EIDs)[1] by InterVA-4 were extracted.[13] InterVA-4 classifies the following as EIDs: poisonings, drownings, deaths from natural disasters and “traumas”—which, as a subclass of EIDs, include road traffic accidents, assaults, fires and falls. This broad VA category of external injury deaths, of which trauma is a subset, are referred to in the text as external injury deaths, EIDs. Additionally, free-text searches of the open free-text summary portion of the VAs were performed to identify

[1] EIDs correspond to external causes of death in chapter 12 of the WHO 2012 VA instrument (WHO, 2012).

For peer review only - http://bmjopen.bmj.com/site/about/guidelines.xhtml

trauma deaths that may have been incorrectly categorized. Search terms included: injury, trauma, fall, accident, traffic and fire.

Defining avoidable death and developing a three-delays framework

Findings from the literature review informed which variables contained in the VA questions and factors extractable from the free text had utility for defining avoidable deaths and in the creation of a three-delays framework. In particular, each narrative in the free text was reviewed for details pertaining to location of external injury or death, acuity or chronicity of external injury, signs and symptoms after external injury, ability to seek care, factors facilitating or inhibiting access to care, the provision of medical care, results of care delivered, and understanding of the effects of care on the patient's outcome.

VA reports on deaths from 2015 were first utilized to categorise deaths by avoidability and delay. The categorisations of avoidability and three delays in a subsample of 20 cases were then validated by external review with a researcher and trauma care provider (JW) familiar with the geographic context. Discrepancies were discussed until consensus was achieved in order to improve classification of subsequent data. Once agreement was reached on categorizing deaths by avoidability and then using the three delays framework, avoidable deaths and delays were discerned for all EIDs from 2012 to 2014. Where there were multiple delays contributing to the avoidable mortality, that which was found to be the most immediate factor was considered the primary contributing delay.

Qualitative analysis

Purposive sampling was utilized to identify emergency-care providers from local hospitals to participate in Key Informant Interviews (KIIs) and snowball sampling was used until responses reached saturation and no new ideas were revealed.[16] The interviews were face-to-face semi-structured and open-ended. They were completed in English, recorded on tape, transcribed, and transferred for analysis into NVivo version 11.4 (QSR International, 2017). Transcripts were analysed using framework methods [17] within a thematic analysis. This allowed for unanticipated meanings to emerge from the data and analysis, as well as for the data to be structured based on the overall aims and objectives of the study, thus permitting both inductive and deductive data analyses.[18] This was an iterative process, to ensure that the content of the narrative data was as fully represented as possible. Identified factors were then compared with those garnered from the literature review and the VA analysis.

Final three delays framework

The findings from the literature review, VA, and qualitative analyses were combined to construct a holistic three delays framework for assessing avoidable deaths in trauma.

Statistical considerations

This is an exploratory study and a power calculation was not performed. Data are described. Analysis was performed using SPSS Statistics software (IBM, version 24), NVivo version 11.4 (QSR International, 2017) and Microsoft Excel.

For peer review only - http://bmjopen.bmj.com/site/about/guidelines.xhtml

Ethics approval and consent
Ethics approval from the University of the Witwatersrand Human Research Ethics Committee to use data from the VAs in the Agincourt HDSS in secondary analyses had been previously obtained (M960720 and M110138). All analysed VA data was anonymized and no individual deceased person or respondent was identifiable from the data used and presented.

Ethics approval for the independent KIIs was obtained from the University of the Witwatersrand Human Research Ethics Committee (M170269) and King's College London Research Ethics Committee (LRU-16/17-4313). Participation in the qualitative study was voluntary and informed consent was obtained for the interview and its recording. All interviews were confidential and narratives were anonymized.

Patient and Public Involvement Statement

Patients were not directly involved in this exploratory study and no new patient data was collected. However, although no patients were involved in this study, Agincourt employs community engagement officers to ensure that community members are aware of research being done in the area – even if community dwellers are not involved – and that results are fed-back to the community.

RESULTS
Literature review
Fifty-seven papers which explored the conceptualisations of avoidable death in trauma and delays in access to care were identified, as were nine additional papers from review of references sections. Of these, 43 papers were included in the literature review. Despite differences in the definition of avoidable death in trauma, common themes included: external injury survivability, deviations from delivery of optimal care and implication of errors in care delivery on the death of the patient. These themes are also reflected in the WHO guideline for the definition of avoidable death in trauma—note that this guideline definition also includes calculations of the probability of survival and injury severity scores.[2] Definitions of survivability which do not use injury severity scores were based on vital signs at the scene; [19]being awake at the scene; [19-21]or being able to seek care after external injury; or having reversible, stable,[22] or non-severe external injuries involving a single-system.[23,21,19,20] Most studies used a combination of these definitions (see appendix 1 for details on approaches to determine criteria for survivability).

Relevant components of the three delays model identified in the literature were as follows: deciding to seek care (socio-economic and cultural factors, perceived accessibility and perceived quality of care e.g. perceived severity, transportation, costs, EMS transport protocols, reputation of previous experience); identifying and reaching care (actual accessibility factors e.g. EMS accessibility and timeliness of response, prehospital care); receiving adequate and appropriate treatment (actual quality of care factors e.g. poorly staffed facilities and

For peer review only - http://bmjopen.bmj.com/site/about/guidelines.xhtml

inadequate management) (Appendices 1 and 2). See Appendix 3 for the full list of articles included in the literature review.

VA Data

Figure 1 shows VA numbers included in the study. Between 2012 and 2015, there were 3001 deaths; of these 260 deaths were categorized as EIDs (8.7% of total) of which 189 were due to trauma (Table 1). The sub-category distribution for both EIDs and trauma deaths are shown in Appendix 4. Traffic and assault injuries were the most common causes of trauma deaths.

Table 1. Demographic characteristics of the EID and trauma (a subset of EID) groups, for all, avoidable, and non-avoidable deaths from 2012-2015

Category	EIDs	Avoidable EID	Non-avoidable EID	Trauma deaths	Avoidable trauma deaths	Non-avoidable trauma deaths
Total	**260**	**104**	**156**	**189**	**78**	**111**
**Age group**						
Older (>65 y)	19 (7.3%)	12 (11%)	7 (4.5%)	12 (6%)	9 (11%)	3(2.7%)
Mid-age (50-64 y)	27 (10%)	10 (9.6%)	17 (11%)	20 (11%)	8 (10%)	12(11%)
Adult (15-49 y)	188 (72%)	74 (71%)	114 (73%)	143 (76%)	57 (73%)	86 (78%)
Child (5-14 y)	14 (5.4%)	4 (3.8%)	10 (6.4%)	7 (3.7%)	1 (1.3%)	6(5.4%)
Under-5 (1-4 y)	10 (3.8%)	3 (2.9%)	7 (4.5%)	5 (2.6%)	2 (2.6%)	3(2.7%)
Infant (1-11 months.)	1 (0.4%)	0	1 (0.6%)	1 (0.5%)	0	1(0.9%)
Neonate (<28 days)	1 (0.4%)	1 (1.0%)	0	1 (0.5%)	1 (1.3%)	0
**Sex**						
Male	198 (76%)	75 (72%)	123 (79%)	147 (78%)	59 (76%)	88(79%)
Female	62 (24%)	29 (28%)	33 (21%)	42 (22%)	19 (24%)	23(21%)

Development of avoidable death and three delays criteria for use with VA

In 2015, out of 482 total deaths, there were 48 EID cases which were used to develop criteria for determining avoidability. Descriptive information on survivability were the only criteria available. Factors indicating survivability were: being alive at the scene, being able to seek help, or non-severe single-system injuries (Table 2). Twenty (42%) EIDs were determined to be avoidable (Table 1). The external reviewer agreed on classification of avoidability of death in 90% of cases.

For peer review only - http://bmjopen.bmj.com/site/about/guidelines.xhtml

From this 2015 VA data, there were several extractable factors relevant to each of the three delays (Table 2). There was 80% agreement with the external expert for classification of delays contributing to death. Further analysis and discussion of VA free-text data was performed together, until consensus was reached and a three delay's framework using data relevant to VA was constructed. Data relevant only to the circumstances of mortality module are contained in Appendix 5.

Table 2. Details on the data sections of VAs used to define injury survivability and determine delays to care

DESCRIPTION	VA SECTIONS CONTAINING RELEVANT DATA	VARIABLES EXTRACTED
Injury survivability	Demographics	Age
	Acuity of death	Acute versus chronic injury/death
	Previous medical history	List of medical conditions
	Signs and symptoms	System-based list of signs and symptoms
	External injury classification	Category of injury
	Trauma module	Details of injury classification and mechanism of injury
	Free-text disease descriptions	Vitals at the scene<br>Injured body system<br>Signs and symptoms at the scene and at hospital presentation<br>Injury severity<br>Actions taken immediately after injury<br>Ability to seek care
First delay	Circumstances of mortality questions	Doubts about the need for care<br>Use of traditional medicine<br>Did not use a mobile phone to call for help
	Free-text disease descriptions	Healthcare literacy and perceived severity of the injury

For peer review only - http://bmjopen.bmj.com/site/about/guidelines.xhtml

		Perceived costs of seeking care—transport, medical costs<br>Perceived quality of care<br>Previous experiences with medical care
	Trauma module	Traffic accident location i.e. rural versus urban<br>Death at site or time of injury
Second delay	Circumstances of mortality questions	Did not use otorized transport to get to care<br>>2 hrs travel to care<br>Prohibitive costs<br>Did not travel to a hospital or health facility
	Free-text disease descriptions	EMS accessibility<br>EMS timeliness of response<br>EMS versus personal transport<br>Prehospital care
Third delay	Circumstances of mortality questions	Problems with admission<br>Problems with treatment (medical treatment, procedures, interpersonal attitudes, respect, dignity)<br>Problems with tests and medications
	Free-text disease descriptions	Inadequate and/or delayed diagnosis and/or treatment<br>Delayed inter-hospital transfers<br>Poorly staffed and/or equipped facilities
	Treatment and health care utilization	Medical and/or surgical treatment<br>Traditional medicine treatment

For peer review only - http://bmjopen.bmj.com/site/about/guidelines.xhtml

		If medical and traditional medicine treatment sought, which was sought first?<br>Medical aid or employer support for healthcare<br>Medical actions<br>If no medical actions taken, reasons for not taking medical action

For peer review only

Avoidable mortality and three delays framework for EIDs and trauma, 2012-2015
Applying the definition of avoidable deaths to the 2012-2015 VA data revealed 104 avoidable EIDs (40%) and 78 avoidable trauma deaths (41%) (Table 1, Appendix 4).

Based on the three delays framework, 58% of people with avoidable EIDs experienced one delay, 38% two delays, and 4% three delays. For avoidable trauma deaths, 57% experienced one delay, 38% two delays, and 5% three delays. For all avoidable deaths, perceived external injury severity, deficiencies in prehospital care, and inadequate and/or delayed diagnosis and/or treatments were the primary contributing factors in delays 1, 2 and 3, respectively (Table 3). Delay three was the most commonly occurring contributing delay to avoidable deaths, in 61% and 59% of EIDs and trauma deaths, respectively.

Table 3. Primary contributing factors in each delay category for avoidable EID and trauma deaths, 2012-2015.*

**Delay**	**Number (%)**	**Primary contributing delay factors**	**Number (%)**
**Avoidable EIDs (n=109)**			
1—Seeking care	25 (24%)	Perceived severity<br><br>Perceived etiology	35 (74%)<br><br>6 (12.7%)
2—Reaching care	16 (15%)	Prehospital care<br><br>EMS timeliness and response	28 (61%)<br><br>17 (37%)
3—Receiving care	63 (61%)	Inadequate and/or delayed diagnosis and/or treatment<br><br>Delayed inter-hospital transfer	82 (78%)<br><br>11 (10%)
**Avoidable trauma deaths (n=81)**			
1—Seeking care	18 (23%)	Perceived severity	23 (77%)

For peer review only - http://bmjopen.bmj.com/site/about/guidelines.xhtml

		Delayed discovery	3 (9.6%)
2—Reaching care	14 (18%)	Prehospital care<br><br>EMS timeliness and response	25 (63%)<br><br>13 (34%)
3—Receiving care	46 (59%)	Inadequate and/or delayed diagnosis and/or treatment<br><br>Delayed inter-hospital transfer	59 (77%)<br><br>9 (11%)

*Note that one individual may experience more than one delay.

When considering just the circumstances of mortality indicators (appendix 5), these captured minimal data about the quality of care. Also, there were no cases where respondents reported having "greater than 2 hours' travel to care" or "doubts about the need for care". However, a mobile phone was not used to call for help in 26% of EID cases and 28% did not travel to a hospital for care. For avoidable trauma deaths, 27% did not use a phone to call for help and 27% did not travel to a hospital for care. Also, issues with the prohibitive costs of seeking care were present in 20% of the avoidable EIDs and 21% of the avoidable trauma deaths.

KIIs and qualitative analysis

Seven KII were needed to reach saturation; these were done with two clinical associates (three-year training for a Bachelor of Clinical Medical Practice), four medical officers (four-year medical degree, two-year internship, one-year community service), and one Casualty (ER) manager working in the three hospitals in Bushbuckridge: Matikwane, Tintswalo and Mapulaneng. Important themes included healthcare literacy, EMS versus personal transport, mistrust of clinics (delay 1); EMS accessibility and timelines, prehospital care (delay 2); poorly staffed facilities, inadequately trained staff, lack of equipment and supplies, difficult referral systems and staff morale (delay 3). See below and appendix 6 for full details on these themes). [2]

Seeking care

Health or healthcare literacy

KIs felt that most patients were unaware of proper channels for seeking care, especially when an injury was not acutely severe.

> *"So, there are those who say straight to the hospital and those who say no but you can never actually tell which one because they don't know themselves… it's just a matter of their specific attitude, and there are mistakes in both ways …"*—Medical officer

[2] Note that an abridged version of the KII data analysis is presented here, but more details are presented in Appendix 6.

For peer review only - http://bmjopen.bmj.com/site/about/guidelines.xhtml

EMS vs. personal transport

KIs recounted that patients are aware of delayed responses from EMS and previous experience with EMS delays may affect the decision to seek care.

> *"From what we've heard, it's delayed response from the ambulances, the emergency services …it's a problem… and also for the patients, I think because this place is surrounded by villages…if they don't have transport, their own transport to come here, it becomes a bit of a challenge"* –Clinical associate

Reaching care

EMS accessibility and timeliness

The KIs felt that the largest barrier for patients reaching care was EMS accessibility. Given the rural area and the limited number of ambulances, several descriptions were provided of patients waiting for long periods to be taken to hospital.

> *"… the biggest problem I think they have is access to emergency care personnel, the paramedics, the ambulance. People may have trauma cases and when they call, the ambulance is already out, attending to trauma care somewhere else. It might take two to three hours for this ambulance to come and pick this patient. Now that is just too long for some cases. That might mean the difference between being normal again and being invalid or even dead."*—Medical officer

Prehospital care

As well as the issues with EMS timeliness, the care provided by EMS providers was described as limited by the training received, as well as by lack of equipment to provide early resuscitation.

> *"It's not only death, we can also prevent the sequelae people come in with…they get brain damage, because the care was too late. The thing of the transport is very important, because the other guys, the EMS, if something happens, they are not well trained to resuscitate those patients. I've seen people die here because of that."* – Medical officer

Receiving care

Inadequately trained staff

A key recurring theme was the need for more training. Many KIs reported that they did not receive standardized training in trauma care (e.g. Advanced Trauma Life Support [ATLS] courses) and those that did expressed the lack of continued learning.

> *"There is no specific ATLS course given to us, actually in the province as a whole…In other hospitals, they send their doctors, especially their junior doctors, to other places, to do their ATLS training, but with us, there is not that. So, you have to be organized and find out when an ATLS course is going to be, find out for yourself and pay for yourself. Which is not convenient also for the hospital … you cannot go away for a week…let's say it's me*

For peer review only - http://bmjopen.bmj.com/site/about/guidelines.xhtml

> *and my colleague who decide to go. That means for that week, we will leave a hole in the hospital."*—Medical officer

Lack of equipment and supplies

KIs reported that given financial constraints, there is often a lack of important medical supplies and when they are present, they easily become non-functional without being repaired or replaced on time.

> *"…the second problem is life support equipment. Today it is there, tomorrow, it is broken down, yes …there is a blood corner, where we keep emergency blood, maybe two or three units in a fridge."*—Medical officer

Difficult referral system

A major problem in the care of severely injured patients, who are most commonly neurotrauma patients, is the ability to transfer them to a tertiary care institution, which is the only location for a computed tomography (CT) scan and access to a neurosurgeon. KIs recounted that some of the barriers include miscommunication about where to transfer patients to and limited ICU ambulances.

> *"Ok, the first, the big barrier, is the referral system. I can have a patient here, head injury, low GCS [Glasgow Coma Scale], they bring the patient in respiratory distress. I'll try to stabilize the patient…tube the patient…I'll get the doctors at the higher level, they will accept the patient, but to transfer that patient, I need an ICU ambulance but in the province, there are only two ICU ambulances…maybe it can take three to four hours…Then take the patient from here to [the other hospital], that is six hours, then it is a problem…especially since we don't have good ventilation equipment."*—Medical officer

> *"Well, sometimes, it's difficult because our referral system is contradictory. Like last week, we were surprised that our referrals should be going to [this] hospital. Meanwhile, we have been arranging them with [another hospital] …They said that memo had been written in March, but we didn't know about it."*—Medical officer

Composite three-delays framework for avoidable deaths in trauma

The creation of the final three-delays framework for avoidable death in trauma based on analysis of information from three methodologies (literature review, VA analysis, and KIIs) is shown in Figure 2.

DISCUSSION

From 2012 to 2015, a large proportion of VA-defined external injury deaths were avoidable. Most avoidable EIDs occurred in male adults between 15-49 years old and were a result of traffic injury or assault. A delay in receiving definitive care (the 'third delay') was the largest contributing factor in avoidable deaths, and occurred as a result of delays in diagnosis and

For peer review only - http://bmjopen.bmj.com/site/about/guidelines.xhtml

treatment, inadequate referral systems for injured patients and poorly staffed and equipped health facilities, particularly at the district hospital level. These findings demonstrate that VA data examining the nature of the death and circumstances around death can be used to determine avoidable deaths and explore the factors contributing to these. Qualitative information from local health care providers provided useful additional information.

Globally, there is an increasing prevalence of trauma morbidity and mortality, especially in people younger than 45 years old,[24] with road traffic injuries being the second leading cause of death in 15-49 years old and the first in males.[25,26] In Mpumalanga, road traffic injuries were the second leading cause of years of life lost in 2012, after HIV/AIDS.[27] Trauma burden is worse in LMICs, given the limited scope of trauma systems and the challenges of service delivery in already strained health systems.[4] When injuries do occur, it is important to have trauma systems in place that ensure that the injured person receives care from the time of injury occurrence, to reduce avoidable mortality. The logical way to develop such systems in countries with nascent health care systems is to first identify gaps in current services. To our knowledge, this is the first time that data from VAs has been used to do this. Findings from this study should contribute to future local health system planning and create a path for future studies at other HDSSs collecting VA data.

Avoidable mortality was identified using data available from VA with criteria informed by a review of the literature. In trauma, there is heterogeneity in methods for categorizing avoidable deaths, but survivability—defined using injury severity scores and expert panels—is the commonest methodology.[28] Although survivability scores were not available in this study, VA contained data which were used – with good agreement – to define survivability. It is reassuring that identified themes here are also reflected in the WHO guideline for the definition of avoidable death in trauma. The results on rates of avoidability are also similar to findings from other LMICs.[4,29-31] However, the avoidable death rate found in this study is greater than that found in high income countries.[28,32-34] The findings that most avoidable deaths were in the group defined by VA as adults (between 15 and 49 years old) and in males are also consistent with previous studies on trauma deaths.[21,35,24]

In this study, delays in receiving care could not be ascertained from VA review alone and KIIs provided useful additional data. HCPs revealed that factors including difficult referral systems, poorly staffed facilities, inadequately trained staff, and lack of equipment and supplies, all contributed to delayed or inadequate diagnosis and/or treatment, and thus avoidable death. These factors have also been shown to be contributors to avoidable trauma deaths in other studies [36-39] and they represent possible intervention points. Potentially, the widespread introduction of trauma training, including ATLS and training provided to HCPs in LMICs by institutions like Primary Trauma Care Foundation, could lead to reduced trauma avoidable mortality, as has been seen in other studies.[40] A review of the interventions to improve trauma care quality in LMICs, their cost-effectiveness and possible application in this setting, may be a worthwhile future study to pursue. Although the delays are presented linearly to enable understanding, they are actually interconnected, as delays in reaching and receiving care at one instance will influence the decision to seek care in future instances. Therefore, what

For peer review only - http://bmjopen.bmj.com/site/about/guidelines.xhtml

is determined to be the most immediate factor contributing to death and thus the largest contributing delay, may actually be the outcome of the effect of other delays in the past. As well, patients cycle through the delays model as their care plans change and they require transfer to other facilities, such that these patients face new and compounding delays as their care provision progresses.

The result that a substantial proportion of mortality is avoidable seems at odds with the findings in a recent study showing that, in South Africa, only 5.2% of the population is estimated to be outside a 2-hour travel time to emergency care.[41] These results are reflected in the analysis of the COM data where there were no reports of being outside a 2-hour travel distance to care. Yet, on examination of other parameters, including the free text, delays in reaching care – especially *appropriate* care – did contribute to avoidable mortality. Indeed, avoidable mortality may be best reduced by introducing effective prehospital trauma care [36] and by ensuring that a bypass protocol is implemented,[42] so that patients with severe trauma injuries are transported immediately to secondary or tertiary hospitals without first being delayed in district facilities. Many severely injured patients in this study experienced a substantial delay when sent first to district hospitals before awaiting transfer to regional or tertiary hospitals. This was corroborated by information from VA narratives and KIIs with emergency care providers. The goal of a well-functioning trauma system should be that the "right patient is treated at the right hospital at the right time".[43] So, if patients are severely injured, they are taken directly to hospitals with appropriate specialists. This has the advantage of eliminating inter-hospital transfer delays. However, the success of a trauma system with targeted patient transfers also depends on an appropriately equipped and staffed prehospital care system, which is lacking in many LMICs, as shown here.

Neurotrauma was a significant cause of avoidable trauma deaths in this study, as seen both from findings of the KIIs and VA review, where 35% of the avoidable trauma deaths had neurotrauma. This finding is likely an underestimation, given the lack of detailed medical information. Other studies have also found that most of the avoidable deaths in trauma are due to neurotrauma,[44,19] usually related to poor airway management.[45,24] In Bushbuckridge, where access to a neurosurgeon occurs only in a tertiary hospital outside of the municipality, it is paramount to provide adequate prehospital care, including proper airway management, as well as reduce inter-hospital transfer delays.

This study had several limitations. VA data does not capture detailed vitals at the scene, exact pre-hospital delays, or the nature of pre-hospital and in-hospital emergency care received. Therefore, although non-severe single-system injuries was used as a criteria for avoidable deaths, many more deaths could have occurred due to missed major secondary injuries, information that may not be well captured in VA data. Furthermore, the circumstances of mortality indicators provided information for the delays, but they did not capture important information about delays in reaching appropriate or quality care. The use of these indicators in South Africa was analysed in the census rounds of 2012-2013 and the researchers found that problems with not calling for help or not going to a medical facility were more pronounced than problems with overall costs in the younger age groups and in those with acute conditions, as in

For peer review only - http://bmjopen.bmj.com/site/about/guidelines.xhtml

most trauma cases.[15] In this study, KIIs added useful information to the VA data, but this information is likely to be skewed towards healthcare facilities. If other service providers or users were interviewed, further information about delays to access in care which are not contained in VA data may have been gleaned. An additional limitation is that the acute nature of trauma may also bias caregivers to report the care provided in hospital in more detail, as was seen in the free-text, so that if not asked specifically about the pre-hospital delays, these are not recollected or recorded. The VA methodology involves interviews conducted by well-trained but non-medical personnel, which can result in important clinical aspects of the injury and the care sought being missed. Since the VA interviews are conducted months after the death of the individual, recall bias becomes a major factor, affecting the reliability and validity of the information provided. Yet, although the VA is not the ideal tool for information on death and causes of death, it is a pragmatic method that provides useful information and its use continues to widen with greater innovations for its application.[15]

CONCLUSION

In this study, VA was shown to be a feasible method for defining avoidable deaths in trauma and ascertaining which of the three delays contribute to death. Between 2012 and 2015, a large percentage of VA-defined external injury (40%) or trauma (41%) deaths were avoidable. The third delay in the three-delays model was found to be the largest contributor to avoidable deaths and the qualitative study supported the findings from the VA. To combat the burden of avoidable trauma deaths in LMICs, there is a need for a functioning trauma system, with adequate health systems interventions in trauma prevention, access to medical care, EMS and adequately staffed and supplied hospitals. “When it comes to trauma care, where people live ought not to determine if they live”.[46]

FIGURE LEGENDS

Figure 1. Deaths considered in this study (EIDs and trauma), relative to total deaths in the HDSS

Figure 2. Composite three-delays framework for avoidable death in trauma.

*The most frequently cited factors from the VA analysis and KIIs analysis are shaded in blue.

ACKNOWLEDGMENTS

We thank Chodziwadziwa Kabudula (MRC/Wits Rural Public Health and Health Transitions Research Unit—School of Public Health, Faculty of Health Sciences, University of the Witwatersrand, Johannesburg/Acornhoek, South Africa) for his assistance with assembling the Agincourt HDSS dataset for our use.

This work has been previously presented at the following conferences: AANS 2018, New

For peer review only - http://bmjopen.bmj.com/site/about/guidelines.xhtml

Orleans, USA and CNS Bethune Round Table 2018, Toronto, Canada.

FUNDING

Funding for part of this project (travel scholarship) was provided by the King's College London, Centre for Global Health. This funding source was not involved in the study design; in the collection, analysis and interpretation of the data; in the writing of the manuscript; and in the decision to submit the paper for publication. Otherwise, this research received no specific grant from an agency of the public, commercial or not-for-profit sectors.

COMPETING INTERESTS

The authors do not report any competing interests or conflicts of interest.

CONTRIBUTIONS

IE helped to develop the idea, did the analysis, drafted and revised the paper; AD helped to develop the idea, and inputted into the paper drafts; PB inputted into the idea and the paper drafts; LD inputted into the idea, supported the analyses, and inputted into the paper drafts; AL, KK, and ST inputted into the idea and the paper drafts; KK set up and is overall responsible for the system of verbal autopsies; ST set up and is overall responsible for the Agincourt HDSS; JW contributed to the analyses and inputted into the paper drafts; JD led the project, developed the idea, supported the analyses, and inputted into the paper drafts; all authors approve the final paper for submission.

DATA SHARING

The data used for the VA analysis is available from the INDEPTH Data Repository Platform at: http://www.indepth-ishare.org/index.php/home
More data is available by emailing: iedem@toh.ca

For peer review only

REFERENCES

1. Lozano R, Naghavi M, Foreman K et al. Global and regional mortality from 235 causes of death for 20 age groups in 1990 and 2010: A systematic analysis for the Global Burden of Disease Study 2010. *Lancet* 2012;380:2095–128 doi: 10.1016/S0140-6736(12)61728-0

2. World Health Organization. Guidelines for Trauma Quality Improvement Programmes. Edited by C. Mock, C. Juillard, S. Brunage, J. Goosen, and M. Joshipura. Geneva: World Health Organization. 2009 doi: 10.1016/j.puhe.2010.01.011.

3. World Health Organization. *Saving Millions of lives: Decade of action for road safety 2011-2020*, *WHO publication*. Available at: http://who.int/violence_injury_prevention/publications/road_traffic/saving_millions_lives_en.pdf. Accessed March 2017.

4. Yeboah D, Mock C, Karikari P et al. Minimizing preventable trauma deaths in a limited-resource setting: A test-case of a multidisciplinary panel review approach at the Komfo Anokye Teaching Hospital in Ghana. *World J Surg* 2014;38:1707-12. doi: 10.1007/s00268-014-2452-z.

5. Rutstein D, Berenberg W, Chalmers T et al. Measuring the quality of medical care. A clinical method. *N Engl J Med* 1976;294:582-88.

6. Thaddeus S and Maine D. Too Far To Walk: Maternal Mortality in Context. *Soc Sci Med* 1994;38:1091–110.

7. Papali A, McCurdy M, Calvello E. A “three delays” model for severe sepsis in resource- limited countries. *J Crit Care* 2015;30:e9-14 doi: 10.1016/j.jcrc.2015.04.003.

8. Musafili A, Persson L, Baribwira C. et al. Case review of perinatal deaths at hospitals in Kigali, Rwanda: Perinatal audit with application of a three-delays analysis. *BMC Pregnancy and Childbirth* 2017;17:1–13. doi: 10.1186/s12884-017-1269-9.

9. Tewari A, Rath S, Yadav L et al. Care Seeking Behaviour of Older Adults with Hip Fracture in India: A Qualitative Study. *J Pat Care* 2017;3:128. doi: 10.4172/2573-4598.1000128.

10. Mikkelsen L, Phillips D, AbouZahr C et al. A global assessment of civil registration and vital statistics systems: monitoring data quality and progress. *Lancet* 2015;6736: 60171– 4.

11. Byass, P. Minimally Invasive Autopsy: A New Paradigm for Understanding Global Health? *PLoS Medicine* 2016;13: 8–11. doi: 10.1371/journal.pmed.1002173.

12. Kahn K, Collinson M, Gomez-Olive F et al. Profile: Agincourt health and socio- demographic surveillance system. *Int J Epidemiol* 2012;41:988–1001.

For peer review only - http://bmjopen.bmj.com/site/about/guidelines.xhtml

13. Murray CJ, Lozano R, Flaxman AD et al. Robust metrics for assessing the performance of different verbal autopsy cause assignment methods in validation studies. *Popul Health Metr* 2011;9:28.

14. World Health Organization. Verbal autopsy standards: The 2012 WHO verbal autopsy instrument. Geneva. Available at: http://www.who.int/healthinfo/statistics/verbalautopsystandards/en/index2.html. Accessed January 2017.

15. D'Ambruoso L, Kahn K, Wager RG et al. Moving from medical to health systems classifications of deaths: extending verbal autopsy to collect information on the circumstances of mortality. *Glob Health Res Policy*. *Global Health Research and Policy* 2016;1:2. doi: 10.1186/s41256-016-0002-y.

16. Ritchie J, Lewis J. Qualitative research practice: A guide for social science students and researchers. Los Angeles: Sage Publishing Ltd 2014.

17. Ritchie J, Lewis J. Qualitative research practice: a guide for social science students and researchers. London: Sage Publishing Ltd 2003.

18. Gale N, Heath G, Cameron E et al. Using the framework method for the analysis of qualitative data in multi-disciplinary health research. *BMC Medical Research Methodology* 2013;13:117. doi: 10.1186/1471-2288-13-117.

19. MacLeod JBA, Cohn SM, Johnson EW et al. Trauma deaths in the first hour: are they all unsalvageable injuries? *Am J Surg* 2007;193:195–9. doi: 10.1016/j.amjsurg.2006.09.010.

20. Kleber C, Giesecke MT, Tsokos M et al. Trauma-related preventable deaths in Berlin 2010: Need to change prehospital management strategies and trauma management education. *World J Surg* 2013;37: 1154–61. doi: 10.1007/s00268-013-1964-2.

21. Ray JJ, Meizoso JP, Satahoo SS et al. Potentially preventable prehospital deaths from motor vehicle collisions. *Traffic Inj Prev* 2016;17:676–80. doi: 10.1080/15389588.2016.1149580.

22. Schoeneberg C, Schilling M, Probst T et al. Preventable and potentially preventable deaths in severely injured elderly patients: A single-center retrospective data analysis of a german trauma center. *World J Surg* 2014;38:3125–32. doi: 10.1007/s00268-014- 2755-0.

23. Papadopoulos IN, Bukis D, Karalas E et al. Preventable prehospital trauma deaths in a Hellenic urban health region: an audit of prehospital trauma care. *J Trauma* 1996;41:864–9.

24. Harris T, Davenport R, Hurst T et al. Improving outcome in severe trauma: trauma systems and initial management--intubation, ventilation and resuscitation. *Postgrad Med Journal*, 2012;88:588–94. doi: 10.1136/postgradmedj-2010-74245.

25. Institute for Health Metrics and Evaluation (IHME). GBD Compare. GBD 2016.

For peer review only - http://bmjopen.bmj.com/site/about/guidelines.xhtml

https://vizhub.healthdata.org/gbd-compare/. Accessed February 18, 2018.

26. Abajobir AA, Abbafati C, Abbas K et al. Global, regional, and national age-sex specific mortality for 264 causes of death, 1980–2016: a systematic analysis for the Global Burden of Disease Study 2016. *Lancet* 2017;390:1151–210. doi: 10.1016/S0140- 6736(17)32152-9.

27.Pillay-van Wyk V, Msemburi W, Laubscher et al. Mortality trends and differentials in South Africa from 1997 to 2012: second National Burden of Disease Study. *Lancet Glob Health* 2016;4:e642–e653. doi: 10.1016/S2214-109X(16)30113-9.

28.Oliver GJ and Walter DP. A Call for Consensus on Methodology and Terminology to Improve Comparability in the Study of Preventable Prehospital Trauma Deaths: A Systematic Literature Review. *Acad Emerg Med* 2016;23:503–10. doi: 10.1111/acem.12932.

29. Zafarghandi MR, Modaghegh MHS, Boudsari BS. Preventable Trauma Death in Tehran: An Estimate of Trauma Care Quality in Teaching Hospitals. *J Trauma* 2003;55:459–65. doi: 10.1097/01.TA.0000027132.39340.FE.

30. Moon S, Lee SH, Ryoo HW et al. Preventable trauma death rate in Daegu, South Korea. *Clini Exp Emerg Med* 2015;2:236–43. doi: 10.15441/ceem.15.085.

31. Iau PTC, Ong CL, Chan STF. Preventable trauma deaths in Singapore. *Aust N Z J Surg* 1998;68:820–5. doi: 10.1046/j.1440-1622.1998.01461.x.

32. Chiara O, Cimbanassi S, Pitidis A et al. Preventable trauma deaths: from panel review to population based-studies. *World J Emerg Surg* 2006;1:1–7. doi: 10.1186/1749-7922-1- 12.

33. Kwon AM, Garbett NC, Kloecker GH. Pooled preventable death rates in trauma patients: Meta analysis and systematic review since 1990. *Eur J Trauma Emerg Surg* 2014;40:279– 85. doi: 10.1007/s00068-013-0364-5.

34. Settervall CHC, Domingues C. de A, Cardoso de Sousa RM et al. Preventable Trauma Deaths. *Rev Saude Publica*, 2012;46:1–8. doi: 10.1001/jama.1985.03360080071032.

35. Castillo-Rodríguez L, Díaz-Jiménez D, Castañeda-Orjuela C. et al. Years of Life Lost (YLL) in Colombia 1998-2011: Overall and avoidable causes of death analysis. *PLoS ONE* 2015;10:1–11. doi: 10.1371/journal.pone.0125456.

36. Henry JA, Reingold AL. Prehospital trauma systems reduce mortality in developing countries. *J Trauma Acute Care Surg* 2012;73:261–8. doi: 10.1097/TA.0b013e31824bde1e.

37. Montmany S, Pallisera A, Rebasa P et al. Preventable deaths and potentially preventable deaths. What are our errors? *Injury* 2016;47:669-73

38. Sugrue M, Caldwell E, D’Amours S et al. Time for a change in injury and trauma care

For peer review only - http://bmjopen.bmj.com/site/about/guidelines.xhtml

delivery: A trauma death review analysis. *Aust N Z J Surg* 2008;78:949–54. doi: 10.1111/j.1445-2197.2008.04711.x.

39. Vioque SM, Kim PK, McMaster J. Classifying errors in preventable and potentially preventable trauma deaths: A 9-year review using the Joint Commission's standardized methodology. *American Journal of Surgery* 2014;208:187–94. doi: 10.1016/j.amjsurg.2014.02.006.

40. Navarro S, Montmany S, Rebasa P. Impact of ATLS training on preventable and potentially preventable deaths. *World J Surg* 2014;38:2273–8. doi: 10.1007/s00268-014- 2587-y.

41. Ouma PO, Maina J, Thuranira PN. Access to emergency hospital care provided by the public sector in sub-Saharan Africa in 2015: a geocoded inventory and spatial analysis. *Lancet Glob Health* 2018;2214–109. doi: 10.1016/S2214-109X(17)30488-6.

42. Goosen J, Bowley DM, Degiannis E et al. Trauma care systems in South Africa. *Injury*, 2003;34:704–8. doi: 10.1016/S0020-1383(03)00153-0.

43. McSwain N, Rotondo M, Meade et al. A model for rural trauma care. *Br J Surg* 2012;99:309–14. doi: 10.1002/bjs.7734.

44. Hussain LM, Redmond AD. Are pre-hospital deaths from accidental injury preventable? *Br Med J (Clin Res Ed)* 1994;308:1077–80. doi: 10.1136/bmj.309.6946.57.

45. London JA, Mock CN, Quansah RE. Priorities for improving hospital-based trauma care in an African city. *J Trauma* 2001;51:747–53. doi: 10.1097/00005373-200110000-00021.

46. Berwick DM, Downey AS, Cornett EA. A National Trauma Care System to Achieve Zero Preventable Deaths After Injury Recommendations From a National Academies of Sciences, Engineering, and Medicine Report. *JAMA* 2016;316:927–28. doi: 10.1001/jama.2016.8524.Conflict.

For peer review only - http://bmjopen.bmj.com/site/about/guidelines.xhtml

For peer review only

For peer review only - http://bmjopen.bmj.com/site/about/guidelines.xhtml

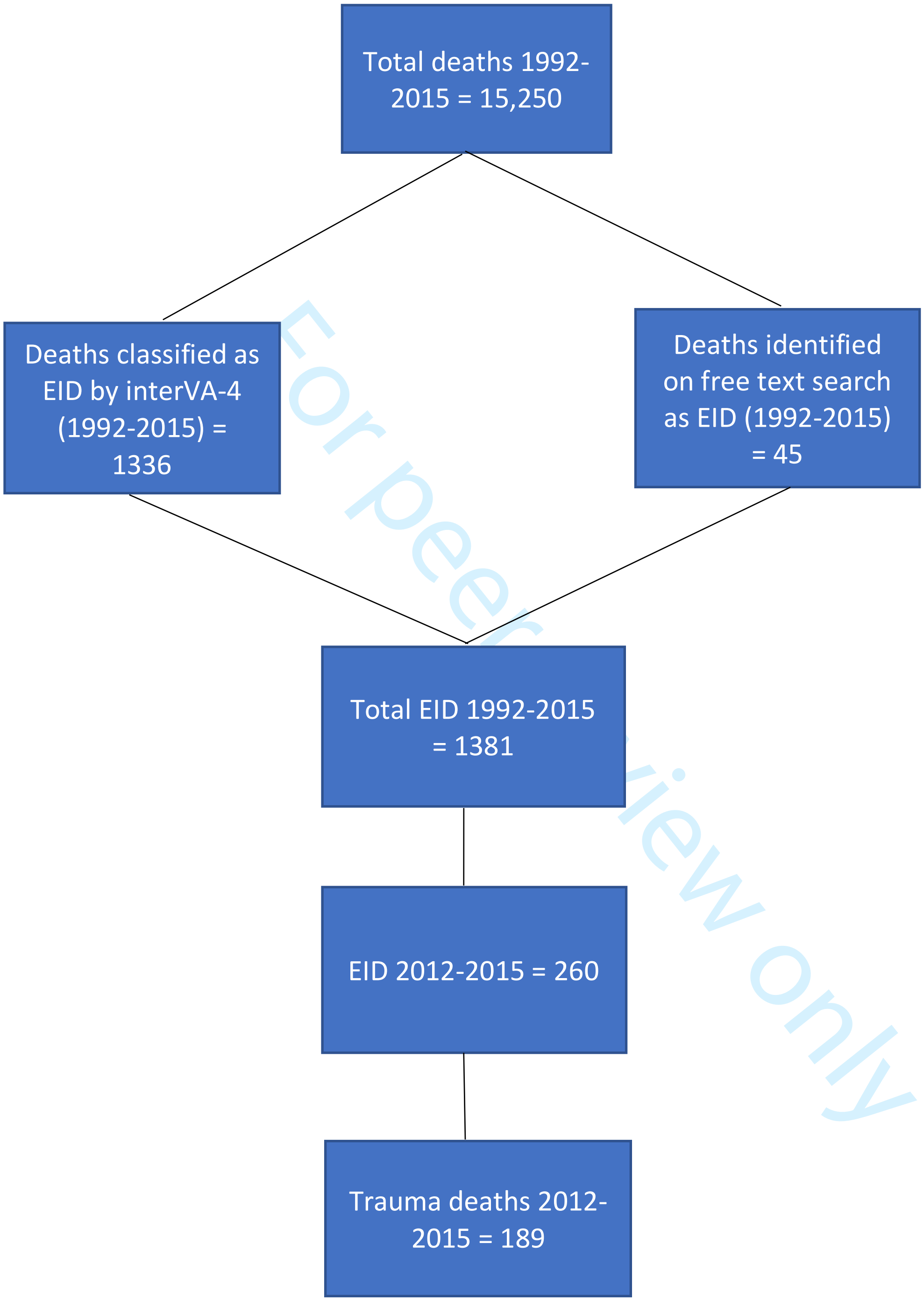

Figure 1. Deaths considered in this study (EIDs and trauma), relative to total deaths in the HDSS

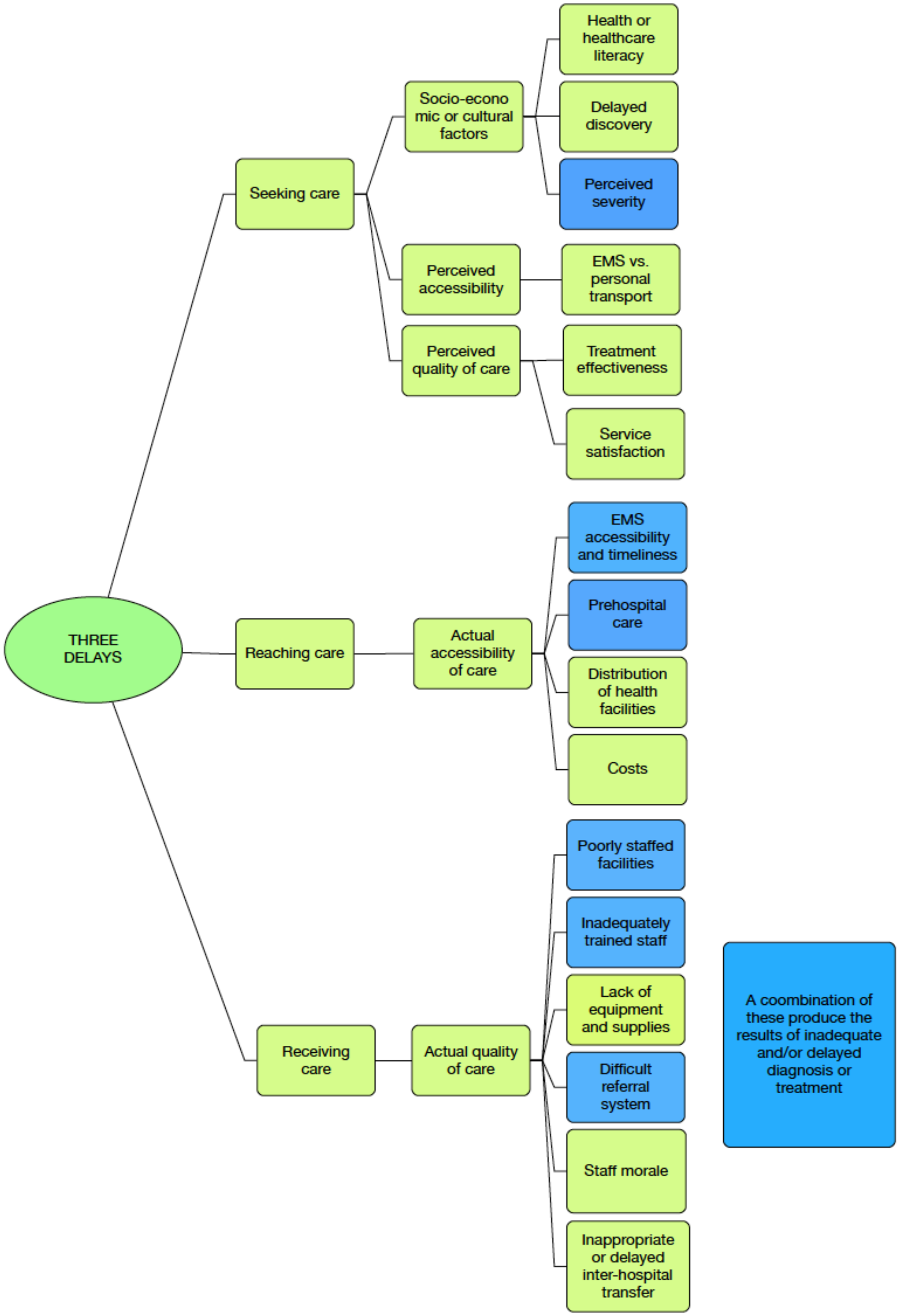

Figure 2. Composite three-delays framework for avoidable death in trauma.
*The most frequently cited factors from the VA analysis and KIIs analysis are shaded in blue.

Appendices

Appendix 1. Details of the definitions of injury survivability

DEFINITIONS OF SURVIVABILITY	STUDIES EXAMINING INJURY SURIVABILITY
Vitals at the scene of injury e.g. palpable pulse or blood pressure	[1]
Awake at the scene	[1-3]
Reversible or treatable injury	[4,3]
Generally stable at initial treatment or if unstable, becomes stable with initial treatment	[5]
Non-severe injuries	[3]
Non-severe single-system or single-organ or single-vessel injuries	[1,2]
Non-severe non-central nervous system (CNS) injuries	[3]
**APPROACHES TO CRITERIA FOR DEFINITION OF SURVIVABILITY**	**STUDIES**
Abbreviated Injury Scale (AIS)/Injury Severity Scores (ISS)/Probability of Survival (PS)/Trauma Score (TS)	[6-8]
AIS/ISS/PS and specific preventable factors	[9,10-12]
AIS/ISS/PS and impact of care delivered	[13-31]
Survivable injuries (subjective/panel)	[5]
Survivable injuries (subjective/panel) and impact of care delivered	[2,4,32-34,31,1,5,35,36,3]
Impact of care delivered	[37-38]

Appendix 2. Details of the three-delays model for care extracted from literature review*

DELAY	FACTORS AFFECTING UTILIZATION OR OUTCOME
DECIDING TO SEEK CARE	**Socio-economic/Cultural**<br>Illness factors<br>● Healthcare literacy<br>● Delayed discovery [4,14]<br>● Perceived severity<br>● Perceived etiology<br>Economic status [39]<br>Educational status
	**Perceived Accessibility**<br>Distance<br>Transportation [4]<br>● EMS versus personal transport**<br>● Road conditions<br>● Season—dry or rainy<br>Costs [39]<br>● Transportation<br>● Medical care<br>● Medications<br>● Opportunity costs<br>● Lost income<br>EMS transport protocols [39]<br>● Closest hospital versus best equipped hospital
	**Perceived quality of care**<br>Reputation or previous experience<br>Satisfaction with outcomes<br>● Treatment effectiveness<br>Satisfaction with service<br>● Staff attitudes<br>● Hospital capacity and surgery<br>● Supplies availability<br>● Waiting time<br>● Hospital efficiency<br>● Care consistent with local beliefs<br>● Timely care<br>● Visitation rules affecting social support
IDENTIFYING AND REACHING CARE	**Actual accessibility of care**<br>Distribution of health facilities [31,37,39]<br>EMS accessibility and timeliness of response [2,37]<br>Travel time [1,40,4,14]<br>Prehospital care [33,41,42,37,39]<br>Costs

For peer review only

	EMS transport protocols<br>● Closest hospital versus most equipped hospital [43]
RECEIVING ADEQUATE AND APPROPRIATE TREATMENT	**Actual quality of care**<br>Poorly staffed facilities [33]<br>● Number of staff/lack of personnel<br>● Competence of staff—training of personnel [40,20]<br>● Maldistribution of staff<br>● Lack of managers<br>Poorly equipped facilities [33,39]<br>● Lack of a blood bank<br>● Lack of a functioning operating room (OR)<br>● Lack of medications or supplies<br>● Lack of infrastructure<br>Inadequate management [33,2,4,20,21,31,42,43,22,37,39,27]<br>● Inadequate or delayed diagnosis<br>● Inadequate or delayed treatment<br>● Inappropriate or delayed inter-hospital transfer<br>● Functioning trauma system<br>● Ward care after ER treatment

*Model based on that created for obstetric emergencies (Thaddeus and Maine, 1994). Delay factors examined in other studies, with respect to trauma, are cited here.

**EMS refers not only to the mode of transportation, but also to the care provided while in transport. As perceptions of EMS can affect decisions to seek care, EMS appears in both seeking care and reaching care categories.

For peer review only

Appendix 3. References for articles in literature review.

1. MacLeod JBA, Cohn SM, Johnson EW et al. Trauma deaths in the first hour: are they all unsalvageable injuries? *Am J Surg* 2007;193:195–9. doi: 10.1016/j.amjsurg.2006.09.010.
2. Kleber C, Giesecke MT, Tsokos M et al. Trauma-related preventable deaths in Berlin 2010: Need to change prehospital management strategies and trauma management education. *World J Surg* 2013;37: 1154–61. doi: 10.1007/s00268-013-1964-2.
3. Ray JJ, Meizoso JP, Satahoo SS et al. Potentially preventable prehospital deaths from motor vehicle collisions. *Traffic Inj Prev* 2016;17:676–80. doi: 10.1080/15389588.2016.1149580.
4. Papadopoulos IN, Bukis D, Karalas E et al. Preventable prehospital trauma deaths in a Hellenic urban health region: an audit of prehospital trauma care. *J Trauma* 1996;41:864–9.
5. Schoeneberg C, Schilling M, Probst T et al. Preventable and potentially preventable deaths in severely injured elderly patients: A single-center retrospective data analysis of a german trauma center. *World J Surg* 2014;38:3125–32. doi: 10.1007/s00268-014- 2755-0.
6. Boman H, Bjornstig U, Hedelin A et al. Avoidable deaths in two areas of Sweden--analysis of deaths in hospital after injury. *European Journal of Surgery* 1999;165:828-33
7. Pellicane J, Byrnne K, DeMaria E. Preventable complications and death from multiple organ failure among geriatric trauma victims. *J Trauma* 1992;33:440-44
8. Oliver GJ, Walter DP, Redmond AD. Prehospital deaths from trauma: are injuries survivable and do bystanders help? *Injury* 2017;48:985-91
9. Hussain LM, Redmond AD. Are pre-hospital deaths from accidental injury preventable? *Br Med J (Clin Res Ed)* 1994;308:1077–80. doi: 10.1136/bmj.309.6946.57.
10. Diamond I, Parkin P, Wales P et al. Preventable pediatric deaths in Ontario: a comparative population-based study. *J Trauma* 2009;66:1189-94.
11. Lerer L, Knottebnelt J. Preventable mortality following sharp penetrating chest trauma. *J Trauma* 1994;37:9-12
12. Oliver GJ, Walter DP, Redmond AD. Are prehospital deaths from trauma and accidental injury preventable? A direct historical comparison to assess what has changed in two decades. *Injury* 2017;48:978-84
13. McDermott F, Cordner S, Tremayne A. Evaluation of the medical management and preventability of death in 137 road traffic fatalities in Victoria, Australia: an overview. Consultative Committee on Road Traffic Fatalities in Victoria. *J Trauma* 1996;40:520-33.
14. Esposito T, Sanddal N, Hansen J et al. Analysis of preventable trauma deaths and inappropriate trauma care in a rural state. *J Trauma* 1995;39:955-62.
15. Esposito T, Sanddal N, Dean J et al. Analysis of preventable pediatric trauma deaths and inappropriate trauma care in Montanna. *J Trauma* 1999;47:243-51.

16. Esposito T, Sanddal T, Reynolds S et al Effect of a voluntary trauma system on preventable death and inappropriate care in a rural state. *J Trauma* 2003;54:663-70.

17. Henriksson E, Ostrom M, Eriksson A. Preventability of vehicle-related fatalities. *Accid Anal Prev* 2001;33:467-75.
18. Rautji R, Bhardwaj D, Dogra T. The Abbreviated Injury Scale and its correlation with preventable traumatic accidental deaths: A study from South Delhi. *Medicine Science and the Law* 2006;46:157-65
19. Chiara O, Scott J, Cimbanassi S et al. Trauma deaths in an Italian urban area: An audit of prehospital and in-hospital trauma care. *Injury* 2002;33:553-62
20. Vioque SM, Kim PK, McMaster J. Classifying errors in preventable and potentially preventable trauma deaths: A 9-year review using the Joint Commission's standardized methodology. *American Journal of Surgery* 2014;208:187–94. doi: 10.1016/j.amjsurg.2014.02.006.
21. Montmany S, Pallisera A, Rebasa P et al. Preventable deaths and potentially preventable deaths. What are our errors? *Injury* 2016;47:669-73
22. Saltzherr T, Wendt K, Nieboer P et al. Preventabilityof trauma deaths in a Dutch level-1 trauma centre. *Injury* 2011;42:870-73.
23. Motomura T, Mashiko K, Matsumoto H et al. Preventable trauma deaths after traffic accidents in Chiba Prefecture, Japan, 2011: Problems and Solutions. *Journal of Nippon Medical School* 2014;81:320-27
24. Sampalis J, Boukas S, Nikolis A et al. Preventable death classification: Inter-rater reliability and comparison with ISS-based survival probability estimates. *Accident Analysis and Prevention* 1995;27:199-206.
25. Schoeneberg C, Schilling M, Hussmann B et al. Preventable and potentially preventable deaths in severely injured patients: a retrospective analysis including patterns of errors. *Eur J Trauma Emerg Surg* 2016;1-9
26. Moon S, Lee SH, Ryoo HW et al. Preventable trauma death rate in Daegu, South Korea. *Clini Exp Emerg Med* 2015;2:236–43. doi: 10.15441/ceem.15.085.
27. Ha M, Kim B, Choi S et al. Preventable and potentially preventable traumatic death rates in a neurosurgery department: a single centre experience. *Korean J Neurotrauma* 2016;12:67-71
28. Kuo L, Kaufman E, Hoffman R et al. Failure to rescue after injury is associated with preventability: the results of mortality panel review of failure to rescue cases in trauma. *Surgery* 2017;161:782-90.
29. 65. Kim Y, Jung K. Utility of the international classification of diseases injury severity score: detecting preventable deaths and comparing the performance of emergency medical centres. *J Trauma* 2003;54:775-80.
30. Iau PTC, Ong CL, Chan STF. Preventable trauma deaths in Singapore. *Aust N Z J Surg* 1998;68:820–5. doi: 10.1046/j.1440-1622.1998.01461.x.
31. Sugrue M, Caldwell E, D'Amours S et al. Time for a change in injury and trauma care delivery: A trauma death review analysis. *Aust N Z J Surg* 2008;78:949–54. doi: 10.1111/j.1445-2197.2008.04711.x.

For peer review only

32. Sanddal T, Esposito T, Whitney J et al. Analysis of preventable trauma deaths and opportunities for trauma care improvement in Utah. *J Trauma* 2011;70:970-77
33. Yeboah D, Mock C, Karikari P et al. Minimizing preventable trauma deaths in a limited-resource setting: A test-case of a multidisciplinary panel review approach at the Komfo Anokye Teaching Hospital in Ghana. *World J Surg* 2014;38:1707-12. doi: 10.1007/s00268-014-2452-z.
34. Zafarghandi MR, Modaghegh MHS, Boudsari BS. Preventable Trauma Death in Tehran: An Estimate of Trauma Care Quality in Teaching Hospitals. *J Trauma* 2003;55:459–65. doi: 10.1097/01.TA.0000027132.39340.FE.
35. Maxson T, Mabry C, Sutherland M. Does the institution of a statewide trauma system reduce preventable mortality and yield a positive return on investmnt for taxpayers. *J Am Coll Surg* 2017;224:489-99
36. Navarro S, Montmany S, Rebasa P. Impact of ATLS training on preventable and potentially preventable deaths. *World J Surg* 2014;38:2273–8. doi: 10.1007/s00268-014-2587-y.
37. Lauriola P, Tosatti F, Schiavi A et al. Confidential enquiry into avoidable vehicle accidental deaths in the province of Modena, Italy. *Eur J Epidemiol*, 2000;16:67-74.
38. Stocchetti N. Risk prevention, avoidable deaths and mortality-morbidity reduction in head injury. *Eur J Emerg Med* 2001;8:215-219.
39. Simons R, Brasher P, Taulu T et al. A population-based analysis of injury-related deaths and access to trauma care in rural-remote Northwest British Columbia. *J Trauma* 2010;69:11-9.
40. Chiara O, Cimbanassi S, Pitidis A et al. Preventable trauma deaths: from panel review to population based-studies. *World J Emerg Surg* 2006;1:1–7. doi: 10.1186/1749-7922-1-12.
41. Henry JA, Reingold AL. Prehospital trauma systems reduce mortality in developing countries. *J Trauma Acute Care Surg* 2012;73:261–8. doi: 10.1097/TA.0b013e31824bde1e.
42. Goosen J, Bowley DM, Degiannis E et al. Trauma care systems in South Africa. *Injury*, 2003;34:704–8. doi: 10.1016/S0020-1383(03)00153-0.
43. McSwain N, Rotondo M, Meade et al. A model for rural trauma care. *Br J Surg* 2012;99:309–14. doi: 10.1002/bjs.7734.

For peer review only

Appendix 4. Distribution of cause of death categories for EIDs, trauma, avoidable EIDs and avoidable trauma deaths, 2012-2015.

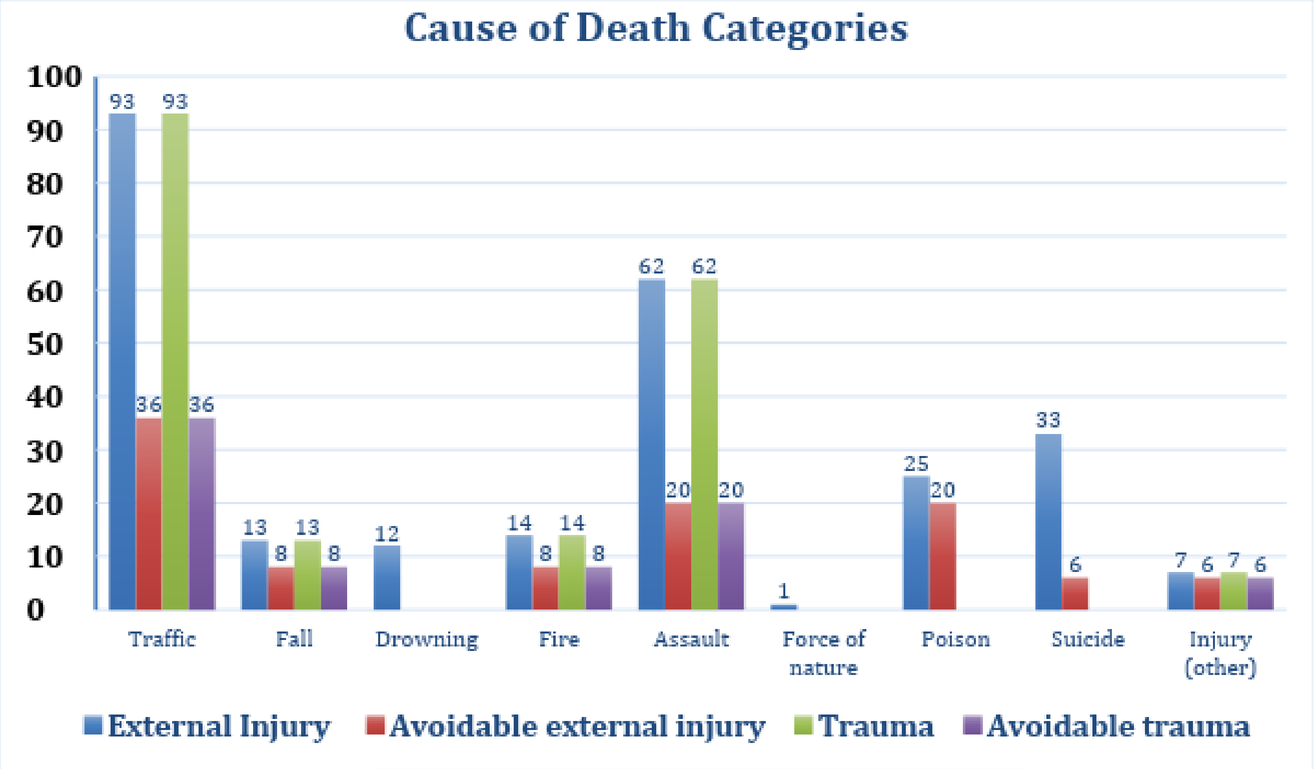

Injury (other) category reflects injuries from objects hitting patients e.g. a football.

Appendix 5. Details of the circumstances of mortality module for the avoidable EIDs (n=109) and trauma (n=81), 2012-2015

**Social module category**	**Indicator**	**EIDs n (%)**	**Trauma n (%)**
Recognition of severity	Doubts about the need for care	0 (0)	0(0)
	Use of traditional medicine	1(1.0)	1 (1.3)
Mobilising assets to seek care	Did not use a mobile phone to call for help	27 (26)	21 (27)
	Did not use motorised transport to get to care	24 (23)	18 (23)
Access to care	Prohibitive costs	21 (20)	16 (21)
	Did not travel to a hospital or health facility	29 (28)	21 (27)
	>2 hrs travel to care	0 (0)	0 (0)
Quality of care	Problems with admission	2 (1.9)	2 (2.6)
	Problems with treatment (medical treatment, procedures, interpersonal attitudes, respect, dignity)	9 (8.6)	8 (10.2)
	Problems with tests and medications	6 (7.5)	6 (10.2)

Note, for the quality of care indicators and "did not use motorized transport to get to care", n=80 for avoidable EIDs and n=59 for avoidable trauma deaths. This reflects the fact that the denominator in these cases is the total number of eopoe who traveled to hospital

For peer review only

Appendix 6. Details of the key informant interview results.

Seeking care

Health or healthcare literacy

For the general population, HCPs felt that most patients were unaware of proper channels for seeking care, especially when an injury was not acutely severe.

> *"So, there are those who say straight to the hospital and those who say no but you can never actually tell which one because they don't know themselves…it's just a matter of their specific attitude, and there are mistakes in both ways …"*—Medical officer

EMS vs. personal transport

HCPs know that patients are aware of delayed responses from EMS services, so most patients decide between waiting for EMS and seeking personal transport. Previous experience with EMS delays may affect the decision to seek care, as some patients will decide to seek care only when personal transport can be arranged. For those who can afford private ambulance services, this is an easier decision to make, as the private ambulances arrive earlier and are better equipped.

> *"From what we've heard, it's delayed response from the ambulances, the emergency services…the emergency service…it's a problem… and also for the patients, I think because this place is surrounded by villages…if they don't have transport, their own transport to come here, it becomes a bit of a challenge"* –Clinical associate

Mistrust of clinics

Although there may be clinics available, they are not open outside business hours and not well equipped, so patients often decide to wait in the Casualty department of district hospitals, even when their conditions may have been treated in clinics. Although seeking any kind of care after an injury is better than seeking no care at all, for most injuries, seeking care at a hospital may be more appropriate than seeking care in a clinic.

> *"…the patients …they come with their referral, but you will find that most of them don't have a referral, which means that they don't trust their clinic, the clinic for them. Even those cases that can be seen there, but most of the time, they are not seen there."* –Medical officer

> *"They don't really like going to the clinics, because to them, they feel like, they're going to send me to the hospital anyways, so they prefer coming straight to the hospitals, because they feel like they're wasting time."* –Medical officer

Reaching care

EMS accessibility and timeliness

The HCPs felt that the largest barrier for patients reaching care was EMS accessibility. Given the rural area and the limited number of ambulances, patients often wait a long time to be taken to hospital.

For peer review only - http://bmjopen.bmj.com/site/about/guidelines.xhtml

> *"… the biggest problem I think they have is access to emergency care personnel, the paramedics, the ambulance. People may have trauma cases and when they call, the ambulance is already out, attending to trauma care somewhere else. It might take two to three hours for this ambulance to come and pick this patient. Now that is just too long for some cases. That might mean the difference between being normal again and being invalid or even dead. So, that is it. It is just the availability of the service…it's not optimal."*—Medical officer

Prehospital care

As well as the issues with EMS timeliness, the care provided by EMS providers is limited by the training they receive, as well as the lack of equipment to provide early resuscitation.

> *"It's not only death, we can also prevent the sequelae people come in with…they get brain damage, because the care was too late. The thing of the transport is very important, because the other guys, the EMS, if something happens, they are not well trained to resuscitate those patients. I've seen people die here because of that."* – Medical officer

<u>Receiving care</u>

Poorly staffed facilities

In the district hospitals, there is a shortage of staff to provide care. The Casualty departments are largely run by clinical associates or junior doctors, who sometimes have difficulty getting senior supervision. Also, in the regional hospital, there is one orthopaedic surgeon and one general surgeon. In the tertiary hospital, there are more orthopaedic and general surgeons but only one neurosurgeon. When these experts are not available, care for the patients is severely delayed.

> *"From my side, as a clinical associate, some of the patients, you need a second opinion from a doctor or something like that, but it is almost impossible, if not impossible, to get that person whom you can talk to about the patient. So, that's the biggest challenge."*—Clinical associate

Inadequately trained staff

An important recurring theme was the need for more training. Many HCPs did not receive standardized training in trauma care e.g. ATLS courses and those that did expressed the lack of continued learning. Not only are there barriers of poor staffing that limit the ability to explore training options located in other provinces, but there are also financial barriers, as HCPs said they financed the course and travel costs independently.

> *"There is no specific ATLS course given to us, actually in the province as a whole. I understand that in Guateng, and in Limpopo maybe. In other hospitals, they send their doctors, especially their junior doctors, to other places, to do their ATLS training, but with us, there is not that. So, you have to be organized and find out when an ATLS course is going to be, find out for yourself and pay for yourself. Which is not convenient also for the hospital … you cannot go away for a week … how many of us… let's say it's me and my colleague who decide to go. That means for that week, we will leave a hole*

in the hospital, leave people struggling here, while you are out to educate yourself. Yes, so it is not the best."—Medical officer

"…now after you've stabilized your patient and there is another trauma case, you have to take your team with you, but someone has to stay and take care of the first patient and now you're faced with a situation where you have to give the most junior person the responsibility of watching the vitals, but they don't really know what they are watching and the next thing you know, the machine alarms go off … they do not know what they are watching… they do not know that the [oxygen] saturation is not supposed to drop below 85% and then eventually you find yourself certifying a patient who … (heavy sigh)… you see what I mean?"—Medical officer

Lack of equipment and supplies

Given financial constraints, there is often a lack of important medical equipment and supplies. When they are present, they easily become non-functional and are not repaired or replaced on time.

"…the second problem is life support equipment. Today it is there, tomorrow, it is broken down, yes …there is a blood corner, where we keep emergency blood, maybe two or three units in a fridge."—Medical officer

Difficult referral system

A major problem in the care of severely injured patients, who are most commonly neurotrauma patients, is the ability to transfer them to a tertiary care institution, which is the only location for a computed tomography (CT) scan and access to a neurosurgeon. Some of the barriers include miscommunication about which hospitals receive referrals from the district hospitals and limited number of ambulances, especially ICU ambulances.

"the problem with serious trauma is the referral. When you have to refer to a higher institution, it's a hassle, it's a problem"—Clinical associate

"Ok, uhh, the first, the big barrier, is the referral system. I can have a patient here, head injury, low GCS, they bring the patient in respiratory distress. I'll try to stabilize the patient. I'll try to stop the bleeding, stabilize the patient, tube the patient, and then…I'll get the doctors at the higher level, they will accept the patient, but to transfer that patient, I need an ICU ambulance but in the province, there are only two ICU ambulances. So, the ICU ambulance probably has to leave Witbank to come here, maybe it can take three to four hours. Then take the patient from here to Nelspruit, that is six hours, then it is a problem…especially since we don't have good ventilation equipment."—Medical officer

"Well, sometimes, it's difficult because our referral system is contradictory. Like last week, we were surprised that our referrals should be going to Themba hospital. Meanwhile, we have been arranging them with Mapulaneng …They said that memo had been written in March, but we didn't know about it. Now, when we communicate with Themba, like last night, the doctor on call was trying to arrange with Themba for

For peer review only

an acute abdomen…but they wanted him to send to Mapulaneng…it's still a bit of a confusion. It's difficult."—Medical officer

Staff morale

Given the numerous challenges described above, the HCPs described feelings of frustration and disappointment with the amount of care they could provide, especially knowing that in better circumstances, they could save more lives.

> *"If you get an ICU ambulance, you have to get the intermediate trained ICU paramedics, or you have to get a chopper and that takes long so honestly, there is only so much you can do here. Because after you've given your drugs and you've intubated and ventilated, what more can you do? You're just sitting there and the CSF is just leaking and then 6 hours later, they come and you know, come on, you think, why are we even doing this? Then they go there, they do the CT scan and then they find that there is no neurosurgeon on call, so what do they do now? They just send the patient back. So, in terms of trauma, we still have a long way to go."*—Medical officer

For peer review only

STROBE Statement—Checklist of items that should be included in reports of ***cross-sectional studies***

	Item No	Recommendation	Page No
**Title and abstract**	1	(*a*) Indicate the study's design with a commonly used term in the title or the abstract	1
		(*b*) Provide in the abstract an informative and balanced summary of what was done and what was found	2
**Introduction**			
Background/rationale	2	Explain the scientific background and rationale for the investigation being reported	4
Objectives	3	State specific objectives, including any prespecified hypotheses	4
**Methods**			
Study design	4	Present key elements of study design early in the paper	4
Setting	5	Describe the setting, locations, and relevant dates, including periods of recruitment, exposure, follow-up, and data collection	5
Participants	6	(*a*) Give the eligibility criteria, and the sources and methods of selection of participants	5,6
Variables	7	Clearly define all outcomes, exposures, predictors, potential confounders, and effect modifiers. Give diagnostic criteria, if applicable	5,6
Data sources/ measurement	8*	For each variable of interest, give sources of data and details of methods of assessment (measurement). Describe comparability of assessment methods if there is more than one group	*5,6*
Bias	9	Describe any efforts to address potential sources of bias	5,6
Study size	10	Explain how the study size was arrived at	5,6
Quantitative variables	11	Explain how quantitative variables were handled in the analyses. If applicable, describe which groupings were chosen and why	6
Statistical methods	12	(*a*) Describe all statistical methods, including those used to control for confounding	6
		(*b*) Describe any methods used to examine subgroups and interactions	-
		(*c*) Explain how missing data were addressed	5
		(*d*) If applicable, describe analytical methods taking account of sampling strategy	6
		(*e*) Describe any sensitivity analyses	-
**Results**			
Participants	13*	(a) Report numbers of individuals at each stage of study—eg numbers potentially eligible, examined for eligibility, confirmed eligible, included in the study, completing follow-up, and analysed	8,13
		(b) Give reasons for non-participation at each stage	-
		(c) Consider use of a flow diagram	Fig1
Descriptive data	14*	(a) Give characteristics of study participants (eg demographic, clinical, social) and information on exposures and potential confounders	8-13
		(b) Indicate number of participants with missing data for each variable of interest	-
Outcome data	15*	Report numbers of outcome events or summary measures	8-12
Main results	16	(*a*) Give unadjusted estimates and, if applicable, confounder-adjusted estimates and their precision (eg, 95% confidence interval). Make clear which confounders were adjusted for and why they were included	-

For peer review only - http://bmjopen.bmj.com/site/about/guidelines.xhtml

		(*b*) Report category boundaries when continuous variables were categorized	-
		(*c*) If relevant, consider translating estimates of relative risk into absolute risk for a meaningful time period	-
Other analyses	17	Report other analyses done—eg analyses of subgroups and interactions, and sensitivity analyses	-
**Discussion**			
Key results	18	Summarise key results with reference to study objectives	15
Limitations	19	Discuss limitations of the study, taking into account sources of potential bias or imprecision. Discuss both direction and magnitude of any potential bias	17
Interpretation	20	Give a cautious overall interpretation of results considering objectives, limitations, multiplicity of analyses, results from similar studies, and other relevant evidence	15-16
Generalisability	21	Discuss the generalisability (external validity) of the study results	15,17
**Other information**			
Funding	22	Give the source of funding and the role of the funders for the present study and, if applicable, for the original study on which the present article is based	18

*Give information separately for exposed and unexposed groups.

Note: An Explanation and Elaboration article discusses each checklist item and gives methodological background and published examples of transparent reporting. The STROBE checklist is best used in conjunction with this article (freely available on the Web sites of PLoS Medicine at http://www.plosmedicine.org/, Annals of Internal Medicine at http://www.annals.org/, and Epidemiology at http://www.epidem.com/). Information on the STROBE Initiative is available at www.strobe-statement.org.

For peer review only

For peer review only - http://bmjopen.bmj.com/site/about/guidelines.xhtml
